# Supplementary material for: Discharge communication study: a realist evaluation of discharge communication experiences of patients, general practitioners and hospital practitioners, alongside a corresponding discharge letter sample
Source: BMJ Open. 2021 Jul 21;11(7):e045465. doi: 10.1136/bmjopen-2020-045465 (PMC8296817; doi:10.1136/bmjopen-2020-045465)
Supplement: Supplementary data [file bmjopen-2020-045465supp001.pdf]

No. of letters selected	Patient name (to be removed during redaction)	Patient Unique research ID (to be added during redaction)	Categorisation (**Unsuccessful** OR **successful** discharge letter example)	Reason for selection & categorisation (e.g. any key good or bad points about letter)
EXAMPLE (Before redaction)<br><br>*(after redaction)*	*Mr Joe Smith*<br><br>[redacted]	 <br>*P0001*	*Unsuccessful*<br><br>*Unsuccessful*	*Bad points:*<br>*Medication alterations poorly outlined and information given to patient not explained*
1				
2				
3				

More rows to be added as needed…

*This grid has been previously published [(1)] under a CC-BY license and has been re-produced here for ease of reference for readers.

1.Weetman K., Spencer R., Dale J., *et al.* What makes a "successful" or "unsuccessful" discharge letter? Hospital clinician and General Practitioner assessments of the quality of discharge letters. *BMC Health Services Research*. 2021;21(1):349. https://doi.org/10.1186/s12913-021-06345-z [Accessed: 18/05/21].
