# Supplementary material for: Discharge communication study: a realist evaluation of discharge communication experiences of patients, general practitioners and hospital practitioners, alongside a corresponding discharge letter sample
Source: BMJ Open. 2021 Jul 21;11(7):e045465. doi: 10.1136/bmjopen-2020-045465 (PMC8296817; doi:10.1136/bmjopen-2020-045465)
Supplement: Supplementary data [file bmjopen-2020-045465supp002.pdf]

GP interview and focus group guide

Interviewer opening question:

Please tell me about your experience(s) of patients receiving written discharge communication?

The rest of interview or focus group will continue in a conversational manner discussing GPs views and experiences on patients receiving written discharge communication and how the discharge communication process can be improved.

Possible interviewer prompts:

- What are your experiences of discharge communication as a GP?
- How do you think discharge communication can be improved?
- Please tell me your views on the discharge letters you selected?
- How would you suggest to improve these letters?
- In your opinions, is this letter suitable for a/the patient?
- What are your views on patients receiving letters?
- What do you think are important content items for good quality discharge letters?
- In your view what are the effects and outcomes of poor quality discharge letters?

*This guide has been previously published [1] under a CC-BY license and has been re-produced here for ease of reference for readers.

1.Weetman K., Dale J., Scott E., *et al.* The Discharge Communication Study: research protocol for a mixed methods study to investigate and triangulate discharge communication experiences of patients, GPs, and hospital professionals, alongside a corresponding discharge letter sample. *BMC Health Services Research*. 2019;19(1):825. https://doi.org/10.1186/s12913-019-4612-1 [Accessed: 10/06/20].
