# Supplementary material for: Discharge communication study: a realist evaluation of discharge communication experiences of patients, general practitioners and hospital practitioners, alongside a corresponding discharge letter sample
Source: BMJ Open. 2021 Jul 21;11(7):e045465. doi: 10.1136/bmjopen-2020-045465 (PMC8296817; doi:10.1136/bmjopen-2020-045465)
Supplement: Supplementary data [file bmjopen-2020-045465supp003.pdf]

Patient interview schedule

I: Interviewer (member of the research team) *Action points Q= Question

I: ***Q1: Please tell me about your experiences of receiving any form of written discharge communication? This can be either a direct copy of the letter sent to your GP or a discharge letter specifically addressed to yourself.***

Q2: When you were discharged from hospital on DATE, what information were your given?

****if patient able to be shown letter copy as per protocol, show patient their letter****

Q3: How did you feel about the information you were given?

Q4: What written information would you like to be given or sent when being discharged from hospital and why?

Q5: Would you prefer to receive a direct copy of the letter sent to your GP or a separate letter specifically addressed to yourself?

Q6: Would you like to always be given this letter or would you prefer to choose each time you are discharged?

Q7: How do you think the process of patients receiving written discharge communication can be improved?

Q8: Is there anything else you would like to talk to me about today related to written discharge communication?

Discussion may continue in a relaxed conversational manner and researcher may ask additional questions related to anything else relevant mentioned by the patient.

*This guide has been previously published [1] under a CC-BY license and has been re-produced here for ease of reference for readers.

1. Weetman K., Dale J., Scott E., *et al.* The Discharge Communication Study: research protocol for a mixed methods study to investigate and triangulate discharge communication experiences of patients, GPs, and hospital professionals, alongside a corresponding discharge letter sample. *BMC Health Services Research*. 2019;19(1):825. https://doi.org/10.1186/s12913-019-4612-1 [Accessed: 10/06/20].
