# Supplementary material for: Discharge communication study: a realist evaluation of discharge communication experiences of patients, general practitioners and hospital practitioners, alongside a corresponding discharge letter sample
Source: BMJ Open. 2021 Jul 21;11(7):e045465. doi: 10.1136/bmjopen-2020-045465 (PMC8296817; doi:10.1136/bmjopen-2020-045465)
Supplement: Supplementary data [file bmjopen-2020-045465supp004.pdf]

BMJ Publishing Group Limited (BMJ) disclaims all liability and responsibility arising from any reliance placed on this supplemental material which has been supplied by the author(s)


## Trio meta-matrix with narrative summaries (S=successful, US=unsuccessful)

| Trio case | GP grading | GP comment and interview/focus group findings | Patient interview findings | HP survey findings | Summary of main trio findings |
|---|---|---|---|---|---|
| 1 | S | Although GP graded letter successful due to clear diagnosis and findings, they did comment that the patient management plan was unclear. GP asserted that they felt patients should receive letters as it informs the patient and is a "safety net" for ensuring follow up plans are actioned. | | HP gave letter high quality score of "9/9" and 9s in all other areas including GP care management plan except HP gave letter "4/9" for patient comprehensibility. HP concern that patients receiving letters may cause anxiety and distress. HP answered that it would be more appropriate for patients to receive personalised letters. | Although letter graded successful, GP did identify issues. Letter given a top score of "9" by HP. GP and HP appear to have differing views on whether patients should receive copies of their discharge letters with HP expressing concern and GP focussing on benefits. |
| 2 | S | | Patient generally pleased with discharge experience and happy to have received copy of the letter. Patient likes to be informed. Patient suggests some issues with understanding medical terminology and says that they would prefer to receive patient personalised letter. Patient would prefer choice of receiving letter at discharge. | HP gave overall quality score of "7/9" and patient comprehensibility score of "9/9". HP reports to always copy patients into letters and believes patients should have choice of receiving letters. Answers that patients should receive GP copy of discharge letter. | HP and patient agree about patients receiving letters but appear to disagree over the form that this should take – patient favours personalised correspondence whereas HP favours patients receiving copies of what is sent to the GP. |

| Trio case | GP grading | GP comment and interview/focus group findings | Patient interview findings | HP survey findings | Summary of main trio findings |
|---|---|---|---|---|---|
| 3 | S | | Patient overall seems pleased with communication and adds that they were given written and verbal information but only as they asked for a copy of the written information and that this was obtained after discharge. Patient describes follow up information in letter is unclear. Patient happy to receive copy of what GP receives and thinks it is reassuring to view the correspondence between doctors for transparency. Patient would prefer more detailed explanations in letter. | HP gives quality score of "8/9" with patient comprehensibility score of "9/9". Answers that patients should receive personalised letters and that patients should be given a choice. HP reports that despite hospital policy and their views on patient choice, they have never given a patient a discharge letter copy. HP believes that part of discharge letter should be given to patient and this is what is meant by personalised, not for two summaries to be generated. | HP given letter top score for patient comprehensibility but patient does report some issues and possible improvements which could be made to letter. Patient and HP in agreement over patient choice of receiving letters but disagreement over form. |
| 4 | S | | Patient says they were impressed with information provided; they were given a discharge letter copy. Patient thinks patients should receive letters automatically. | HP gave overall letter score of "9/9" and patient comprehensibility score of "9/9". HP reports to give patients letters most of the time and thinks patients should receive GP copy in opt out style system. | Broad agreement between HP and patient within this trio case. |
| 5 | US | Unclear procedure due to acronyms not comprehensible to GP; for this reason, unclear what had been done. GP thinks abbreviations should be written out in full for clarity both for the sake of the patient and themselves. | Patient received letter after long discharge delay in hospital. Patient pleased to have received letter. Patient says they cannot understand all of letter but that they are aware they can ask the GP if they want to understand more. | | Patient assumes GP understands all of letter and is a source of information for interpretation when GP does not due to use of uncommon abbreviations in letter. |

| Trio case | GP grading | GP comment and interview/focus group findings | Patient interview findings | HP survey findings | Summary of main trio findings |
|---|---|---|---|---|---|
| 6 | S | GP considered letter successful as follow up arranged. GP perceives use of acronyms in letter probably not comprehensible to patient. GP thinks use of lay terms in letter may be useful for patient understanding. | Patient thinks letter should ideally be emailed. Patient reports not being given much information and only received letter as relative went to hospital to get a copy after discharge. Patient feels discharge is not always clear and more time needs to be put in to ensure patient understanding. Patient felt letter generally inadequate and unsure of some of medical terms and acronyms in letter, patient states acronyms should not be used and terminology should be explained in lay terms. | | GP and patient in agreement that letter format not entirely accessible to patient. Agreement over ways to rectify this issue through avoidance of acronyms and explanations of medical terminology in lay terms. |
| 7 | S | Letter graded successful as follow up clear. GP perceives letter written in patient friendly language. | Patient reports no difficulties with letter understanding but does note inaccuracies in letter. | | GP and patient appear to agree on patient understanding. |
| 8 | US | Letter graded unsuccessful as drug changes and reasons for these unclear. | Patient reports being very pleased to have received copy of discharge letter having been given limited information in regard to previous discharges. Patient felt receiving letter supported their wellbeing. Patient conveys that receiving letter means that they can be actively involved in their own care and thus increase patient autonomy. | | Patients receiving letters may support and improve patient wellbeing. |
| 9 | S | GP graded letter successful as it gave full details of investigations and findings and a working diagnosis. Important in GP view for patient to be given plan of action and instructions. | Patient reports not to have been given a copy of the letter. Patient would have preferred to have been given written information to ensure that they do not forget anything. | | Patient and GP in agreement that patient did not receive a Letter and both appear to support practice of patients receiving letters. |

| Trio case | GP grading | GP comment and interview/focus group findings | Patient interview findings | HP survey findings | Summary of main trio findings |
|---|---|---|---|---|---|
| 10 | S | Letter graded successful as clear notes. Generally, letter informative and clear. GP raises possible issues with patient understanding due to presence of jargon and abbreviations; GP notes some patients would be fine with not understanding these elements whereas some patients will want to know more and may bring letter to GP with queries. GP says that there is a certain amount of technical information that needs to be passed between doctors but to improve patient understanding the letter should be clear and concise with use of lay language. | Patient given a copy of the letter. Patient reports medication information is very useful and clear but notes some issues with abbreviations for which they suggest an abbreviation chart. Patient suggests use of lay terms to make information clearer. Patient says receiving letter decreases the need to see the GP post-discharge. | | GP and patient agreed that unexplained abbreviations may not be clear to patient and in order to increase patient understanding, acronyms and abbreviations should be spelt out in full and jargon should be accompanied by lay explanations. |
| 11 | S | Letter graded successful as detailed and clear plan. GP did note actions for patient and what the patient told unclear. | Received discharge letter. Patient suggestion that medical terminology could be better explained for patient. Suggestion that verbal explanatory information should accompany letter. | | Patient felt in order to increase their understanding, jargon should be accompanied by lay explanations. |
| 12 | S | Letter graded successful as clear medication information and plan. Generally, GP happy with letter but is not sure how understandable this letter would be to patient. GP feels clinical summary and medication information would be useful to patient and that it is useful for patient to have a copy of the letter. | Patient received letter. Patient found letter information adequate and found medication information particularly useful. Patient felt information and detail in the letter was perhaps excessive and could be shortened and simplified. | | GP and patient in agreement that discharge letter can usefully provide up to date medication information for patient. Patient felt letter contents could be simplified to increase its usefulness to them. |

| Trio case | GP grading | GP comment and interview/focus group findings | Patient interview findings | HP survey findings | Summary of main trio findings |
|---|---|---|---|---|---|
| 13 | S | Letter graded successful as clear medication information and follow up arranged. GP felt it was useful that letter says drugs started and stopped and reasons why. GP felt instructions to patient and follow up very clear. GP feels letter is appropriate and likely to be useful and comprehensible to patient. | Patient showed preference for receiving copies and did receive a copy in this case which they found useful. Patient liked that letter was simple and comprehensive but also brief. Suggestion that letter could be emailed to accelerate process. | | GP and patient in agreement about letter usefulness and comprehensibility to patient. |
| 14 | S | Letter graded successful due to level of detail. GP reported issues with hospitals presuming GPs have access to system to view results when they often do not. Although GP graded letter successful, GP did comment that the letter would benefit from more information regarding the clinical summary and admission details. GP assesses letter as appropriate for patient. | Patient given discharge letter from hospital. Patient happy with this information, they felt it was clear what was wrong, what was going to happen next and medication information. Patient reports no problems with reading or understanding letter. Patient feels letter could have more detail. Patient thinks letter system should be opt out and patients should ideally receive personalised letters. Patient suggests use of lay terms to increase letter usefulness. | | GP and patient in agreement about letter usefulness and comprehensibility to patient as well as level of detail for letter to be useful. Patient suggests use of lay terms to increase usefulness of letter to patient. |
| 15 | US | GP reports issues with the fact that the doctor writing the letter has not seen the patient. GP actions in letter described as ambiguous and inaccuracies noted by GP. The GP felt generally the letter is appropriate for the patient but raises concerns that the vague and unclear parts of the letter may cause patient anxiety. GP suggests how letter could meet needs of both GP and patient through simple interpretations of results and brief summarising of technical information to include breakdown of acronyms. GP felt unexplained acronyms should be avoided for the sake of patient understanding. | Patient not received letter and felt discharge communication process was poor. On letter review, patient was unclear on some of the medical terms in letter. Patient would have preferred to have been given copy of letter by hospital. Patient felt written discharge correspondence to patients should be mandatory. | | GP suggests use of lay terms and simple interpretations to increase usefulness of letter to patient. Patient felt patient correspondence after discharge should be mandatory. GP felt acronyms should be avoided for the sake of understanding and clarity for patient. GP and patient in agreement that discharge communication unsuccessful. |

| Trio case | GP grading | GP comment and interview/focus group findings | Patient interview findings | HP survey findings | Summary of main trio findings |
|---|---|---|---|---|---|
| 16 | S | GP commented that patient not given a copy and they felt that the patient should have and that the letter would have been entirely appropriate for the patient. GP feels letter may have been reassuring for patient. GP comments that sharing letters with patients is the gold standard. Discharge plan simple and letter successful as concise and clear. | Patient reports being copied into recent letters but has found some of the letter contents technical. Despite this patient would prefer to receive copies of the letter sent to the GP rather than a patient personalised letter. Patient feels happy when they receive letters. | | GP preference and patient preference for patients receiving letters. GP and patient disparity about whether or not patient received a copy of their recent discharge letter. |
| 17 | US | Letter graded unsuccessful as limited information regarding medication and investigations. GP found medication information unclear as well as working diagnosis. GP unsure whether or not letter wording would cause patient anxiety due to the diagnosis sounding serious. GP unsure whether letter language comprehensible to patient as many technical medical terms. GP thinks for safety netting, it is useful for the patient to know what the follow up plans are. GP reports information given to patients seems variable. | Patient says they were given discharge letter but with no accompanying verbal information or opportunity to ask questions. Patient reports feeling disappointed with discharge communication. Patient feels letter is not entirely accurate and that there have been ramifications as a result of this. Patient saw serious diagnosis for first time in letter which was slightly worrying. | | GP and patient seem to be in agreement that discharge communication unsuccessful and that it is not ideal for the patient to be finding out about a potentially serious diagnosis for the first time in a letter with no accompanying counselling. |
| 18 | S | GP thinks patients need to know what is happening via a simple letter in lay language. Letter has handwritten pencil annotations which are unclear. Letter graded as successful due to good clinical summary and clear GP actions. GP concerns that receiving this letter may make patient feel anxious. GP raised issues with current prevalence of inaccuracies in discharge letters. | Patient says that they like to receive letters as they like to know what is going on. Patient feels discharge communication is good as long as they get a copy of the discharge letter. | | GP and patient do not seem to be in agreement about patient appropriateness of letter. GP perceives letter may cause patient anxiety when the patient did not report this. |

| Trio case | GP grading | GP comment and interview/focus group findings | Patient interview findings | HP survey findings | Summary of main trio findings |
|---|---|---|---|---|---|
| 19 | S | Letter graded successful as clear diagnosis, summary, medication, diagnosis and plan. Nothing missing from the letter in GP view. To make letter clearer to patient, GP suggests jargon could be broken down and explained. | Patient happy to have received something written down so that they did not have to remember it. Patient mentions jargon not all initially clear but also says terms can be easily looked up on the internet or through other means. Patient likes to receive the same information as their GP. | | GP concerned that patient may not understand letter and that letters such as this may need explaining. Patient happy to have received letter and notes resources such as internet that can be used to look up unknown terms. |
| 20 | US | Letter graded unsuccessful due to lack of medication details. Letter appropriate for patient only if they had knowledge of the information previously. GP thinks it is OK for patients to get copies as long as the letter is clear and meaningful to the patient otherwise the GP will need to spend time explaining letters to patients. | Patient seems somewhat indifferent to receiving letters and is most concerned that a copy is received by the GP. Patient would like to be given choice about receiving letter despite feeling that they often do not need a copy. Patient notes no faults with the letter. | | Patient and GP disagree about quality of letter. |
| 21 | S | GP comments that letter is good quality and sufficiently detailed. GP feels generally letters are appropriate for patients and that it is useful for patients to have record of treatment and medications. | Patient values receiving letters and can understand them and finds them comprehensible. Broadly, patient impressed with letters they have received including the most recent. | | GP and patient in agreement that letter suitable and useful for patient. |
| 22 | US | GP feels letter contains limited detail and no results of investigations or information regarding treatment. Due to lack of information, letter requires GP follow up to clarify details. GP unsure if this letter would be useful to a patient due to the lack of detail. | Patient pleased to have received copy of the discharge letter. Patient found letter very helpful. Patient prefers to receive copy of what is sent to the GP and unsure why anyone would want anything different. Patient cannot see way to improve letter. | | GP and patient disagree on quality of letter. |

| Trio case | GP grading | GP comment and interview/focus group findings | Patient interview findings | HP survey findings | Summary of main trio findings |
|---|---|---|---|---|---|
| 23 | US | Letter grading due to the fact that the letter does not make sense to GP and is generally inadequate. | Patient likes receiving letters and to know what is going on. Patient reported no problems with letter or receiving it. Patient likes to receive a copy the same as what the GP receives. | | GP and patient disagree on letter quality. |
| 24 | S | GP cannot think of case where it would not be appropriate for the patient to have a copy of the letter. GP believes patients receiving letters promotes and encourages autonomy and patient informed-ness and can also be reassuring. GP feels overall letter is clear and succinct. | Patient notes verbal and written information was conflicting. Patient pleased to have received letter and felt it was informative. Patent thinks patients need to know what happened, medication information and follow up plan. Patient feels letter system should be opt out to reduce the risk of patients mistakably not receiving letters. | | GP and patient seem to agree on the benefits of patients receiving letters – that it can inform on condition and what is next. |
| 25 | S | GP expresses concerns with patients comprehending medical terms in discharge letters. GP does add that often patients having letters is useful particularly for GP home visits. GP expounds difficulty writing a letter to meet the needs of two audiences – GP and patient. | Patient reports being given limited information at the time of discharge. Patient notes a few inaccuracies on letter which made them feel uneasy about the rest of the letter and its accuracy, content, and quality. Broadly, patient did not feel the discharge experience was particularly good. | | GP and patient slightly disagree on letter quality – GP grades as successful but patient does not describe communication and discharge experience positively. |
| 26 | S | GP graded letter successful as findings and plan clear. GP feels no new information should be communicated to the patient in the discharge letter. GP thinks that whether or not it is useful for patient to have a copy of the letter depends on the content and quality of letter. GP feels notes letters should never be handwritten as this can be unclear and thinks generally processes need improving to support better communication. | Patient reports being given limited information and no copy of the letter. Patient was left feeling slightly confused about what was going on. Patient would prefer to always receive copies of letter and for this to be the same as what the GP receives. | | GP and patient in agreement that patient receiving letter can be useful. |
