# Supplementary material for: Discharge communication study: a realist evaluation of discharge communication experiences of patients, general practitioners and hospital practitioners, alongside a corresponding discharge letter sample
Source: BMJ Open. 2021 Jul 21;11(7):e045465. doi: 10.1136/bmjopen-2020-045465 (PMC8296817; doi:10.1136/bmjopen-2020-045465)
Supplement: Supplementary data [file bmjopen-2020-045465supp005.pdf]

Quartet meta-matrix with narrative summaries (*US=unsuccessful, S=successful)

Quartet case	GP grading*	GP comment and interview/focus group findings	Patient interview findings	HP survey findings	Main quartet findings
A	US	Letter graded unsuccessful by GP as diagnosis and reason for admission unclear as blank on letter template. GP unclear of cause of patient symptoms and presenting complaint and whether this cause is known to hospital. GP raises possible issues with patient understanding due to presence of jargon and abbreviations. GP thinks avoiding acronyms and use of lay terms in letter may be useful for patient understanding and notes that letter should be provided within context of adequate patient counselling. GP suggests patient information section on letter. GP feels template letters are good as they avoid things being missed. GP likes to know diagnosis, admission and discharge date, consultant details, medication, procedures and results, and patient awareness of diagnosis. GP feels blanks on summaries should not be permitted as unclear.	Patient received copy of letter but did not seem too pleased as they noticed inaccuracies on the letter which made them feel upset/angry. However, patient does find it useful to receive letter so that they can remedy discrepancies. Patient feels someone should go through letter with patients prior to discharge to reduce inaccuracies and ensure patient understanding. Patient prefers to receive direct copy of GP letter. Patient feels letter should have contained name of discharging physician.	HP gave overall letter a quality score of “6/9” with diagnosis information as “2/9” and patient comprehensibility as “2/9”. HP felt patients should have a choice about receiving letters and that they should receive a GP copy. HP notes issues with letters being completed by most junior doctors, some of whom may not be on the corresponding consultant speciality team leading to issues. The HP comments that they tend to dictate letters which allows more information to be inputted as the template can be limiting.	Apparent agreement across all three groups that letter is somewhat unsuccessful. All groups raise issues with letter accuracy and HP notes this is likely due to junior status of completing doctor. GP and HP seem to agree patients should receive letter and patient agrees with this noting that had they not received the letter; they would not have been able to rectify the errors. Patient and GP agree that letter should be provided within the context of patient counselling.
B	US	GP comments that they have no way of knowing whether or not patient received letter. GP feels letter is not patient appropriate and could cause patient to feel anxious due to amount of medical language. GP adds that to improve letter, lay language for patient could be used. GP comments that it is good there are no handwritten sections on letter and that the findings are clear. GP feels patients need to know the procedure and results and follow up. GP comments that it is useful when patients receive letters because it helps them understand the action plan. GP feels that discharge letters need improving in terms of timeliness, factual accuracy, details regarding	Patient been given a copy of letter; it was in an unsealed envelope so they read it. Patient notes that follow up stated on letter has not happened. Patient notes they were lucky to have someone with them in hospital who remembered information as they did not due to effects of anaesthesia. Patient would have preferred interpretative simple summary of results. Patient mentions importance of considerations of the individual	HP gave overall quality score of “5/9” with patient comprehensibility score of “7/9”. HP felt patients should receive choice of receiving letters and that this should be a GP copy. HP notes that they do not always have much time to complete discharge summaries and so must be brief. HP notes completing summaries which are timely but also informative and accurate is very challenging.	GP concerned that patient may not understand letter and that letters such as this may need explaining. Patient happy to have received letter and notes resources such as internet that can be used to look up unknown terms. Lower quality of letter perhaps explained by HP comments regarding the time pressures of completing summaries in their role.

Quartet case	GP grading*	GP comment and interview/focus group findings	Patient interview findings	HP survey findings	Main quartet findings
		what has happened, and plan of action. GP says that GPs are not responsible for chasing results and yet letters request this of them.	and patient choice. Patient notes that unfamiliar terms can easily be searched on internet.	HP notes that they feel their discharge letters are generally adequate but some HPs include only brief details.	
C	S	Successful grading as all information clear and concise including diagnosis and treatment plan. GP feels unexplained acronyms should be minimised for clarity for both GP and patient. GP notes inconsistency of patients receiving letters. GP raises concerns with patient understanding letter due to acronyms, one of which the GP is unfamiliar with, and medical terminology. GP feels that letter should clearly summarise the results in patient-friendly language to make content clearer (e.g. it should be stated that test results were normal for reassurance). GP feels the important items for letters are diagnosis, reason for admission, clinical summary, treatment and results, medication, and follow up and GP actions. GP feels letters are currently very variable in terms of quality. GP thinks patients should only not be given letters in cases of harm. GP comments that the "blank" GP action on letter is confusing and if there is no action this should be explicitly stated for clarity.	Patient has letter and notes that this is useful so if they go abroad they could show the letter to any clinicians looking after them as relevant. Patient notes that different patients may want different levels of information particularly in regard to bad news. Patient reports that they understand letter and are happy with it although they would have preferred to have been given a copy of the letter through the hospital rather than because they took part in the research. Patient suggests letter could be improved by being written in plain English. Patient notes the importance of adequate patient counselling. Patient values knowing next steps.	HP gives letter quality score of "8/9" across all categories to include patient comprehensibility. HP thinks patients should receive a choice of receipt and that the form should be personalised letters. HP rates their letters highly but adds no comments as to why.	GP expresses concerns regarding the patient understanding letter but patient notes that they did understand the contents. However, the GP and patient agree that the letter would be more useful if it was written in plain English with minimal or no acronyms. The HP seems unaware of the acronym issues. The HP feels patients would benefit from personalised letters but patient says they have preference for receiving a copy of what the GP receives. Letter seems to be evaluated as successful across population groups.
D	S	GP thinks patients receive letters variably. GP notes that language in letters is often very medical and so not suitable for the patient without explanation. The GP asserts that letters can be written in a straightforward way for the patient. GP feels patients should receive letters and says this can make patients feel more included in their care. GP feels letter is a bit brief in regard to results and follow up. Good elements of the letter are that tests have been overviewed. The GP feels a summary of the results to include interpretations	Patient says they did not receive a copy of the discharge letter but they would have liked one had it been offered. Patient would have preferred results to have been clearer and letter to make use of lay terms. Patient would like to be given letter every time they attend hospital. Patient suggests letter could be improved by clearer summary of	Letter given "1/9" by HP across quality scores. HP comments that the letter is poor because it was generated by a computer and was not written by themselves.. HP writes that the computer is unable to select the salient information and communicate it and so sometimes they send	HP and GP seem to agree that computerised templates are not particularly helpful. Groups broadly agree about letter quality. All groups agree patients should receive letters.

Quartet case	GP grading*	GP comment and interview/focus group findings	Patient interview findings	HP survey findings	Main quartet findings
		would be useful for the patient and the GP. The GP makes a general comment on the dangers of rapid hospital requests post-discharge.	what happened, medication, treatment, and follow up plans.	a separate letter to the GP with the important information.	
E	S	Letter graded successful as reason for admission and follow up plan were clear as were actions for GP and medication changes. GP favours that GP action in letter not blank but clear that the GP does not need to undertake further actions. GP feels the letter would be appropriate and useful to patient but may be improved by use of lay terms. GP notes patients receive letters inconsistently but they think it is useful for patients to receive copies particularly in regard to medication information. GP notes difficulty of writing letter that is patient friendly whilst meeting technical needs of GP. GP feels information in letter is quite medical and may be confusing/concerning for a patient; GP suggests lay explanations would help. However, GP does note letter would likely be useful for the patient so they are aware of the follow up plan. GP thinks important elements for letters are tests and results, diagnosis, GP action points. GP suggests patients are given abbreviated copies to include diagnosis, medications, and follow ups.	Patient reports that they had not received copy of letter but they would have liked to have done despite that the letter communicated bad news and a serious diagnosis. Patient would prefer copy of what goes to the GP and that this is useful so they can refer back to it so they are not dependent upon remembering information. Patient would like information in the letter relating to what happened and next steps.	HP rates letter “8” in all quality categories including GP information and patient comprehensibility. The HP notes producing summaries on a weekend when they are understaffed is a barrier to producing high quality letters. The HP feels their letter is clear and informative. The HP comments that the [hospital B] discharge templates are superior to the [hospital A] ones as they allow more freedom with inputting information.	The HP reports they always copy patients into letters and yet the patient reported they had not received a copy of the letter. There seems to be agreement across the groups that the letter was successful. GP expresses concern about patient understanding due to medical terms. The patient noted no understanding issues and found the letter useful.
F	US	Letter graded unsuccessful as unclear diagnosis and medication information. GP suggests that letter could be improved by medication information being put at the end of the letter rather than the beginning as this may cloud other important information. GP comments that positive aspects of the letter such as the inclusion of investigations, management plan, and actions for GP. Another letter improvement would be to specify if any blood tests need repeating and if so which ones and when. GP feels patients should receive letters.	Patient reports that they had received a copy of the discharge letter although one page missing when compared with GP copy. Patient found the medication information unclear. Patient also felt the diagnosis information was unclear and that they were given conflicting verbal and written information. The patient comments that they would like to receive a discharge letter every	HP grades letter an “8/9” for overall quality. HP notes restrictive template of summary can be a barrier to providing detail. The HP comments that upon reviewing the diagnosis it is unclear and they should/could have explained the presenting complaint better. The HP comments on the frustration that reports cannot be cut and pasted into the summary and	GP and patient seem to agree that letter requires improvements and that the medication information is unclear. All agree diagnosis information is unclear.

Quartet case	GP grading*	GP comment and interview/focus group findings	Patient interview findings	HP survey findings	Main quartet findings
			time they are discharged from hospital.	that the templates have restricting word counts.	
G	S	Discharge letter successful as it was concise with clear reason for admission, treatment, follow up, information given to patient, investigations and results. GP values that the medication changes in the letter are clear which is useful. GP thinks patients should receive letters but notes issues with jargon. GP feels current quality of discharge letters is variable and many letters have incomplete medication lists and insufficient detail regarding tests carried out and GP actions.	Patient reports being given copy of letter which they were happy with. Patient notes communication difficulties of being transferred between care providers. Patient felt medication information was a bit unclear and that when they were discharged, they still did not know the cause of their condition.	HP gives quality score of “6/9” and patient comprehensibility score of “3/9”. HP thinks patients should receive GP copies but not always. The HP comments that their spelling and grammar let them down but they do feel the management plan and diagnosis in the letter are succinct and informative.	Agreement between GP and patient as letter contained clear follow up and diagnosis but HP rates letter quality lower due their spelling and grammar mistakes.
H	US	Letter graded unsuccessful as no diagnosis and medication list incomplete. GP does note that there is a follow up plan which is helpful but without the diagnosis the letter is not clear enough. GP notes this letter does not contain enough detail. GP feels patients should receive letters but raises issues with unexplained medical terms. GP feels it is useful for patients to have record of medication and treatment. GP feels patient understanding could be improved through adequate patient counselling regarding discharge letter information.	Patient felt unclear of what the problem was when they discharged due to little information received. Patient reports that they did not receive a copy of the discharge letter but they would have liked to have done. Patient suggests that a patient personalised letter may be more valuable but that they would want both letters. Patient mentions use of internet for looking up unknown terms.	HP gives letter a “6/9” for quality and patient comprehensibility but rates diagnosis information a “2/9” as on reflection they feel this is unclear. The HP thinks the follow up information is also poor. HP thinks patients should receive GP copies and always be given a choice of receipt. The HP feels the letter could have been improved by specifying the differential diagnoses in light of the presenting complaint.	Diagnosis information indicated as unsuccessful across all three groups. GP raises issues with patients understanding medical terms. Patient mentioned no issues with letter contents and said that terms can easily be internet searched.
I	S	Successful grading as clear, inclusive of relevant information, and explained what information and advice given to the patient which the GP reports is not always included on summaries but very important. GP suggests issues with patients understanding letters particularly regarding medication changes and feels letters need to be written in plain English and lay language with	Patient reports to be given verbal information only and no letter which they did not find helpful. They would like to receive letters to include more detailed management and recommendations information. Patient wants letter to contain	HP gives scores of “9/9” for all categories except patient comprehensibility which they give “7/9”. HP claims to always copy patients into letters. HP commented that the letter was successful.	GP feels abbreviations need to be avoided in letters as these are not patient friendly. Patient and GP agreed that letter should be written in plain English with explained terms. GP and patient agree that patient actions and

Quartet case	GP grading*	GP comment and interview/focus group findings	Patient interview findings	HP survey findings	Main quartet findings
		minimal or no abbreviations. GP feels patients receiving letters is a good idea but needs to be accompanied by adequate patient counselling and letters should clearly highlight if the patient is required to take any action. GP notes that a successful letter is not a long letter.	specific information about what is wrong, medication, and how condition can be improved. Patient feels receiving verbal & written information is useful.		recommendations need to be explicit and clearer in the letter.
J	US	Unsuccessful grading due to lack of clear findings and follow up plan. GP feels the letter should have included clear details of the discharging physician and also information given to the patient alongside presentation of clinical findings. GP comments that the letter is particularly unclear as it is handwritten and illegible and so they feel uncertain of the exact procedure that the patient has had and the outcome. GP feels that this specific letter would not be helpful to the patient as it contains no information or advice or follow up details. GP also comments that the letter contains too many medical terms which would be hard for the patient to understand. GP notes general usefulness of patients receiving copies but says the letter should accompany counselling. The GP feels letters should always be typed.	Patient reports difficulties remembering the verbal information they were given as no letter. Patient was given a letter for the GP but as it was in a sealed envelope, they did not open it. Patient suggests they should have been given advice for condition and management, details of any follow up and medications, and expectations of recovery. Patient would prefer to receive a direct copy of what is sent to the GP and thinks patients should always be given letters as information can be easily forgotten.	HP gives letter quality score of “2/9” and notes it was actually produced by someone else more junior on their team but the letter has their name on. The HP rated the letter poorly across quality scales but did not provide any details as to how the letter could have been improved.	GP feels nothing in this particular letter would be of use to patient. Patient had trouble remembering the verbal information. Agreement across all three groups that discharge communication poor and unsuccessful. GP notes the illegibility of the letter due to handwritten form. The patient and HP focus on the content brevity. GP and patient agree that patient needs to know advice and follow up plans.
