# Supplementary material for: Discharge communication study: a realist evaluation of discharge communication experiences of patients, general practitioners and hospital practitioners, alongside a corresponding discharge letter sample
Source: BMJ Open. 2021 Jul 21;11(7):e045465. doi: 10.1136/bmjopen-2020-045465 (PMC8296817; doi:10.1136/bmjopen-2020-045465)
Supplement: Author's manuscript [file bmjopen-2020-045465.draft_revisions.pdf]

BMJ Open is committed to open peer review. As part of this commitment we make the peer review history of every article we publish publicly available.

When an article is published we post the peer reviewers' comments and the authors' responses online. We also post the versions of the paper that were used during peer review. These are the versions that the peer review comments apply to.

The versions of the paper that follow are the versions that were submitted during the peer review process. They are not the versions of record or the final published versions. They should not be cited or distributed as the published version of this manuscript.

BMJ Open is an open access journal and the full, final, typeset and author-corrected version of record of the manuscript is available on our site with no access controls, subscription charges or pay-per-view fees (http://bmjopen.bmj.com).

If you have any questions on BMJ Open's open peer review process please email info.bmjopen@bmj.com

BMJ Open

The Discharge Communication Study: a realist evaluation of discharge communication experiences of patients, GPs, and hospital practitioners, alongside a corresponding discharge letter sample

Journal:	*BMJ Open*
Manuscript ID	bmjopen-2020-045465
Article Type:	Original research
Date Submitted by the Author:	01-Oct-2020

Complete List of Authors:	Weetman, Katharine; University of Warwick Warwick Medical School, Unit of Academic Primary Care<br>Dale, Jeremy; University of Warwick Warwick Medical School, Unit of Academic Primary Care<br>Scott, Emma; University of Warwick Warwick Medical School, Unit of Academic Primary Care<br>Schnurr, Stephanie; University of Warwick, Applied Linguistics

Keywords:	Health policy < HEALTH SERVICES ADMINISTRATION & MANAGEMENT, Protocols & guidelines < HEALTH SERVICES ADMINISTRATION & MANAGEMENT, INTERNAL MEDICINE, PRIMARY CARE, QUALITATIVE RESEARCH

SCHOLARONE™
Manuscripts

I, the Submitting Author has the right to grant and does grant on behalf of all authors of the Work (as defined in the below author licence), an exclusive licence and/or a non-exclusive licence for contributions from authors who are: i) UK Crown employees; ii) where BMJ has agreed a CC-BY licence shall apply, and/or iii) in accordance with the terms applicable for US Federal Government officers or employees acting as part of their official duties; on a worldwide, perpetual, irrevocable, royalty-free basis to BMJ Publishing Group Ltd ("BMJ") its licensees and where the relevant Journal is co-owned by BMJ to the co-owners of the Journal, to publish the Work in this journal and any other BMJ products and to exploit all rights, as set out in our licence.

The Submitting Author accepts and understands that any supply made under these terms is made by BMJ to the Submitting Author unless you are acting as an employee on behalf of your employer or a postgraduate student of an affiliated institution which is paying any applicable article publishing charge ("APC") for Open Access articles. Where the Submitting Author wishes to make the Work available on an Open Access basis (and intends to pay the relevant APC), the terms of reuse of such Open Access shall be governed by a Creative Commons licence – details of these licences and which Creative Commons licence will apply to this Work are set out in our licence referred to above.

Other than as permitted in any relevant BMJ Author's Self Archiving Policies, I confirm this Work has not been accepted for publication elsewhere, is not being considered for publication elsewhere and does not duplicate material already published. I confirm all authors consent to publication of this Work and authorise the granting of this licence.

For peer review only

The Discharge Communication Study: a realist evaluation of discharge communication experiences of patients, GPs, and hospital practitioners, alongside a corresponding discharge letter sample

Katharine Weetman[1], Jeremy Dale[2], Emma Scott[3], Stephanie Schnurr[4]

1. Unit of Academic Primary Care, Division of Health Sciences, Warwick Medical School, University of Warwick, Coventry, UK, CV4 7AL, **Early Career Fellow**
2. Unit of Academic Primary Care, Division of Health Sciences, Warwick Medical School, University of Warwick, Coventry, UK, CV4 7AL, **Professor**
3. Unit of Academic Primary Care, Division of Health Sciences, Warwick Medical School, University of Warwick, Coventry, UK, CV4 7AL, **Senior Research Fellow**
4. Applied Linguistics, University of Warwick, Coventry, UK, CV4 7AL, **Associate Professor**

Correspondence to:

Katharine Weetman
Warwick Medical School, University of Warwick, Coventry, UK, CV4 7AL
K.Weetman@warwick.ac.uk

Word count (excluding abstract & tables): 4,157

Key words: Communication; discharge letter; hospital discharge; patient information; discharge summaries, copy letters, doctor patient communication.

Study area: General health/ communication

For peer review only - http://bmjopen.bmj.com/site/about/guidelines.xhtml

TITLE

The Discharge Communication Study: a realist evaluation of discharge communication experiences of patients, GPs, and hospital practitioners, alongside a corresponding discharge letter sample

ABSTRACT (300 words)

Objectives: To develop a programme theory for the intervention of patients receiving discharge letters.

Design: We used a realist evaluation approach and captured multiple perspectives of hospital discharge to refine our previously developed programme theory. GP, patient and hospital clinician views of a single discharge event in which they were all involved were collected using semi-structured interviews and surveys. These were then triangulated to match the corresponding discharge letter. Data was qualitatively synthesised and compared in meta-matrices before interrogation with realist logic of analysis to develop the programme theory that maps out how patients receiving discharge letters works in specific contexts.

Setting: 14 GP practices and four hospital trusts in West Midlands, UK.

Participants: 10 complete matched cases (GP, patient, and hospital practitioner), and a further 26 cases in which a letter was matched with two out of the three participants.

Results: We identified 7 context mechanism outcome configurations not found through literature searching. These related to the broad concepts of: patient preference for receiving letters, patient comprehension of letters, patient-directed letters, patient harm, and clinician views on patients receiving letters. "Patient choice" was important to the success (or not) of the intervention. Other important contexts for positive effects included: letters written in plain English, lay explanations for jargon, written and verbal information provided, no new information in letter, and patient choice acknowledged. Three key findings were: patient understanding is perhaps greater than clinicians perceive, clinician attitudes are a barrier to patients receiving letters, and that, negative outcomes more commonly manifested when patients had not received letters, rather than when they had.

Conclusions: We suggest how patients receiving discharge letters could be improved to enhance patient outcomes. Our programme theory has potential for use in different healthcare contexts and as a framework for policy development relating to patient discharge.

ARTICLE SUMMARY

Strengths and limitations of this study

- First study to compare and contrast matched communication experiences of patients and clinicians in relation to specific discharge letters.
- The inclusion of GP, patient, and hospital practitioner perspectives increased the completeness and relevance of the programme theory.
- The developed programme theory maps out how patients receiving letters works (or not) in specific contexts and may be applicable to multiple healthcare contexts both nationally and internationally.
- The study exclusion criteria restricted the findings such that evidence relating to children, solely to mental health, and those lacking capacity was not considered.
- This study took place with patients treated at four hospital trusts in the West Midlands, UK and the results may not reflect discharge communication experiences of those based in other areas.

For peer review only - http://bmjopen.bmj.com/site/about/guidelines.xhtml

INTRODUCTION

Background

Effective communication during discharge care transitions is essential for patient safety and to reduce negative outcomes [(1)] such as hospital readmissions [(2)]. Despite this, studies [(1, 3-5)] continue to reiterate that processes and content of discharge communication require improvement. Internationally, the practice of patients receiving letters varies but it is generally common for hospital doctors to write directly to GPs or equivalent [(6)]. UK standards and policies [(7-11)] currently outline that patients should receive copies of letters between physicians as a "right" [(11)] and that this is "good practice" [(7)], unless there is risk of harm. Initiatives such as "please write to me" [(8)] by the *Academy of Medical Royal Colleges* have sought to increase practice of patients receiving letters and suggested modifications such as using plain English to increase patient comprehensibility. A recent (2020) review by Rayner *et al.* [(6)] highlighted the value of writing to patients in order to enhance collaborative working and positive outcomes. Despite this, research [(12-14)], both within the UK and internationally, continues to report that patients receive letters inconsistently, the effects of which are unclear [(14, 15)]. Reasons for this inconsistently are little understood but physician attitudes such as concerns about perceived harm may be acting as barrier to policy uptake which has implications for patient experience and safety [(14)]. It is important to understand the extent to which this occurs purposefully, and how this affects patient experience and outcomes.

Our previous realist review [(14)] found conflicts between clinician and patient perspectives in relation to patients receiving discharge letters (e.g. perceived rates of patient understanding). Hence, the current study was designed to shed light on reasons for conflicts through investigating experiences from multiple viewpoints within the same discharge events. The objectives were to undertake an investigation of how patients receiving discharge letters may be improved alongside best practice recommendations and to develop a programme theory for patients receiving letters. As outlined in the work of Pawson [(16-19)], a "programme theory" is useful as it goes beyond consideration of "does it work" and instead seeks to explain *how* an intervention may be theorised to "work" to include within what contexts, for whom, why and to what extent [(16, 20)]. The research questions were:

1. How do the experiences of patients, GPs, and hospital practitioners differ and align within the multi-perspective discharge communication cases?
2. How does patients receiving discharge letters work (or not) and what are the important contexts associated with the desired positive effects?

For peer review only - http://bmjopen.bmj.com/site/about/guidelines.xhtml

This is the final paper in a series forming the Discharge Communication Study [21]; the others are summarised in box 1. Results relating to the GPs and patients are published [22, 23].

Box 1 Summary of discharge communication studies and results

GP study [22]

Methods

- GPs were recruited within the West Midlands (UK) by the local Clinical Research Network.
- Recruited GPs were asked to purposively sample [24] 14-24 recent (<3 weeks) discharge letters in accordance with the inclusion and exclusion criteria (see table 1)
- GPs completed a discharge letter selection template (see supplementary file 1) with their discharge letter grading (successful or unsuccessful) and their comments.
- All GPs were invited to take part in an audio recorded interview or focus group with KW (see supplementary file 2 for interview guide).

Main findings

- 53 GPs selected and commented on discharge letters. 26 of these GPs took part in interviews and focus groups to discuss their views.
- Certain components (e.g. GP actions) were associated with successful gradings.
- Study also found that component (e.g. diagnosis) clarity was important.

Patient study [23]

Methods

- The patients associated with each of the sampled letters were posted an invitation pack by their GP practice. No relationship was established with participants prior to the study.
- The pack invited patients to take part in a 1-1 semi-structured interview at their home or GP surgery with KW (see supplementary file 3 for interview guide).
- All interview/focus group data were audio recorded and transcribed by KW who also took notes. Transcripts were not shown to participants.

Main findings

- 50 patients to whom the sample letters related took part in interviews.
- Study found patients generally wanted to receive letters (approximately 88%).
- Patients also suggested how letter accessibility may be improved (e.g. no acronyms).

Hospital practitioner study

Methods

- The hospital practitioners who wrote the sampled letters were invited to take part in a survey.
- Survey invitation packs were sent by post or distributed by the hospital internally.

Main findings

- 46 hospital practitioners completed surveys.
- Differences between what clinicians felt should be done and what they did in practice e.g. 26 (56.5%) felt patients should always receive letters and 17 (37.0%) did this in practice.
- Hospital practitioners expressed reservations around patients receiving letters (e.g. *"this may cause unnecessary anxiety and distress."*)
- Many responding clinicians (26, 56.5%) unaware of the Department of Health 2003 guidelines on copying letters to patients [7].

For peer review only - http://bmjopen.bmj.com/site/about/guidelines.xhtml

METHODS

Recruitment and data collection

The Discharge Communication Study was an exploratory mixed methods study based in the West Midlands, United Kingdom (UK); the protocol has been published [21]. The intervention under scrutiny 'patients receiving discharge letters' was defined by the team as 'the patient being given or sent any form of written (paper or digital) hospital discharge communication; this could be a direct copy, patient-directed letter, or a combination.' Recruitment took place between August 2017 and September 2018. Box 1 summarises the data collection methods employed across all studies. The study comprised three elements: (1) GP sampling and rating of discharge letters and narrative interviews, (2) semi-structured interviews with patients to whom the letters related, (3) survey of hospital practitioners who wrote the sampled letters. The study design allowed individual discharge letters to be "matched" to different perspectives of those involved within the communicative event. Study specific ID codes allocated to the letters allowed cross-matching with participants to build multiple viewpoint cases termed "quartets" (mapping together four elements if complete, or "trios" if only one perspective missing - see figure 1). Building matched cases allowed direct comparisons between experiences within a single discharge event in order to develop a programme theory for patients receiving discharge letters. The target was to build 30 quartet cases through recruiting 30 GPs, patients and hospital practitioners (HPs) (target n=90).

Table 1 Discharge letter inclusion and exclusion criteria

Inclusion criteria	• NHS adult (18+ years) patients recently discharged (≤3 weeks) from hospital following an episode of inpatient or outpatient care.<br>• Patient registered with the participating GP practice.<br>• Patient treated at and discharged from hospital trusts within Warwickshire, Coventry, Rugby, Herefordshire and Worcestershire.<br>• Cases where written discharge communication has been sent to the patient's GP.
Exclusion criteria	• Age <18 years.<br>• Patients who lack capacity to give informed consent to participate in the study (e.g. Alzheimer's, severe mental illness etc.) or are deemed by the GP to be unsuitable for participation (e.g. end of life).<br>• Patients discharged to providers or units other than their GP (e.g. discharge from hospital to a rehab unit).<br>• Discharge communication from mental health services.<br>• Communication about individuals who are considered unable to participate in an interview or focus group or survey conducted in English.<br>• Letter relates to patient who has expressed a general wish not to participate in research.

Analysis

The study was underpinned by a critical realist framework [25] and a generative view of causation, that is, not just whether an intervention works but in what contexts, how, for

For peer review only - http://bmjopen.bmj.com/site/about/guidelines.xhtml

whom, and why [20]. A realist logic of analysis [16-18, 25] has the potential to account for complexity; discharge communication is complex in many ways such that the letter form (i.e. typed or handwritten) and format (i.e. narrative or templated) as well as the communicative abilities and attitudes of both writers and recipients may vary. This study took a pragmatic approach to realist evaluation [17, 26, 27] in order to apply realist logic to multiple perspective cases within single discharge events. The study drew on realist principles to generate a "programme theory" or theorised explanation of whether or not patients receiving letters "works" (or not) as well as outlining the important relating context [C], mechanism [M], and outcome [O] configurations (CMOCs). The programme theory from our previously conducted realist review [14] was used as the starting theory; this was further developed based on the primary data results and findings. Interrogation and synthesis of evidence for CMOCs used a realist analytic approach [18] to consider the same theory of whether or not "patients receiving letters" works in comparative settings [28]. Thus, analysis was grounded on the assumption that "outcomes" of the intervention may vary according to "context" [28]. All data were inspected for evidence of *"relevance"* [20, 28, 29] to the theory. Manual note-taking on data was then undertaken [14] and judgements were formed as to what any new CMOCs might plausibly be prior to integration into the programme theory.

Data relating to each group was initially analysed separately (see box 1). Findings across groups were then triangulated and a secondary analysis was undertaken using meta-matrices to compare and contrast data. Such triangulation has previously been used within healthcare research [30-32], particularly in relation to healthcare consultations [33-36], to compare multiple perspectives. Multi-perspective case analysis involved re-review of the data for each case; findings from different participants within letter cases were re-read and juxtaposed to highlight agreements and disagreements. Narrative summaries for each case were then developed. Summaries were not intended to be comprehensive but select and include findings of relevance to the research questions. Analysis sought to reconcile previously identified literature disparities on this topic (see our realist review [14]) through highlighting source convergence and divergence in relation to "patients receiving letters".

Patient and public involvement

Around 30 patients were involved in the research design through identifying research priorities [37] by "ranking" potential research questions through completing surveys and taking part in discussions. Four persons with experience as carers from a pre-established panel also provided feedback on the readability and clarity of the patient information materials.

For peer review only - http://bmjopen.bmj.com/site/about/guidelines.xhtml

RESULTS

Recruitment

Figure 2 shows how data collection led to the formation of 26 trio cases (1 GP and HP, 3 patient and HP, 22 patient and GP) and 10 quartet cases (patient, GP, and HP). Table 2 summarises the data characteristics in terms of GP grading, patient gender and age, discharge episode type (inpatient, outpatient…), specialty, and hospital practitioner role. The 10 quartet cases had an even divide of GP graded successful and unsuccessful letters. Four patients reported that they had previously received the discharge letter and six reported that they had not. Letters related to 6 specialties across four hospital trusts.

Table 2 trio and quartet characteristics

Characteristic	Trio cases (n=26)	Quartet cases (n=10)
GP grading	Successful: 18 (69.2%)<br>Unsuccessful: 8 (30.8%)	Successful: 5 (50.0%)<br>Unsuccessful: 5 (50.0%)
No. of GP practices and GPs	14 GP practices, 17 GPs	8 practices 9 GPs
Practice sizes	Small (<5,000 patients): 1 (7.1%)<br>Medium (5-10,000 patients): 8 (57.2%)<br>Large (10,000+ patients): 5 (35.7%)	Small (<5,000 patients): 0 (0.0%)<br>Medium (5-10,000 patients): 4 (50.0%)<br>Large (10,000+ patients): 4 (50.0%)
Patient age	Range: 27-87<br>Median: 67	Range: 59-77<br>Median: 71
Patient gender	Female: 14 (53.8%)<br>Male: 12 (46.2%)	Female: 3 (30.0%)<br>Male: 7 (70.0%)
Admission	Inpatient: 20 (76.9%)<br>Outpatient: 2 (7.7%)<br>Other: 4 (15.4%)	Inpatient: 7 (70.0%)<br>Outpatient: 1 (10.0%)<br>Other: 2 (20.0%)
No. of specialties	13	6
Hospital role of discharging physician	2 different roles<br>Consultant: 20 (76.9%)<br>Core trainee or equivalent: 6 (23.1%)	4 different roles<br>Consultant: 6 (60%)<br>Advanced clinical practitioner: 1 (10%)<br>Junior doctor: 2 (20%)<br>Senior house officer: 1 (10%)

Context mechanism outcome configurations

Narrative summaries for our data are in supplementary file 4 (trios) and 5 (quartets). Following a realist approach, findings were interrogated for theories and CMOCs of "relevance" [20, 28, 29] to patients receiving discharge letters. The following section describes the identified CMOCs and concepts. Sub-heading themes which structured our realist review

For peer review only - http://bmjopen.bmj.com/site/about/guidelines.xhtml

[14] were used and iteratively modified. The 48 CMOCs from the realist review were also systematically interrogated in light of the new evidence; 7 new CMOCs were added. The final table of 55 CMOCs is in supplementary file 6.

Patient preference/choice

Of the 36 cases, 26 patients had received the discharge letter and 10 had not. Patients frequently emphasised positive effects of receiving letters such as increased satisfaction and a sense of involvement [12, 38] [CMOC2]. Patients explained that receiving letters can increase their autonomy and so encourage them to take control and *"ownership"* of their health [CMOC5, CMOC14]. In cases where patients had not received letters (C-E, H-J), patients reported difficulty retaining information and feeling unclear about what happened, their condition and how to manage it. On the other hand, in cases where patients had received letters [context, C](A, B, F, G), patients reported feeling informed and finding the letter useful as a reminder [mechanism, M] of what happened to increase recall [39, 40] [outcome, O] [CMOC15] and decrease the need to memorise information [CMOC50].

Past studies, across a range of settings, report that patient preference for receiving letters is high (79%-97%) [39-46]; this study supports this finding as patients generally indicated preference for discharge letter receipt. Despite this, both GPs and patients noted the inconsistent practice of patients receiving letters. A potential suggested solution was for letters to contain a template "tick box" [C] as to whether or not the patient has been given a letter copy so that it can be audited [O] and increase awareness of the practice [M] [CMOC49]. One new CMOC that emerged was that patients may use the letter [M] as a record [C] for providing evidence for administrative proceedings [O] (e.g. benefits) [CMOC51] or for care within unfamiliar settings (e.g. holidays). Broadly, impacts on patients' experiences were framed as more positive when patients had received discharge letters and more negative when they had not. Crucially, positive outcomes were typically only triggered within key contexts (e.g. letter factually accurate [CMOC15]). Our realist review found patients generally did not object to social habits being included in the letter as long as it had relevance [14]; our findings here caveated this notion in that this information should also be phrased with neutral non-judgemental language [C] to reduce likelihood of upset [M] which could diminish wellbeing [O] [CMOC53]. Crucially, patient preference was not 100% and it is important to consider those who may not wish to receive letters [CMOC40] through acknowledgments of *patient choice* [12, 41-43] [CMOC41]. Moreover, some patients may want to receive letters some of the time but not for every single care episode; patients identified

For peer review only - http://bmjopen.bmj.com/site/about/guidelines.xhtml

this may apply in cases of repeat admissions for the same condition [C] where letters may be repetitive and not helpful [47, 48] [M] and so not requested [O] [CMOC52]. Systems of letter receipt must therefore account for individual case variation.

Patient comprehension

Findings supported previous evidence [41, 45, 48, 49], that patients may understand their letters [M] leading to improved patient knowledge and recall [O] as well as patients feeling empowered to take responsibility for their own health and so carrying out recommendations [CMOC12-15, CMOC54]. However, letters are not always stylistically tailored to patients' needs due to the presence of medical jargon and acronyms. Within some cases (e.g. case 6), the patient and GP agreed that the patient would have benefitted from use of lay terms in the letter to unravel the medical jargon. Case 5 highlighted that unexplained acronyms should be avoided for the sake of both patient and GP comprehensibility. There is a risk that patients receiving letters [C] may increase appointments [O] as patients seek explanations of the letter contents [M] [50]. Nevertheless, in line with past work [46, 51], findings were that this rarely occurs and indeed no study patients reported having made appointments for this purpose [CMOC7, CMOC11]. Furthermore, patients reported that the absence rather than receipt of the letter is what would prompt them to visit the GP [M] and thus increased patient information [C] may reduce rather than increase appointments [O] [CMOC11]. GPs suggested use of a "patient information" section on the letter [C] which provides a letter synopsis in the form of a lay summary to increase understanding [M] and improve patient knowledge and satisfaction [O] [CMOC54]. Patients and GPs agreed that letters should complement rather than substitute verbal information. This is seen in case 17 where the letter communicates a serious diagnosis to the patient and they report being given no other information from the hospital. Hence, letters should only be provided in the context of adequate patient counselling so that the letter is not communicating new information.

Personalised or patient-directed discharge letters

Personalised letters may increase resource use and workload [45, 48, 52] [CMOC25]. There were disagreements as to whether it would be more beneficial for patients to receive a separate personalised letter or the same letter as the GP; some clinicians felt personalised

For peer review only - http://bmjopen.bmj.com/site/about/guidelines.xhtml

letters may improve patient comprehension (e.g. case 1) whereas patients generally preferred to receive the same copy as the GP for transparency and reassurance (e.g. case 3, 22, 23)[CMOC26]. Patients did suggest letter improvements in cases where the clinicians rated the letter successfully (cases B, I); patients felt letters should contain more information regarding how they can improve their condition and recommended patient actions.

Patient harm

Clinicians sometimes had concerns that patients receiving letters may cause harm such as patient anxiety or confusion. However, clinician concern was expressed in several cases where the patients emphasised the benefits of discharge letter receipt (cases B, C, E, G, H). Patients suggested that receiving letters [C] may reduce negative outcomes through reassuring them and reducing or settling anxiety [M] thereby supporting their wellbeing [O] [CMOC39] (case 8). Instances which subverted this trend primarily related to the letter quality (e.g. letter inaccuracies caused stress). One patient found that clear written information in bad news contexts [C] was particularly useful [M] as it allowed them to make an informed end of life plan [O]. Suggestions to reduce risk of harm included ensuring the content is wholly factual and ensuring the patient consents to letter receipt (53) [CMOC41].

Clinician views

Supporting past literature, some clinicians were in favour (51, 54) [CMOC5, CMOC16] of the practice whilst others had reservations (12, 49) [CMOC6, CMOC35]. GPs appeared to be more in favour than hospital practitioners. Nonetheless, some GPs did express issues regarding the inherent need of letters to contain technical information which may not be patient comprehensible. Hospital practitioner concerns included: patient confusion and anxiety (13, 38, 44) [CMOC19], that the patient will not find the letter useful, that letters would need to be oversimplified (12, 55), and that receiving a letter may not be in the best interests of the patient (e.g. mental health cases). Clinician and GP perceived benefits [CMOC5] of patients receiving letters were: increased sense of patient inclusion, improved knowledge (52, 55), and increased transparency (49) [CMOC33]. Our realist review (14) suggested that patient understanding of their letters may be higher than clinicians perceive (45, 51); this study further supports this notion. Comparably to previous literature, concern regarding "patient

For peer review only - http://bmjopen.bmj.com/site/about/guidelines.xhtml

understanding" was common [12, 38, 49, 55] [CMOC6]. However, clinician and patient views were sometimes the antithesis of one another; there were cases where the clinician had concerns [C] regarding patient comprehensibility [M] in cases where the patient reported to have found the letter useful [O][CMOC55] (see cases A-C, E, G-H, J). Patients demonstrated resourcefulness through expounding that unknown terms can be looked up on the internet (case 19) as well as discretion [C] through appreciating that understanding the contents and implications [O] may not necessarily involve comprehending every word [M].

Programme theory

Our findings were used to refine the programme theory, using our realist review [14] as the starting point; changes made to the theory are highlighted in bold (see figure 3). All matched cases were re-read, annotated and interrogated for evidence. Relevant evidence [28, 29] was inspected and concepts drawn on to form the resultant programme theory which shows two main channels: patient copies of letters and patient personalised letters. Contexts for when patients receive letters still contained five key contexts for when this intervention does work but context details were modified. Previously, the theory had four key contexts for when the intervention is theorised not to work; these were updated to include the new context of judgemental language in relation to social behaviour [CMOC53]. Outcomes of patients receiving separate personalised letters were modified; new negative outcomes were overly "basic" content and perceived potential secrecy between clinicians if they are sending and receiving separate letters. "Patient choice" was still a key influencer for likelihood of beneficial outcomes, and contextual influences such as resource provision and directives [CMOC49] were determiners of patients being given a choice of letter receipt [CMOC52].

DISCUSSION

Summary of findings

We undertook a realist evaluation [19, 26, 56, 57] to explore patient, GP and hospital clinician experiences of written discharge communications and hence test and refine the programme theory from our previous realist review [14]. The modified programme theory (figure 3) maps out how patients receiving discharge letters works in specific contexts leading to different positive and negative outcomes. Analysis of the multi-perspective

For peer review only - http://bmjopen.bmj.com/site/about/guidelines.xhtml

discharge events led to the emergence of findings not found in our previous review. Several changes to the initial theory were made to include 10 CMOC modifications and the addition of 7 new CMOCs not found through previous literature searching. No CMOCs were removed. Key contexts for positive outcomes included: letters written in plain English, lay explanations for jargon, written and verbal information provided, no new information in letter, and patient given choice of letter receipt.

While benefits [42, 58] and drawbacks [55, 59] of patients receiving discharge letters have been previously reported, our study adds an understanding of *how* patients receiving letters *works* through outlining the important contexts and associated mechanisms that explain outcome patterns [60, 61]. In addition, the multi-perspective analysis provided possible explanations for previously reported discrepancies identified through our realist review [14]. One example of a discrepancy was that past work highlighted conspicuously inconsistent rates of patient understanding [12, 41, 48, 49, 62, 63]. Data from this study revealed that even in cases where clinicians expressed concerns, patients generally reported to have understood the letter and found it useful. Furthermore, patients often preferred receiving the same letter as the GP rather than a separate letter. Another disparity was in relation to "negative outcomes". A common clinician concern within the study and past literature [13, 38, 44] was that patients receiving letters may cause anxiety and harm. However, literature also reported that patients may find letters useful [12, 45, 48]. Our method highlighted that in several cases where clinicians had concerns, patients who received letters tended to emphasise the positive effects (e.g. increased knowledge). Indeed, patients stressed negative outcomes in contexts where they *had not* rather than *had* received letters. Some patients reported that receiving the letter alleviated anxiety thereby supporting their wellbeing through informing them of their admission, and any next steps, as well as providing reassurance that their GP was updated.

Strengths and weaknesses of the study

We followed RAMESES standards for realist evaluation [27, 64] and completed the COREQ checklist by Tong et al. [65]. To the best of our knowledge, this is the first study to triangulate matched perspectives of patients and clinicians in relation to specific discharge letters. This allowed reconciliation of disparities in the literature and so enabled refinement of the programme theory. Grounding the research in realist theory strengthened the applicability of findings as it facilitated an understanding of not just whether patients should receive letters, but how this practice may "work" as well as in what contexts and why [16, 17].

For peer review only - http://bmjopen.bmj.com/site/about/guidelines.xhtml

As with other realist evaluations [66], the results and findings are intended to have wide applicability to other settings, in this case, settings where adults may receive hospital discharge letters. However, it is important to note the contexts and those groups who were excluded or were under-represented in this study. The exclusion criteria restricted the programme theory such that evidence relating to children, solely to mental health, and those lacking capacity to consent was not considered. Moreover, participation bias may have resulted in the views of ethnic minorities and other marginalised groups being under-represented. The main weakness of the study was the small sample sizes in terms of numbers of patients, sociodemographic diversity of the patients, and range of conditions; for many of the discharge letters it was not possible to form a complete quartet. The study fell short of the target of building 30 quartets; the primary reason for this was under-recruitment of hospital practitioners. The low response rate of hospital practitioners was likely impacted by their lack of available time, our survey recruitment strategy, hospital rotations, and the time lapse between the practitioner writing the letter and receiving the survey invitation. The programme theory would have benefitted from being informed by a larger and more diverse sample of primary evidence. The matched cases relate to a specific geographic area and hence will not have reflected the full range of hospital discharge communication practices that are present nationally. Analysis cannot be considered to be wholly objective due to the influence of researcher identity [67]. Therefore, “reflexivity” was practised throughout the research to reduce but not eradicate bias [67, 68]. Reflexivity was practised through keeping a research diary and regular research team discussion and reflection. Data analysis was also limited by the available evidence which was thin in relation to: dictating letters, the cost of patients receiving letters, doctor-patient relationships, and reasons for variation of practice. Further research is needed to explore these areas as well as the relevance of the programme theory to excluded and under-represented groups, such as those without capacity and children.

Meaning of the study: implications for clinicians and policy makers

The programme theory generated by this study draws on our previous review and primary data, and hence reflects evidence from 16 countries and over 16,000 participants. As such, the theory has both national and international relevance and is likely to be applicable to different healthcare settings. It generally supports policies [7-9, 11] that patients should be offered copies of letters between physicians. Although sending patients’ letters, to include discharge letters, has been recommended practice for almost 20 years [7], uptake

For peer review only - http://bmjopen.bmj.com/site/about/guidelines.xhtml

remains inconsistent [12, 13]. Although national guidelines exist [7-10, 69, 70], each hospital may have its own discharge policy; this means that patients may have different discharge experiences and receive different discharge communications depending on the hospital, discharging physician, and reason for admission, as exemplified in this study. This needs to be addressed with more standardised practices which account for individual preferences and are grounded by *patient choice* with the exception of where there is a risk of "harm", as defined in guideline documents [7]. Patients have a right to receive their letters [11] and should not be denied the opportunity to receive letters based on the perception that their understanding may be low. Although patients may have limited health literacy, they demonstrated resourcefulness and resilience for accessing letter content by looking up unknown terms on the internet and also appreciated that understanding the important features and main directives of a letter does not necessarily involve comprehending every word. Thus, patient understanding is perhaps greater than perceived and the presence of clinical terminology alone is not reason enough to exclude patients from communications. Overall, our study found that negative outcomes more commonly manifested when patients had not received letters, rather than when they had. This included contexts where the clinicians had concern about patient understanding and yet the patient reported to have found the letter of value. It may be inferred that within certain contexts, clinician concerns about patients receiving letters are perhaps unfounded. Thus, clinician attitudes and risk averse behaviour may be acting as a barrier to uptake of this practice.

This research has provided a modified programme theory which demonstrates how policy makers and clinicians may effectively involve patients in their care through provision of written communications. Our theory outlines how both positive and negative outcomes may be produced through this intervention and highlights the importance of contextual considerations [57]. As outlined in previous realist evaluations [61], an advantage of this approach is the relevance of the resultant theory to policy makers as it informs how policy may be adapted to particular purposes and the specific contexts needed to achieve the desired outcomes. An example is the importance of the contextual factor "patient choice of letter receipt" to producing positive outcomes; this is of relevance to policy makers as it explains how best practice of patients receiving letters may be adapted to "work" and how research may be implemented into practice and policy.

CONCLUSION

Sharing information and effective discharge communication with patients should be a priority to improve patient experience and the safety of patient care. This study has yielded

For peer review only - http://bmjopen.bmj.com/site/about/guidelines.xhtml

insights into ways in which practices of patients receiving discharge letters could be improved to enhance patient experience and outcomes. Key findings were: clinicians may underestimate patients' capacity to comprehend discharge letters, patient choice is important for positive outcomes, absence rather than presence of information may be more associated with negative outcomes, and clinician attitudes may be acting as a barrier to patients receiving letters. Our programme theory draws on previous research and experiences of clinicians and patients. The theory has potential for use in different healthcare contexts and as a framework for policy development on patient discharge.

Acknowledgements: The authors would like to express thanks to the GPs, patients, and hospital practitioners who informed the study design as well as those who took part in the study. We would like to thank the West Midlands Clinical Research Network for their support with research recruitment. We would also like to thank the Institute of Advanced Study at the University of Warwick for their support during the write-up of this paper. Finally, we would like to thank Hugh Rayner for providing feedback on an earlier version of the manuscript.

Author contributions: KW, ES, SS and JD substantially contributed to the design and conception of the work and collaborated with KW on data interpretation. KW drafted the manuscript which was then critically revised by JD, ES and SS. All authors approved the final manuscript for publication and agree to be accountable for all aspects of the work.

Funding: This work was supported by the Economic and Social Research Council [grant number ES/J500203/1] and Clinical Commissioning Groups of Coventry & Rugby and South Warwickshire. The funders had no involvement in analysis or preparation of the manuscript.

Competing interests: None declared.

Patient and public involvement: The public and other stakeholder groups were involved in the design and selection of research questions for this research.

Patient consent for publication: Not required.

Ethics approval: Ethics approval was granted by the UK Health Research Authority in July 2017 (Integrated Research Application System ID: 219871, Research Ethics Committee reference: 17/WM/0170, sponsor: University of Warwick).

Data sharing statement: The narrative summaries for data are provided as supplementary files. The full raw datasets for the study are not publicly available due to the sensitive and identifiable nature of the data. Despite names and other identifiers being removed, the in-depth nature of case information may mean that participants could be identified.

References

1.Groene R.O., Orrego C., Sunol R., *et al.* "It's like two worlds apart": an analysis of vulnerable patient handover practices at discharge from hospital. *BMJ Qual Saf*. 2012;21 Suppl 1:i67-75.[Accessed: 10/06/20].

2.Lorenzati B., Quaranta C., Perotto M., *et al.* Discharge communication is an important underestimated problem in emergency department. *Internal & Emergency Medicine*.

For peer review only - http://bmjopen.bmj.com/site/about/guidelines.xhtml

2016;11(1):157-8. Available from: https://dx.doi.org/10.1007/s11739-015-1351-0 [Accessed: 09/07/2020].
3.Rapport F., Hibbert P., Baysari M., *et al.* What do patients really want? An in-depth examination of patient experience in four Australian hospitals. *BMC Health Serv Res*. 2019;19(1):38.[Accessed: 17/07/2020].
4.Flink M., Bergenbrant Glas S., Airosa F., *et al.* Patient-centered handovers between hospital and primary health care: an assessment of medical records. *Int J Med Inform*. 2015;84(5):355-62.[Accessed: 10/06/20].
5.Beaton A., O'Leary K., Thorburn J., *et al.* Improving patient experience and outcomes following serious injury. *N Z Med J*. 2019;132(1494):15-25.[Accessed: 10/06/20].
6.Rayner H., Hickey, M., Logan, I., Mathers, N., Rees, P., Shah, R. Writing outpatient letters to patients. *BMJ*. 2020;368:m24. Available from: https://www.bmj.com/content/bmj/368/bmj.m24.full.pdf [Accessed: 10/07/2020].
7.*Department of Health*. Copying letters to patients: good practice guidelines. 2003. Available from https://webarchive.nationalarchives.gov.uk/20120504030618/http://www.dh.gov.uk/prod_consum_dh/groups/dh_digitalassets/@dh/@en/documents/digitalasset/dh_4086054.pdf
8.The Academy of Medical Royal Colleges. Please, write to me: Writing outpatient clinic letters to patients. 2018. Available from https://www.aomrc.org.uk/reports-guidance/please-write-to-me-writing-outpatient-clinic-letters-to-patients-guidance/
9.National Institute for Health and Care Excellence (NICE). Patient experience in adult NHS services: improving the experience of care for people using adult NHS services 2012. Available from: https://www.nice.org.uk/guidance/cg138.
10.Professional Record Standards Body. Implementation guidance report eDischarge standard. Better records for better care2019. Available from https://theprsb.org/standards/healthandcarerecords/
11.*Department of Health*. The NHS Plan: A Plan for Investment a Plan for Reform. London: HMSO; 2000. Available from https://webarchive.nationalarchives.gov.uk/20121102184216/http://www.dh.gov.uk/en/Publicationsandstatistics/Publications/PublicationsPolicyAndGuidance/DH_4002960
12.Baxter S., Farrell K., Brown C., *et al.* Where have all the copy letters gone? A review of current practice in professional-patient correspondence. *Patient Educ Couns*. 2008;71(2):259-64. Available from: https://dx.doi.org/10.1016/j.pec.2007.12.002 [Accessed: 10/06/20].
13.Boaden R., Harris C. Copying letters to patients—will it happen? *Fam Prac*. 2005;22:141–3. Available from: https://academic.oup.com/fampra/article/22/2/141/522310 [Accessed: 09/07/2020].
14.Weetman K., Wong G., Scott E., *et al.* Improving best practice for patients receiving hospital discharge letters: a realist review. *BMJ Open*. 2019;9(6):e027588. Available from: https://bmjopen.bmj.com/content/bmjopen/9/6/e027588.full.pdf [Accessed: 10/06/20].
15.Harris E., Rob P., Underwood J., *et al.* Should patients still be copied into their letters? A rapid review. *Patient Educ Couns*. 2018;101(12):2065-82. Available from: https://www.sciencedirect.com/science/article/abs/pii/S0738399118303136?via%3Dihub [Accessed: 09/07/2020].
16.Pawson R. Middle range theory and program theory evaluation: From provenance to practice. In: Vaessen J., Leeuw, F.L., editor. *Mind the gap Perspectives on policy evaluation and the social sciences*. New Brunswick, NJ and London: Transaction Publishers; 2010. p. 171-203.
17.Pawson R. *The science of evaluation: a realist manifesto*. London: Sage; 2013.
18.Pawson R. *Evidence-based policy: a realist perspective*. London, UK: Sage; 2006.
19.Pawson R., & Tilley, N. *An introduction to scientific realist evaluation.* 1997 2020/09/02. In: Evaluation for the 21st Century: A Handbook [Internet]. Thousand Oaks,

For peer review only - http://bmjopen.bmj.com/site/about/guidelines.xhtml

California: SAGE Publications, Inc.; [405-18]. https://methods.sagepub.com/book/evaluation-for-the-21st-century.

20. Pawson R., Greenhalgh T., Harvey G., *et al.* Realist review--a new method of systematic review designed for complex policy interventions. *J Health Serv Res Policy*. 2005;10 Suppl 1:21-34. Available from: https://journals.sagepub.com/doi/abs/10.1258/1355819054308530 [Accessed: 09/07/2020].

21. Weetman K., Dale J., Scott E., *et al.* The Discharge Communication Study: research protocol for a mixed methods study to investigate and triangulate discharge communication experiences of patients, GPs, and hospital professionals, alongside a corresponding discharge letter sample. *BMC Health Services Research*. 2019;19(1):825. Available from: https://doi.org/10.1186/s12913-019-4612-1 [Accessed: 10/06/20].

22. Weetman K., Dale J., Spencer R., *et al.* GP perspectives on hospital discharge letters: an interview and focus group study. *BJGP Open*. 2020 https://bjgpopen.org/content/4/2/bjgpopen20X101031. Available from: https://bjgpopen.org/content/4/2/bjgpopen20X101031 [Accessed: 10/06/20].

23. Weetman K., Dale J., Scott E., *et al.* Adult patient perspectives on receiving hospital discharge letters: a corpus analysis of patient interviews. *BMC Health Services Research*. 2020;20(1):537. Available from: https://doi.org/10.1186/s12913-020-05250-1 [Accessed: 24/6/20].

24. Teddlie C., Yu F. Mixed methods sampling: A typology with examples. *Journal of mixed methods research*. 2007;1(1):77-100. Available from: https://journals.sagepub.com/doi/10.1177/1558689806292430 [Accessed: 09/07/2020].

25. Pawson R., Tilley N. *Realistic Evaluation. Evaluation*. London: Sage; 1999.

26. Jagosh J., Bush P.L., Salsberg J., *et al.* A realist evaluation of community-based participatory research: partnership synergy, trust building and related ripple effects. *BMC Public Health*. 2015;15:725. Available from: https://pubmed.ncbi.nlm.nih.gov/26223523/ [Accessed: 21/7/2020].

27. Wong G., Westhorp G., Greenhalgh J., *et al.* Quality and reporting standards, resources, training materials and information for realist evaluation: the RAMESES II project. *NIHR Journals*. 2017;Health Services and Delivery Research(5). Available from: https://pubmed.ncbi.nlm.nih.gov/29072890/ [Accessed: 21/7/2020].

28. Pawson R., Greenhalgh, T., Harvey, G. & Walshe, K. . Realist synthesis: an introduction.' *ESRC Research Methods Programme*. 2004 Available at: https://goo.gl/1Rz2Ry. Available from: Available at: https://goo.gl/1Rz2Ry [Accessed: 04/01/17].

29. Pawson R. Digging for nuggets: how 'bad' research can yield 'good' evidence. *International Journal of Social Research Methodology*. 2006;9(2):127-42. Available from: https://www.tandfonline.com/doi/abs/10.1080/13645570600595314 [Accessed: 09/07/2020].

30. Farmer T., Robinson, K.. Elliott, S. J., Eyles, J. Developing and implementing a triangulation protocol for qualitative health research. *Qual Health Res*. 2006;16(3):377-94. Available from: https://journals.sagepub.com/doi/abs/10.1177/1049732305285708 [Accessed: 09/07/2020].

31. Begley C.M. Using triangulation in nursing research. *Journal of Advanced Nursing*. 1996;24(1):122-8. Available from: https://onlinelibrary.wiley.com/doi/abs/10.1046/j.1365-2648.1996.15217.x [Accessed: 09/07/2020].

32. Dootson S. An in-depth study of triangulation. *Journal of Advanced Nursing*. 1995;22(1):183-7. Available from: https://onlinelibrary.wiley.com/doi/abs/10.1046/j.1365-2648.1995.22010183.x [Accessed: 09/07/2020].

For peer review only - http://bmjopen.bmj.com/site/about/guidelines.xhtml

33.Mendick N., Young, B., Holcombe, C., Salmon, P. The ethics of responsibility and ownership in decision-making about treatment for breast cancer: triangulation of consultation with patient and surgeon perspectives. *Soc Sci Med*. 2010;70(12):1904-11. Available from: https://www.sciencedirect.com/science/article/abs/pii/S027795361000225X?via%3Dihub [Accessed: 09/07/2020].

34.Salmon P., Mendick, N., Young, B. Integrative qualitative communication analysis of consultation and patient and practitioner perspectives: towards a theory of authentic caring in clinical relationships. *Patient Educ Couns*. 2011;82(3):448-54. Available from: https://www.sciencedirect.com/science/article/abs/pii/S0738399110006257?via%3Dihub [Accessed: 09/07/2020].

35.Durif-Bruckert C., Roux, P., Morelle, M., Mignotte, H., Faure, C., Moumjid-Ferdjaoui, N. Shared decision-making in medical encounters regarding breast cancer treatment: the contribution of methodological triangulation. *Eur J Cancer Care (Engl)*. 2015;24(4):461-72. Available from: https://onlinelibrary.wiley.com/doi/abs/10.1111/ecc.12214 [Accessed: 09/07/2020].

36.Bahadori M., Yaghoubi M., Haghgoshyie E., *et al.* Patients' and physicians' perspectives and experiences on the quality of medical consultations: a qualitative evidence. *Int J Evid Based Healthc*. 2019 https://journals.lww.com/ijebh/Abstract/2020/06000/Patients__and_physicians__perspectives_and.9.aspx Available from: https://journals.lww.com/ijebh/Abstract/2020/06000/Patients__and_physicians__perspectives_and.9.aspx [Accessed: 09/07/2020].

37.Cowan K. O., S. James Lind Alliance Guidebook. Southampton: James Lind Alliance; 2013.

38.Tomkins C.S., Braid J.J., Williams H.C. Do dermatology outpatients value a copy of the letter sent to their general practitioner? In what way and at what cost? *Clin Exp Dermatol*. 2004;29(1):81-6. Available from: https://onlinelibrary.wiley.com/doi/abs/10.1111/j.1365-2230.2004.01437.x?sid=nlm%3Apubmed [Accessed: 10/06/20].

39.Antoniou A., Saunders M., Bourner R., *et al.* would you like to see yours? *Bull R Coll Surg Engl*. 2007;89(2):62-4. Available from: https://publishing.rcseng.ac.uk/doi/10.1308/147363507X169936 [Accessed: 09/07/2020].

40.Krishna Y., Damato B.E. Patient attitudes to receiving copies of outpatient clinic letters from the ocular oncologist to the referring ophthalmologist and GP. *Eye (Lond)*. 2005;19(11):1200-4. Available from: https://www.nature.com/articles/6701740 [Accessed: 09/07/2020].

41.Fenton C., Al-Ani A., Trinh A., *et al.* Impact of providing patients with copies of their medical correspondence: a randomised controlled study. *Intern Med J*. 2017;47(1):68-75. Available from: https://onlinelibrary.wiley.com/doi/abs/10.1111/imj.13252 [Accessed: 10/06/20].

42.O'Driscoll B.R., Koch J., Paschalides C. Copying letters to patients: Most patients want copies of letters from outpatient clinics and find them useful. *BMJ*. 2003;327(7412). Available from: http://www.ncbi.nlm.nih.gov/pmc/articles/PMC188516/ [Accessed: 10/07/2020].

43.Rao M., Fogarty P. What did the doctor say? *J Obstet Gynecol*. 2007;27(5):479-80. Available from: https://dx.doi.org/10.1080/01443610701405853 [Accessed: 09/07/2020].

44.Treacy K., Elborn J.S., Rendall J., *et al.* Copying letters to patients with cystic fibrosis (CF): letter content and patient perceptions of benefit. *J Cyst Fibros*. 2008;7(6):511-4. Available from: https://www.cysticfibrosisjournal.com/article/S1569-1993(08)00061-1/fulltext [Accessed: 10/06/20].

For peer review only - http://bmjopen.bmj.com/site/about/guidelines.xhtml

45.Brodie T., Lewis D. A survey of patient views on receiving vascular outpatient letters. *Eur J Vasc Endovasc Surg*. 2010;39(1):5-10. Available from: https://www.ejves.com/article/S1078-5884(09)00500-0/fulltext [Accessed: 09/07/2020].
46.Sharma D., O'Brien S., Hardy K. Copying letters to patients: What patients think - A questionnaire survey. *Clinician in Manage*. 2007;15(2):75-8. Available from: https://www.ncbi.nlm.nih.gov/pmc/articles/PMC2121286/ [Accessed: 09/07/2020].
47.O'Reilly M., Cahill M.R., Perry I.J. Writing to patients: a randomised controlled trial. *Clin Med*. 2006;6(2):178-82. Available from: http://www.clinmed.rcpjournal.org/content/6/2/178.full.pdf [Accessed: 10/06/20].
48.Pinder E., Jefferys S., Loeffler M. Patient Satisfaction: Receiving a copy of the GP letter following fracture or elective orthopaedic clinic. *BMJ Qual Improv Rep*. 2013;2(2). Available from: http://bmjopenquality.bmj.com/content/bmjqir/2/2/u202144.w1085.full.pdf [Accessed: 09/07/2020].
49.Baumann W., Schussler, L., Bertram, M., Benser, J., Kumpers, S., Hermes-Moll, K. Oncologists' letters for breast cancer patients. *Oncol Res Treat*. 2016;39:184-5. Available from: http://dx.doi.org/10.1159/000449050 [Accessed: 10/06/20].
50.Liapi A., Robb P.J., Akthar A. Copying clinic letters to patients: a survey of patient attitudes. *J Laryngol Otol*. 2006;121(6):588-91. Available from: https://www.cambridge.org/core/article/copying-clinic-letters-to-patients-a-survey-of-patient-attitudes/9683993BFBE9720C5C9C13741F285713 [Accessed: 17/07/2020].
51.Brockbank K. Copying patient letters - Making it work. *Clin Gov*. 2005;10(3):231-40. Available from: http://dx.doi.org/10.1108/14777270510627590 [Accessed: 10/06/20].
52.McConnell D., Butow P., Tattersall M. Audiotapes and letters to patients: the practice and views of oncologists, surgeons and general practitioners. *Br J Cancer*. 1999;79:1782-8.[Accessed: 10/06/20].
53.The Newcastle upon Tyne Hospitals NHS Foundation Trust. The Newcastle upon Tyne Hospitals NHS Foundation Trust: Sharing Letters with Patients Policy. 2019. Available from http://www.newcastle-hospitals.org.uk/downloads/policies/Operational/SharingLetterswithPatients201901.pdf
54.Bench S.D., Heelas K., White C., *et al.* Providing critical care patients with a personalised discharge summary: a questionnaire survey and retrospective analysis exploring feasibility and effectiveness. *Intensive & Critical Care Nursing*. 2014;30(2):69-76. Available from: http://www.intensivecriticalcarenursing.com/article/S0964-3397(13)00090-6/fulltext [Accessed: 17/07/2020].
55.O'Reilly M., Cahill M., Perry I.J. Writing to patients: 'putting the patient in the picture'. *Ir Med J*. 2005;98(2):58-60. Available from: https://pubmed.ncbi.nlm.nih.gov/15835515/ [Accessed: 09/07/2020].
56.Martin P., Tannenbaum, C. A realist evaluation of patients' decisions to deprescribe in the EMPOWER trial. *BMJ Open*. 2017;7(4):e015959. Available from: https://bmjopen.bmj.com/content/bmjopen/7/4/e015959.full.pdf [Accessed: 09/09/2020].
57.Crampton P., Mehdizadeh L., Page M., *et al.* Realist evaluation of UK medical education quality assurance. *BMJ Open*. 2019;9(12):e033614. Available from: https://bmjopen.bmj.com/content/bmjopen/9/12/e033614.full.pdf [Accessed: 09/09/2020].
58.Saunders N.C., Georgalas C., Blaney S.P., *et al.* Does receiving a copy of correspondence improve patients' satisfaction with their out-patient consultation? *J Laryngol Otol*. 2003;117(2):126-9. Available from: https://dx.doi.org/10.1258/002221503762624576 [Accessed: 09/07/2020].
59.Hallowell N. Providing letters to patients. Patients find summary letters useful. *BMJ*. 1998;316(7147):1830. Available from: https://www.bmj.com/content/316/7147/1830.3 [Accessed: 09/07/2020].

For peer review only - http://bmjopen.bmj.com/site/about/guidelines.xhtml

60. Kerr H., Price, J., Nicholl, H., O'Halloran, P. Facilitating transition from children's to adult services for young adults with life-limiting conditions (TASYL): Programme theory developed from a mixed methods realist evaluation. *International Journal of Nursing Studies*. 2018;86:125-38. Available from: http://www.sciencedirect.com/science/article/pii/S0020748918301536 [Accessed: 09/09/2020].
61. Willis C.E., Reid S., Elliott C., *et al.* A realist evaluation of a physical activity participation intervention for children and youth with disabilities: what works, for whom, in what circumstances, and how? *BMC Pediatrics*. 2018;18(1):113. Available from: https://doi.org/10.1186/s12887-018-1089-8 [Accessed: 09/09/2020].
62. Lin M.J., Tirosh A.G., Landry A. Examining patient comprehension of emergency department discharge instructions: Who says they understand when they do not? *Internal & Emergency Medicine*. 2015;10(8):993-1002. Available from: https://dx.doi.org/10.1007/s11739-015-1311-8 [Accessed: 09/07/2020].
63. Choudhry A.J., Baghdadi Y.M., Wagie A.E., *et al.* Readability of discharge summaries: with what level of information are we dismissing our patients? *American Journal of Surgery*. 2016;211(3):631-6. Available from: https://dx.doi.org/10.1016/j.amjsurg.2015.12.005 [Accessed: 09/07/2020].
64. Wong G., Westhorp G., Manzano A., *et al.* RAMESES II reporting standards for realist evaluations. *BMC medicine*. 2016;14(1):96-. Available from: https://pubmed.ncbi.nlm.nih.gov/27342217 [Accessed: 21/7/2020].
65. Tong A., Sainsbury P., Craig J. Consolidated criteria for reporting qualitative research (COREQ): a 32-item checklist for interviews and focus groups. *Int J Qual Health Care*. 2007;19(6):349-57. Available from: https://academic.oup.com/intqhc/article/19/6/349/1791966 [Accessed: 09/07/2020].
66. De Sutter M., De Sutter, A., Sundahl, N., Declercq, T., Decat, P. Inter-professional collaboration reduces the burden of caring for patients with mental illnesses in primary healthcare. A realist evaluation study. *European Journal of General Practice*. 2019;25(4):236-42. Available from: https://doi.org/10.1080/13814788.2019.1640209 [Accessed: 09/09/2020].
67. Malterud K. Qualitative research: standards, challenges, and guidelines. *The lancet*. 2001;358(9280):483-8. Available from: https://www.thelancet.com/journals/lancet/article/PIIS0140-6736(01)05627-6/fulltext [Accessed: 09/07/2020].
68. Mays N., Pope C. Qualitative research in health care: Assessing quality in qualitative research. *BMJ*. 2000;320(7226):50. Available from: https://www.ncbi.nlm.nih.gov/pmc/articles/PMC1117321/ [Accessed: 09/07/2020].
69. Royal College of Physicians. Standards for the clinical structure and content of patient records. 2013. Available from https://www.rcplondon.ac.uk/projects/outputs/standards-clinical-structure-and-content-patient-records
70. NHS Digital. The PRSB Standards for the Structure and Content of Health and Care Records. Professional Record Standards Body (PRSB); 2018. Available from https://theprsb.org/standards/healthandcarerecords/

List of figure headings

Figure 1 quartet illustration

Figure 2 recruitment results

Figure 3 resultant programme theory

For peer review only - http://bmjopen.bmj.com/site/about/guidelines.xhtml

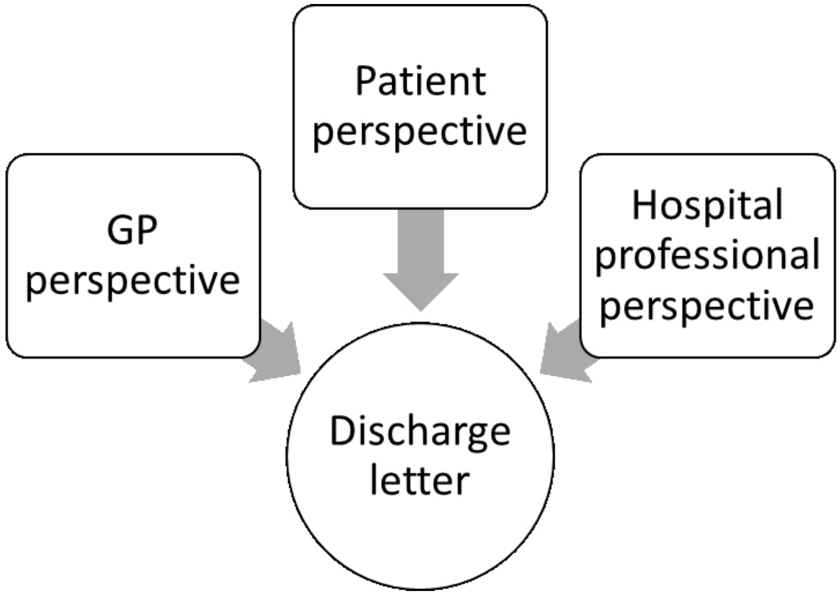

Figure 1 quartet illustration

73x43mm (300 x 300 DPI)

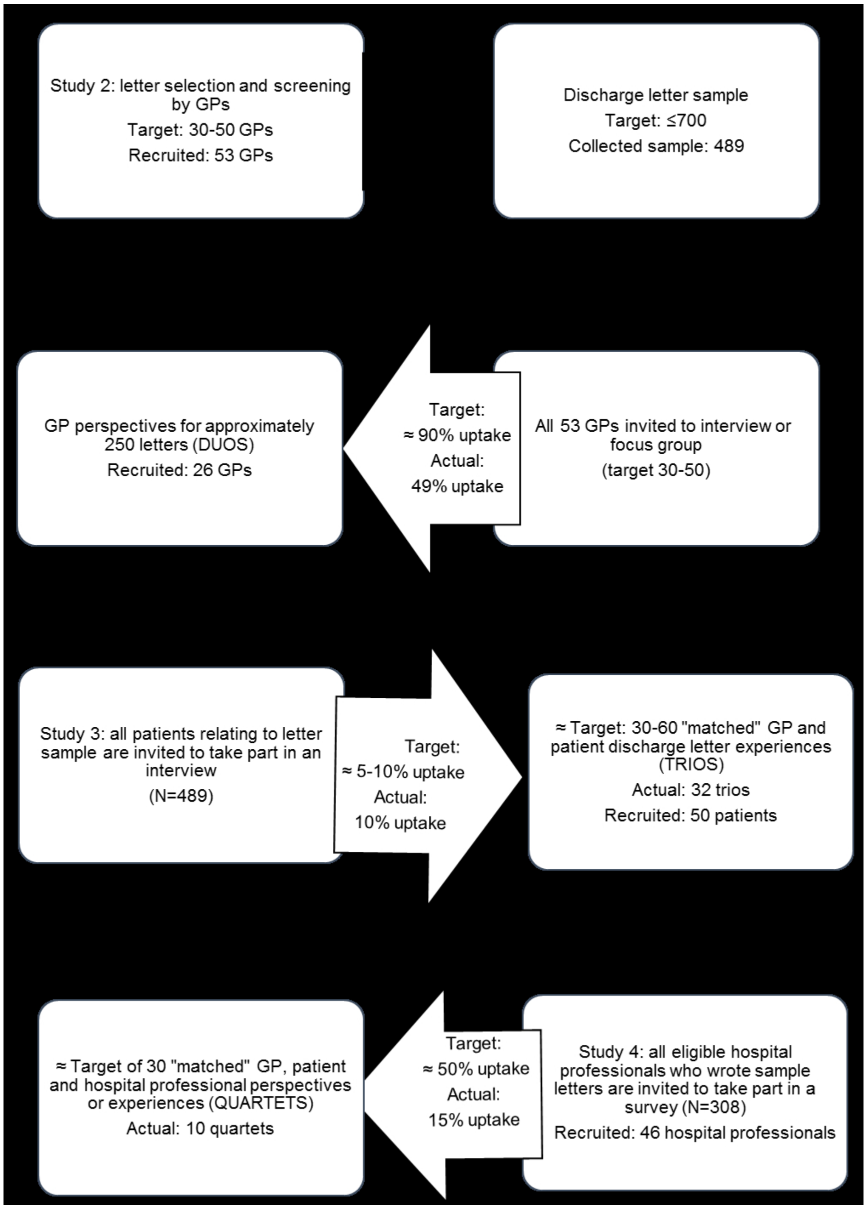

Figure 2 recruitment results

85x119mm (300 x 300 DPI)

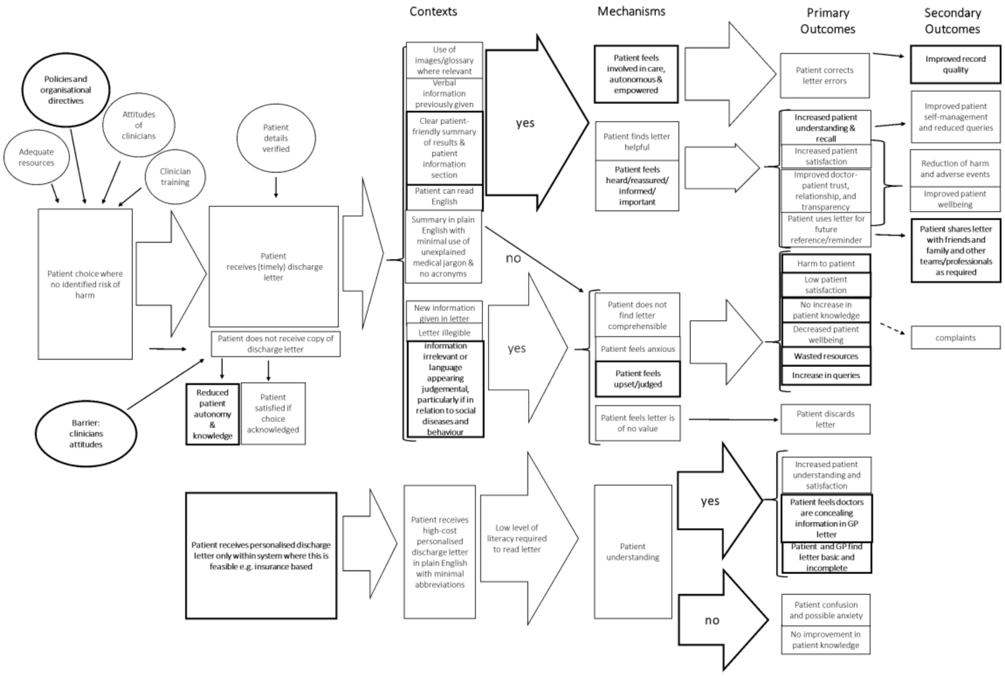

Figure 3 resultant programme theory

234x158mm (300 x 300 DPI)

No. of letters selected	Patient name (to be removed during redaction)	Patient Unique research ID (to be added during redaction)	Categorisation (**Unsuccessful** OR **successful** discharge letter example)	Reason for selection & categorisation (e.g. any key good or bad points about letter)
EXAMPLE (Before redaction)<br><br>*(after redaction)*	*Mr Joe Smith*<br><br>[redacted]	 <br>*P0001*	*Unsuccessful*<br><br>*Unsuccessful*	*Bad points:*<br>*Medication alterations poorly outlined and information given to patient not explained*
1				
2				
3				

More rows to be added as needed…

For peer review only

GP interview and focus group guide

Interviewer opening question:

Please tell me about your experience(s) of patients receiving written discharge communication?

The rest of interview or focus group will continue in a conversational manner discussing GPs views and experiences on patients receiving written discharge communication and how the discharge communication process can be improved.

Possible interviewer prompts:

- What are your experiences of discharge communication as a GP?
- How do you think discharge communication can be improved?
- Please tell me your views on the discharge letters you selected?
- How would you suggest to improve these letters?
- In your opinions, is this letter suitable for a/the patient?
- What are your views on patients receiving letters?
- What do you think are important content items for good quality discharge letters?
- In your view what are the effects and outcomes of poor quality discharge letters?

For peer review only

Patient interview schedule

I: Interviewer (member of the research team) *Action points Q= Question

I: ***Q1: Please tell me about your experiences of receiving any form of written discharge communication? This can be either a direct copy of the letter sent to your GP or a discharge letter specifically addressed to yourself.***

Q2: When you were discharged from hospital on DATE, what information were your given?

****if patient able to be shown letter copy as per protocol, show patient their letter****

Q3: How did you feel about the information you were given?

Q4: What written information would you like to be given or sent when being discharged from hospital and why?

Q5: Would you prefer to receive a direct copy of the letter sent to your GP or a separate letter specifically addressed to yourself?

Q6: Would you like to always be given this letter or would you prefer to choose each time you are discharged?

Q7: How do you think the process of patients receiving written discharge communication can be improved?

Q8: Is there anything else you would like to talk to me about today related to written discharge communication?

Discussion may continue in a relaxed conversational manner and researcher may ask additional questions related to anything else relevant mentioned by the patient.

For peer review only

Trio meta-matrix with narrative summaries (S=successful, US=unsuccessful)

Trio case	GP grading	GP comment and interview/focus group findings	Patient interview findings	HP survey findings	Summary of main trio findings
1	S	Although GP graded letter successful due to clear diagnosis and findings, they did comment that the patient management plan was unclear. GP asserted that they felt patients should receive letters as it informs the patient and is a "safety net" for ensuring follow up plans are actioned.		HP gave letter high quality score of "9/9" and 9s in all other areas including GP care management plan except HP gave letter "4/9" for patient comprehensible. HP concern that patients receiving letters may cause anxiety and distress. HP answered that it would be more appropriate for patients to receive personalised letters.	Although letter graded successful, GP did identify issues. Letter given a top score of "9" by HP. GP and HP appear to disagree about whether patients should receive copies of their discharge letters with HP expressing concern and GP focussing on benefits.
2	S		Patient generally pleased with discharge experience and happy to have received copy of the letter. Patient likes to be informed. Patient suggests some issues with understanding medical terminology and says that they would prefer to receive patient personalised letter. Patient would prefer choice of receiving letter at discharge.	HP gave overall quality score of "7/9" and patient comprehensible score of "9/9". Reports to always copy patients into letters and believes patients should have choice of receiving letters. Answers that patients should receive GP copy of discharge letter.	HP and patient agree about patients receiving letters but appear to disagree over the form that this should take – patient favours personalised correspondence whereas HP favours patients receiving copies of what is sent to the GP.

For peer review only

Trio case	GP grading	GP comment and interview/focus group findings	Patient interview findings	HP survey findings	Summary of main trio findings
3	S		Patient overall seems pleased with communication and adds that they were given written and verbal information but that they asked for a copy of the written information and that this was chased and obtained after discharge. Patient describes follow up information in letter is unclear. Patient happy to receive copy of what GP receives and thinks it is reassuring to view the correspondence between doctors for transparency. Patient would prefer more detailed explanations in letter.	HP gives quality score of “8/9” with patient comprehensibility score of “9/9”. Answers that patients should receive personalised letters and that patients should be given a choice. HP reports that despite hospital policy and their views on patient choice, they have never given a patient a discharge letter copy. HP believes that part of discharge letter should be given to patient and this is what is meant by personalised, not for two summaries to be generated.	HP given letter top score for patient comprehensibility but patient does report some issues and possible improvements which could be made to letter. Patient and HP in agreement over patient choice of receiving letters but disagreement over form. HP says patient personalised but patient says GP copy.
4	S		Patient says they were impressed with information provided; they were given a discharge letter copy. Patient thinks patients should receive letters automatically.	HP gave overall letter score of “9/9” and patient comprehensibility score of “9/9”. Reports to give patients letters most of the time and thinks patients should receive GP copy in opt out style system.	Broad agreement between HP and patient within this trio case.
5	US	Unclear procedure due to acronyms not comprehensible to GP; for this reason, unclear what had been done. GP thinks abbreviations should be written out in full for clarity both for the sake of the patient and themselves.	Patient received letter after long discharge delay in hospital. Patient pleased to have received letter. Patient says they cannot understand all of letter but that they are aware they can ask the GP if they want to understand more.		Patient assumes GP understands all of letter and is a source of information for interpretation when GP does not due to use of uncommon abbreviations in letter.

Trio case	GP grading	GP comment and interview/focus group findings	Patient interview findings	HP survey findings	Summary of main trio findings
6	S	GP considered letter successful as follow up arranged. GP perceives use of acronyms in letter probably not comprehensible to patient. GP thinks use of lay terms in letter may be useful for patient understanding.	Patient thinks letter should ideally be emailed. Patient reports not being given much information and only received letter as relative went to hospital to get a copy after discharge. Patient feels discharge is not always clear and more time needs to be put in to ensure patient understanding. Patient felt letter generally inadequate and unsure of some of medical terms and acronyms in letter, patient states acronyms should not be used and terminology should be explained in lay terms.		GP and patient in agreement that letter format not entirely accessible to patient. Agreement over ways to rectify this issue through avoidance of acronyms and explanations of medical terminology in lay terms.
7	S	Letter graded successful as follow up clear. GP perceives letter written in patient friendly language.	Patient reports no difficulties with letter understanding but does note inaccuracies in letter.		GP and patient appear to agree on patient understanding.
8	US	Letter graded unsuccessful as drug changes and reasons for these unclear.	Patient reports being very pleased to have received copy of discharge letter having been given limited information in regard to previous discharges. Patient felt receiving letter supported their wellbeing. Patient conveys that receiving letter means that they can be actively involved in their own care and thus increase patient autonomy.		Patients receiving letters may support and improve patient wellbeing.

Trio case	GP grading	GP comment and interview/focus group findings	Patient interview findings	HP survey findings	Summary of main trio findings
9	S	GP graded letter successful as it gave full details of investigations and findings and a working diagnosis. Important in GP view for patient to be given plan of action and instructions.	Patient reports not to have been given a copy of the letter. Patient would have preferred to have been given written information to ensure that they do not forget anything.		Patient and GP in agreement that patient did not receive a Letter and both appear to support practice of patients receiving letters.
10	S	Letter graded successful as clear notes. Generally, letter informative and clear. GP raises possible issues with patient understanding due to presence of jargon and abbreviations; GP notes some patients would be fine with not understanding these elements whereas some patients will want to know more and may bring letter to GP with queries. GP says that there is a certain amount of technical information that needs to be passed between doctors but to improve patient understanding the letter should be clear and concise with use of lay language.	Patient given a copy of the letter. Patient reports medication information is very useful and clear but notes some issues with abbreviations for which they suggest an abbreviation chart. Patient suggests use of lay terms to make information clearer. Patient says receiving letter decreases the need to see the GP post-discharge.		GP and patient agreed that unexplained abbreviations may not be clear to patient and in order to increase patient understanding, acronyms and abbreviations should be spelt out in full and jargon should be accompanied by lay explanations.
11	S	Letter graded successful as detailed and clear plan. GP did note actions for patient and what the patient told unclear.	Received discharge letter. Patient suggestion that medical terminology could be better explained for patient. Suggestion that verbal explanatory information should accompany letter.		Patient felt in order to increase their understanding, jargon should be accompanied by lay explanations.

Trio case	GP grading	GP comment and interview/focus group findings	Patient interview findings	HP survey findings	Summary of main trio findings
12	S	Letter graded successful as clear medication information and plan. Generally, GP happy with letter but is not sure how understandable letter would be to patient. GP feels clinical summary and medication information would be useful to patient and that it is useful for patient to have a copy of the letter.	Patient received letter. Patient found letter information adequate and found medication information particularly useful. Patient felt information and detail in the letter was perhaps excessive and could be shortened and simplified.		GP and patient in agreement that discharge letter can usefully provide up to date medication information for patient. Patient felt letter contents could be simplified to increase its usefulness to them.
13	S	Letter graded successful as clear medication information and follow up arranged. GP felt it was useful that letter says drugs started and stopped and reasons why. GP felt instructions to patient and follow up very clear. GP feels letter is appropriate and likely to be useful and comprehensible to patient.	Patient showed preference for receiving copies and did receive a copy in this case which they found useful. Patient liked that letter was simple and comprehensive but also brief. Suggestion that letter could be emailed to accelerate process.		GP and patient in agreement about letter usefulness and comprehensibility to patient.
14	S	Letter graded successful due to level of detail. GP reported issues with hospitals presuming GPs have access to system to view results when they often do not. Although GP graded letter successful, GP did comment that the letter would benefit from more information regarding the clinical summary and admission details. GP assesses letter as appropriate for patient.	Patient given discharge letter from hospital. Patient happy with this information, they felt it was clear what was wrong, what was going to happen next and medication information. Patient reports no problems with reading or understanding letter. Patient feels letter could have more detail. Patient thinks letter system should be opt out and patients should ideally receive personalised letters. Patient suggests use of lay terms to increase usefulness of letter to patient.		GP and patient in agreement about letter usefulness and comprehensibility to patient as well as level of detail for letter to be useful. Patient suggests use of lay terms to increase usefulness of letter to patient.

For peer review only

Trio case	GP grading	GP comment and interview/focus group findings	Patient interview findings	HP survey findings	Summary of main trio findings
15	US	GP reports issues with the fact that the doctor writing the letter has not seen the patient. GP actions in letter described as ambiguous and inaccuracies noted by GP. The GP felt generally the letter is appropriate for the patient but raises concerns that the vague and unclear parts of the letter may cause patient anxiety. GP suggests how letter could meet needs of both GP and patient through simple interpretations of results and brief summarising of technical information to include breakdown of acronyms. GP felt acronyms should be avoided for the sake of patient understanding.	Patient not received letter and felt discharge communication process was poor. Patient unclear on some of the medical terms in letter. Patient would have preferred to have been given copy of letter. Patient felt written discharge correspondence to patients should be mandatory.		GP suggests use of lay terms and simple interpretations to increase usefulness of letter to patient. Patient felt patient correspondence after discharge should be mandatory. GP felt acronyms should be avoided for the sake of understanding and clarity for patient. GP and patient in agreement that discharge communication unsuccessful.
16	S	GP commented that patient not given a copy and they felt that the patient should have and that the letter would have been entirely appropriate for the patient. GP feels letter may have been reassuring for patient. GP comments that sharing letters with patients is the gold standard. Discharge plan simple and letter successful as concise and clear.	Patient reports being copied into recent letters but has found some of the letter contents technical. Despite this patient would prefer to receive copies of the letter sent to the GP rather than a patient personalised letter. Patient feels happy when they receive letters.		GP preference and patient preference for patients receiving letters. GP and patient disparity about whether or not patient received a copy of their recent discharge letter.

For peer review only

Trio case	GP grading	GP comment and interview/focus group findings	Patient interview findings	HP survey findings	Summary of main trio findings
17	US	Letter graded unsuccessful as limited information regarding medication and investigations. GP found medication information unclear as well as working diagnosis. GP unsure whether or not letter wording would cause patient anxiety due to the diagnosis sounding serious. GP unsure whether letter language comprehensible to patient as many technical medical terms. GP thinks for safety netting, it is useful for the patient to know what the follow up plans are. GP reports information given to patients seems variable.	Patient says they were given discharge letter but with no accompanying verbal information or opportunity to ask questions. Patient reports feeling disappointed with discharge communication. Patient feels letter is not entirely accurate and that there have been ramifications as a result of this. Patient saw serious diagnosis for first time in letter which was slightly worrying.		GP and patient seem to be in agreement that discharge communication unsuccessful and that it is not ideal for the patient to be finding out about a potentially serious diagnosis for the first time in a letter with no accompanying counselling.
18	S	GP thinks patients need to know what is happening via a simple letter in lay language. Letter has handwritten pencil annotations which are unclear. Letter graded as successful due to good clinical summary and clear GP actions. GP concerns that receiving this letter may make patient feel anxious. GP raised issues with current prevalence of inaccuracies in discharge letters.	Patient says that they like to receive letters as they like to know what is going on. Patient feels discharge communication is good as long as they get a copy of the discharge letter.		GP and patient do not seem to be in agreement about patient appropriateness of letter. GP perceives letter may cause patient anxiety when the patient did not report this.
19	S	Letter graded successful as clear diagnosis, summary medication, diagnosis and plan. Nothing missing from the letter in GP view. To make letter clearer to patient, GP suggests jargon could be broken down and explained.	Patient happy to have received something written down so that they did not have to remember it. Patient mentions jargon not all initially clear but also says terms can be easily looked up on the internet or through other means. Patient likes to receive the same information as their GP.		GP concerned that patient may not understand letter and that letters such as this may need explaining. Patient happy to have received letter and notes resources such as internet that can be used to look up unknown terms.

For peer review only - http://bmjopen.bmj.com/site/about/guidelines.xhtml

Trio case	GP grading	GP comment and interview/focus group findings	Patient interview findings	HP survey findings	Summary of main trio findings
20	US	Letter graded unsuccessful due to lack of medication details. Letter appropriate for patient only if they had knowledge of the information previously. GP thinks it is OK for patients to get copies as long as the letter is clear and meaningful to the patient otherwise the GP will need to spend time explaining letters to patients.	Patient seems somewhat indifferent to receiving letters and is most concerned that a copy is received by the GP. Patient would like to be given choice about receiving letter despite feeling that they often do not need a copy. Patient notes no faults with the letter.		Patient and GP disagree about quality of letter.
21	S	GP comments that letter is good quality and sufficiently detailed. GP feels generally letters are appropriate for patients and that it is useful for patients to have record of treatment and medications.	Patient values receiving letters and can understand them and finds them comprehensible. Broadly, patient impressed with letters they have received including the most recent.		GP and patient in agreement that letter suitable and useful for patient.
22	US	GP feels letter contains limited detail and no results of investigations or information regarding treatment. Due to lack of information, letter requires GP follow up to clarify details. GP unsure if this letter would be useful to a patient due to the lack of detail.	Patient pleased to have received copy of the discharge letter. Patient found letter very helpful. Patient prefers to receive copy of what is sent to the GP and unsure why anyone would want anything different. Patient cannot see way to improve letter.		GP and patient disagree on letter usefulness to patient and quality of letter.
23	US	Letter grading due to the fact that the letter does not make sense to GP. Although letter graded unsuccessful, GP comments that detail on letter is generally adequate.	Patient likes receiving letters and to know what is going on. Patient reported no problems with letter or receiving it. Patient likes to receive a copy the same as what the GP receives.		GP and patient disagree on letter quality.

For peer review only - http://bmjopen.bmj.com/site/about/guidelines.xhtml

Trio case	GP grading	GP comment and interview/focus group findings	Patient interview findings	HP survey findings	Summary of main trio findings
24	S	GP cannot think of case where it would not be appropriate for the patient to have a copy of the letter. GP believes patients receiving letters promotes and encourages autonomy and patient informed-ness and can also be reassuring. GP feels overall letter is clear and succinct.	Patient notes verbal and written information was conflicting. Patient pleased to have received letter and felt it was informative. Patent thinks patients need to know what happened, medication information and follow up plan. Patient feels letter system should be opt out to reduce the risk of errors of patients not receiving letters.		GP and patient seem to agree on the benefits of patients receiving letters – that it can inform on condition and what is next.
25	S	GP expresses concerns with patients comprehending medical terms in discharge letters. GP does add that often patients having letters is useful particularly for GP home visits. GP expounds difficulty writing a letter to meet the needs of two audiences – GP and patient.	Patient reports being given limited information at the time of discharge. Patient notes a few inaccuracies on letter which made them feel uneasy about the rest of the letter and its accuracy, content and quality. Broadly, patient did not feel the experience was particularly good.		GP and patient slightly disagree on letter quality – GP grades as successful but patient does not describe experience positively.
26	S	GP graded letter successful as findings and plan clear. GP feels no new information should be communicated to the patient in the discharge letter. GP thinks that whether or not it is useful for patient to have a copy of the letter depends on the content and quality of letter. GP feels notes should never be handwritten as this can be unclear and thinks generally processes need improving to support better communication.	Patient reports being given limited information and no copy of the letter. Patient was left feeling slightly confused about what was going on. Patient would prefer to always receive copies of letter and for this to be the same as what the GP receives.		GP and patient in agreement that patient receiving letter can be useful.

For peer review only - http://bmjopen.bmj.com/site/about/guidelines.xhtml

Quartet meta-matrix with narrative summaries (*US=unsuccessful, S=successful)

Quartet case	GP grading*	GP comment and interview/focus group findings	Patient interview findings	HP survey findings	Main quartet findings
A	US	Letter graded unsuccessful by GP as diagnosis and reason for admission unclear as blank on letter template. GP unclear of cause of patient symptoms and presenting complaint and whether this cause is known to hospital. GP raises possible issues with patient understanding due to presence of jargon and abbreviations. GP thinks avoiding acronyms and use of lay terms in letter may be useful for patient understanding and notes that letter should be provided within context of adequate patient counselling. GP suggests patient information section on letter. GP feels template letters are good as they avoid things being missed. GP likes to know diagnosis, admission and discharge date, consultant details, medication, procedures and results, and patient awareness of diagnosis. GP feels blanks on summaries should not be permitted as unclear.	Patient received copy of letter but did not seem too pleased as they noticed inaccuracies on the letter which made them feel upset/angry. However, patient does find it useful to receive letter so that they can remedy discrepancies but they note that the remedying process has been time-consuming. Patient feels someone should go through letter with patients prior to discharge to reduce inaccuracies and ensure patient understanding. Patient prefers to receive direct copy of GP letter. Patient feels letter should have contained name of discharging physician.	HP gave overall letter a quality score of "6/9" with diagnosis information as "2/9" and patient comprehensibility as "2/9". HP felt patients should have a choice about receiving letters and that they should receive a GP copy. HP notes issues with letters being completed by most junior doctors, some of whom may not be on the corresponding consultant speciality team leading to issues. The HP comments that they tend to dictate letters which allows more information to be inputted as the template can be limiting.	Apparent agreement across all three groups that letter is somewhat unsuccessful. All groups raise issues with letter accuracy and HP notes this is likely due to junior status and inexperience of completing doctor. GP and HP seem to agree patients should receive letter and patient agrees with this noting that had they not received the letter; they would not have been able to rectify the errors. Patient and GP agree that letter should be provided within the context of patient counselling.
B	US	GP comments that they have no way of knowing whether or not patient received letter. GP feels letter is not patient appropriate and could cause patient to feel anxious due to amount of medical language and so would not be useful to patient. GP adds that to improve letter, lay language for patient could be used. GP comments that it is good there are no handwritten sections on letter and that the findings are clear. GP feels patients need to know the procedure and results and follow up. GP comments that it is useful when patients receive letters because it helps them understand the action plan. GP feels that discharge letters need improving in terms of timeliness, factual	Patient been given a copy of letter; it was in an unsealed envelope so they read it. Patient notes that follow up stated on letter has not happened. Patient notes they were lucky to have someone with them in hospital who remembered information as they did not due to effects of anaesthesia. Patient would have preferred interpretative simple summary of results. Patient mentions importance of considerations of the individual	HP gave overall quality score of "5/9" with patient comprehensibility score of "7/9". HP felt patients should receive choice of receiving letters and that this should be a GP copy. HP notes that they do not always have very much time to complete discharge summaries and so must keep details brief. HP notes completing summaries which are timely but also informative and accurate is very	GP concerned that patient may not understand letter and that letters such as this may need explaining. Patient happy to have received letter and notes resources such as internet that can be used to look up unknown terms. GP feels letter is not appropriate or useful to patient but patient felt it was. Lower quality of letter perhaps explained by HP comments regarding the time pressures

Quartet case	GP grading*	GP comment and interview/focus group findings	Patient interview findings	HP survey findings	Main quartet findings
		accuracy, details regarding what has happened, and plan of action. GP says that GPs are not responsible for chasing results and yet letters request this of them.	and patient choice. Patient notes that unfamiliar terms can easily be searched on internet.	challenging. HP notes that they feel their discharge letters are generally adequate but some HPs include only brief details.	of completing summaries in their role.
C	S	Successful grading as all information clear and concise including diagnosis and treatment plan. GP feels unexplained acronyms should be minimised for clarity for both GP and patient. GP notes inconsistency of patients receiving letters. GP raises concerns with patient understanding letter due to acronyms, one of which the GP is unfamiliar with, and medical terminology. GP feels that letter should clearly summarise the results in patient-friendly language to make content clearer (e.g. it should be stated that test results were normal for reassurance). GP feels the important items for letters are diagnosis, reason for admission, clinical summary, treatment and results, medication, and follow up and GP actions. GP feels letters are currently very variable in terms of quality. GP thinks patients should only not be given letters in cases of harm (e.g. 3rd party information). GP comments that the "blank" GP action on letter is confusing and if there is no action this should be explicitly stated for clarity.	Patient has letter and notes that this is useful so if they go abroad they could show the letter to any clinicians looking after them as relevant. Patient notes that different patients may want different levels of information particularly in regard to bad news. Patient reports that they understand letter and are happy with it although they would have preferred to have been given a copy of the letter through the hospital rather than because they took part in the research. Patient suggests letter could be improved by being written in plain English. Patient notes the importance of adequate patient counselling. Patient values knowing next steps.	HP gives letter quality score of "8/9" across all categories to include patient comprehensibility. HP thinks patients should receive a choice of receipt and that the form should be personalised letters. HP rates their letters highly but adds no comments as to why.	GP expresses concerns regarding the patient understanding letter but patient notes that they did understand the contents. However, the GP and patient agree that the letter would be more useful if it was written in plain English with minimal or no acronyms. The HP seems unaware of the acronym issues. The HP feels patients would benefit from personalised letters but patient says they have preference for receiving a copy of what the GP receives. Letter seems to be evaluated as successful across population groups.
D	S	GP thinks patients receive letters variably. GP notes that language in letters is often very medical and so not suitable for the patient without explanation. The GP asserts that letters can be written in a straightforward way for the patient. GP feels patients should receive letters and says this can make patients feel more included in their care. GP feels letter is a bit brief as says very little about results and if anything needs to be done in terms of follow up. Good elements of the letter are that	Patient says they did not receive a copy of the discharge letter but they would have liked one had it been offered. Patient would have preferred results to have been clearer and letter to make use of lay terms. Patient would like to be given letter every time they attend hospital. Patient suggests letter could be	Letter given "1/9" by HP across quality scores. HP comments that the letter is poor because it was generated by a computer and was not written by themselves. Criticism that the letter contents are merely a decontextualized list of words. HP writes that the computer is	HP and GP seem to agree that computerised templates are not particularly informative or helpful. Groups broadly agree about poor letter quality. Patient and GP agree information about results is too brief. All groups agree patients should receive letters.

Quartet case	GP grading*	GP comment and interview/focus group findings	Patient interview findings	HP survey findings	Main quartet findings
		tests have been overviewed. The GP feels a summary of the results to include interpretations would be useful for the patient and the GP. The GP makes a general comment on the dangers of rapid hospital requests post-discharge (e.g. asking for a patient review in 3 days).	improved by clearer synopsis of what happened, medication, treatment, and follow up plans.	unable to select the salient information and communicate it and so sometimes they send a separate letter to the GP with the important information.	
E	S	Letter graded successful as reason for admission and follow up plan were clear as were actions for GP and medication changes. GP favours that GP action in letter not blank but clear that the GP does not need to undertake further actions. GP feels the letter would be appropriate and useful to patient but may be improved by use of lay terms. GP notes patients receive letters inconsistently but they think it is useful for patients to receive copies particularly in regard to medication information. GP notes difficulty of writing letter that is patient friendly whilst meeting technical needs of GP. GP feels information in letter is quite medical and may be confusing or concerning for a patient; GP suggests lay explanations to reduce patient anxiety. However, GP does note letter would likely be useful for the patient so they are aware of the follow up plan. GP thinks important elements for letters are tests and results, diagnosis, GP action points. GP suggests patients are given abbreviated copies to include diagnosis, medications, and follow ups.	Patient reports that they had not received copy of letter but they would have liked to have done despite that the letter communicated bad news and a serious diagnosis. Patient would prefer copy of what goes to the GP and that this is useful so they can refer back to it so they are not dependent upon remembering information. Patient notes that the letter relates to them and as such they can relate to the letter. Patient would like information in the letter relating to what happened and next steps.	HP rates letter “8” in all quality categories including GP information and patient comprehensibility. The HP notes producing summarises on a weekend when they are understaffed is a barrier to producing high quality letters. The HP feels their letter is clear and informative. The HP comments that the hospital B discharge templates are superior to the hospital A ones as they allow more freedom with inputting information.	The HP reports they always copy patients into letters and yet the patient reported they had not received a copy of the letter. There seems to be agreement across the groups that the letter was successful. GP expresses concern about patient understanding due to medical terms but the patient noted no understanding issues and found the letter useful.
F	US	Letter graded unsuccessful as unclear diagnosis, medication information regarding why drugs stopped and why. GP suggests that letter could be improved by medication information being put at the end of the letter rather than the beginning as this may cloud other important information. GP comments that positive aspects of the letter are that there are reasons for medication changes alongside investigations, management plan, and	Patient reports that they had received a copy of the discharge letter although one page missing when compared with GP copy. Patient found the medication information unclear. Patient also felt the diagnosis information was unclear and that they were given conflicting verbal and	HP grades letter an “8/9” for overall quality. HP notes restrictive template of summary can be a barrier to providing detail. The HP comments that upon reviewing the diagnosis it is unclear and they should/could have explained the presenting	GP and patient seem to agree that letter requires improvements and that the medication information is unclear. All agree diagnosis information is unclear.

For peer review only

Quartet case	GP grading*	GP comment and interview/focus group findings	Patient interview findings	HP survey findings	Main quartet findings
		actions for GP. Another letter improvement would be to specify if any blood tests need repeating and if so which ones and when. GP feels patients should receive letters.	written information. The patient comments that they would like to receive a discharge letter every time they are discharged from hospital. Patient suggests letters need to include diagnosis, treatment, and management.	complaint better. The HP comments on the frustration that reports cannot be cut and pasted into the summary and that the templates have restricting word counts.	
G	S	Discharge letter successful as it was concise with clear reason for admission, treatment, follow up, information given to patient, investigations and results. GP values that the medication changes in the letter are clear which is useful. GP thinks patients should receive letters but notes issues with jargon. GP feels current quality of discharge letters is variable and many letters have incomplete medication lists and insufficient detail regarding tests carried out and GP actions.	Patient reports being given copy of letter which they were happy with. Patient notes difficulties being transferred from an inpatient to outpatient. Patient felt medication information was a bit unclear and that when they were discharged, they still did not know the cause of their condition.	HP gives quality score of “6/9” and patient comprehensibility score of “3/9”. HP thinks patients should receive GP copies but not always. The HP comments that their spelling and grammar let them down but they do feel the management plan and diagnosis in the letter are succinct and informative.	Agreement between GP and patient as letter contained clear follow up and diagnosis but HP rates letter quality lower due their spelling and grammar mistakes. GP and HP concern about patient understanding; patient noted no issues.
H	US	Letter graded unsuccessful as no diagnosis and medication list incomplete. GP does note that there is a follow up plan which is helpful but without the diagnosis the letter is not clear enough. GP notes this letter does not contain enough detail. GP feels patients should receive letters but raises issues with unexplained medical terms. GP feels it is useful for patients to have record of medication and treatment. GP feels patient understanding could be improved through adequate patient counselling regarding discharge letter information.	Patient felt unclear of what the problem was when they discharged due to little information received. Patient reports that they did not receive a copy of the discharge letter but they would have liked to have done. Patient suggests that a patient personalised letter may be more valuable but that they would want both letters. Patient mentions use of internet for looking up unknown terms.	HP gives letter a “6/9” for quality and patient comprehensibility but rates diagnosis information a “2/9” as on reflection they feel this is unclear as it is missing. The HP thinks the follow up information is also poor. HP thinks patients should receive GP copies and always be given a choice of receipt. The HP feels the letter could have been improved by specifying the differential diagnoses in light of the presenting complaint.	Diagnosis information indicated as unsuccessful across all three groups. GP raises issues with patients understanding medical terms but patient mentioned no issues with letter contents and said that terms can easily be internet searched.
I	S	Successful grading as clear, inclusive of relevant information, and explained what information and	Patient reports to be given verbal information only and no	HP gives scores of “9/9” for all categories except patient	GP feels abbreviations need to be avoided in letters as

For peer review only

Quartet case	GP grading*	GP comment and interview/focus group findings	Patient interview findings	HP survey findings	Main quartet findings
		advice given to the patient which the GP reports is sporadically included on summaries but very important. GP suggests issues with patients understanding letters particularly regarding medication changes and feels letters need to be written in plain English and lay language with minimal or no abbreviations. GP feels patients receiving letters is a good idea but needs to be accompanied by adequate patient counselling and letters should clearly highlight if the patient is required to take any action. GP notes that a successful letter is not a long letter.	letter which they did not find helpful. They would like to receive letters to include more detailed management and recommendations information. Patient wants letter to contain specific information about what is wrong, medication, and how condition can be improved. Patient feels receiving verbal & written information is useful.	comprehensibility which they give "7/9". HP claims to always copy patients into letters. HP commented that the letter was successful.	these are not patient friendly. GP does not rate letters well when information given to patient is unclear. Patient and GP agreed that letter should be written in plain English with explained terms. GP and patient agree that patient actions and recommendations need to be explicit and clearer in the letter.
J	US	Unsuccessful grading due to lack of clear findings and follow up plan. GP feels the letter should have included clear details of the discharging physician and also information given to the patient alongside presentation of clinical findings. GP comments that the letter is particularly unclear as it is handwritten and illegible and so they feel uncertain of the procedure that the patient has had and the outcome. GP feels that this specific letter would not be helpful to the patient as it contains no information or advice or follow up details. GP also comments that the letter contains too many medical terms which would be hard for the patient to understand. GP notes general usefulness of patients receiving copies but says the letter should accompany counselling. The GP feels letters should always be typed.	Patient reports difficulties remembering the verbal information they were given as no letter. Patient was given a letter for the GP but as it was in a sealed envelope, they did not open it. Patient suggests they should have been given advice for condition and management, details of any follow up and medications, and expectations of recovery. Patient would prefer to receive a direct copy of what is sent to the GP and thinks patients should always be given letters as information can be easily forgotten.	HP gives letter quality score of "2/9" and notes it was actually produced by someone else more junior on their team but the letter has their name on. The HP rated the letter poorly across quality scales but did not provide any details as to how the letter could have been improved.	GP feels nothing in this particular letter would be of use to patient. Patient had trouble remembering the verbal information. Agreement across all three groups that discharge communication poor and unsuccessful. GP notes the illegibility of the letter due to handwritten form but the patient and HP do not comment on this but instead focus on the content brevity. GP and patient agree that patient needs to know advice and follow up plans.

For peer review only

Table of Developed CMOCs (context, mechanism, outcomes configurations)

CMOC	Context	Mechanism	Outcome	Effect assessment	Does it "work" or not?
CMOC1	patient not offered letter	patient feels less involved in care	reduced patient autonomy	negative	does not work
CMOC2	patient offered opportunity to receive letter(s)/patient choice respected	patient feels more informed and involved in care	increased patient autonomy and increased involvement of patients in treatment, care and communications	positive	does work
CMOC3	large clear posters displaying patients right to choose and importance of correct contact information	patient realises they should inform hospital of address changes and preferences	lowered risk of confidentiality breach	positive	does work
CMOC4	NHS drive for patient-led care (influence or context)	clinicians increasingly offering patient choice of receiving letter/sharing information with patients	increased patient empowerment	positive	does work
CMOC5	clinician views letters to patients are beneficial e.g. increases transparency, compliance, trust, patient satisfaction, patient understanding and recall	clinician feels patient should be offered letter	potential increase in patient autonomy & satisfaction	positive	does work
CMOC6	Clinicians views letters to patients as not beneficial e.g. letter not comprehensible to patient, medico-legal issues, increased cost and staff workload, patient harm	clinician feels patient should not be offered letter	no patient autonomy	N/A	unclear

For peer review only

CMOC	Context	Mechanism	Outcome	Effect assessment	Does it "work" or not?
	(anxiety, distress, and confusion) and issues around confidentiality				
CMOC7	NHS guidance that all hospital-GP correspondence should be copied to patient as a "right" where appropriate and if patients agree (unless risk of serious harm or legal issues)	clinicians increasingly offering patient choice of receiving letter	increased use of NHS resources to send letters but patient benefits through increased understanding & potential reduction in patient queries (costs balanced)	positive	does work
CMOC8	Data Protection Act 1998 (UK)	Patients may become aware of their right to know what is written & stored about them	Patients informed of their stored electronic information (increased transparency)	positive	does work
CMOC9	doctor copies letters to patient	patient trusts doctor more	improved doctor-patient relationship	positive	does work
CMOC10	patient offered choice of receiving letters	patient chooses to receive letters	Increased administrative staff workload and costs of printing & posting letters	negative	unclear
CMOC11	patient offered choice of receiving letters	patient chooses to receive letters	reduced queries and GP visits and reduced hospital re-admissions (limited evidence)	positive	does work
CMOC12	structured discharge letters written clearly in plain English (pref. 5th grade level) with medical jargon explained with lay terms, no value judgements of patients and minimal abbreviations	patients understand letter	increased patient knowledge	positive	does work

For peer review only

CMOC	Context	Mechanism	Outcome	Effect assessment	Does it "work" or not?
CMOC13	doctors provided training in letter writing & record keeping (contextual influence) leading to doctors write letters of higher quality and more appropriate for patients	patients understand letter	Increased patient knowledge/potential increase in doctor confidence in letter writing	positive	does work
CMOC14	patient preference for letter copies acknowledged and patient offered choice of receiving letter	patients feel able to express their preference	decreased strain on resources & increased patient autonomy & satisfaction	positive	does work
CMOC15	patient provided written & verbal information to include sufficient counselling	patient reflects on written record of information for reference	increased patient knowledge of care plan, recall and acceptance of illness or condition	positive	does work
CMOC16	Human Rights Act (1998) and Race Revelations Act (2000) - clinicians equally offer all patients letter copies regardless of background	clinician feels all patients should be offered letter	increased equality and accessibility of information to patients	positive	does work
CMOC17	Use of pictures/pictographs/cartoons with written information	patients understand letter	Patient benefits from improved understanding e.g. adherence to agreed care plan	positive	does work
CMOC18	verbal information only	patient may not be able to retain information	reduced patient recall	negative	does not work
CMOC19	professionals who are not involved/limited involvement with patient writes letter	professional does not understand patient plan	letter quality reduced/increased risk of harm	negative	does not work
CMOC20	patient hospital visit of sensitive nature and/or patient lacks capacity e.g. psychotic episode, dementia	patient finds letter distressing and/or confusing	harm to patient	negative	does not work

For peer review only

CMOC	Context	Mechanism	Outcome	Effect assessment	Does it "work" or not?
CMOC21	Patient letter written above patient educational level or in a language the patient does not read	patient finds letter difficult to understand	patient is confused with no increased knowledge of care/possible misinterpretation of care instructions	negative	does not work
CMOC22	letter contains inaccurate information	patient identifies inaccuracies	patient notifies hospital/GP of inaccuracies and corrections are made leading to improved record keeping	positive	does work
CMOC23	patient receives discharge letter	patient does not understand entirety of letter	patient sources answers (internet, GP, friend or relative)	positive	does work
CMOC24	Patient specific letter sent to patient	patient finds letter clear	improved patient comprehension	positive	does work
CMOC25	Patient specific letter sent to patient	Clinician produces two letters	increased staff workload and costs	negative	does not work
CMOC26	Patient specific letter sent to patient	Patient identifies information sent to GP and patient is different	medico-legal concerns could be raised over letter discrepancies and any withheld information	negative	does not work
CMOC27	hospital sends patient discharge letter without verifying patient contact details without notifying patient	hospital worker does not identify and correct incorrect information	potential breach of patient confidentiality	negative	does not work
CMOC28	hospital routinely checks patient addresses and sends discharge letters to patient marked confidential using full name	hospital worker identifies and corrects incorrect information	patient receives letter, minimal risk of patient confidentiality breach	positive	does work
CMOC29	patient receives discharge letter	patient may feel they have questions relating to letter	patient contacts health provider with queries (evidence suggests minimal impact and queries)	positive	unclear

For peer review only

CMOC	Context	Mechanism	Outcome	Effect assessment	Does it "work" or not?
CMOC30	discharge letter/summary dictated in front of patient	patient queries any inaccuracies	letter less likely to contain inaccuracies	positive	does work
CMOC31	Hospital gives patient letter to deliver to GP	patient may find they are unable to make delivery or patient does not like being asked to perform this task	GP does not always receive letter. Patient satisfaction low.	negative	does not work
CMOC32	Patient receives letter not written at appropriate level for them	patient does not understand letter	patient feels confused and dissatisfied with discharge care	negative	does not work
CMOC33	Patient has anxiety that doctors talk about things behind their backs	patient who receives letter feels reassured that there is no hidden information	decreased patient anxiety and improved doctor-patient relationship through transparency	positive	does work
CMOC34	patient receives discharge letter	Patient feels they are important to clinician	patient is impressed with letter and feels clinician has an interest	positive	does work
CMOC35	choice about whether letter is sent to patient	clinician feels letters would be a disaster and inappropriate for patient	patient does not receive letter(s)	N/A	unclear
CMOC36	patient receives discharge letter	Patient feels indifferent	no impact on patient	N/A	unclear
CMOC37	patient receives discharge letter with bad news	Patient finds letter initially distressing	letter causes initial distress but final outcome that patient finds letter helpful and aids recall and acceptance of condition	positive	does work
CMOC38	letter sent to patient containing information not discussed with patient or abnormal results	patient feels distressed and anxious reading letter	patient harm/unethical practice	negative	does not work
CMOC39	patient worried about diagnosis and receives letter	patient understanding helped by letter	patient feels less anxious due to being more informed	positive	does work

For peer review only

CMOC	Context	Mechanism	Outcome	Effect assessment	Does it "work" or not?
CMOC40	patient preference for letter copies not acknowledged	Patient dissatisfied to have received letter	decreased patient satisfaction	negative	does not work
CMOC41	patient offered choice of receiving letters (opt out)	patient enabled to decide on letter preference	patient may or may not receive letter depending on their preference in relation to the particular care episode resulting in higher patient satisfaction. Increased rate of patients receiving letters	positive	does work
CMOC42	patient who feels copies of letters are not necessary for themselves	Patient pleased not to be given letter	patient satisfied, secondary outcomes: costs and time saved	positive	does work
CMOC43	patient receives discharge letter where appropriate	patient understands letter	patient finds letter informative and helpful. Patient wellbeing boosted and supported	positive	does work
CMOC44	patient receives discharge letter where appropriate	patient feels involved in care plan	patient ensures follow up plan is followed and books any necessary tests etc.	positive	does work
CMOC45	patient receives discharge letter where appropriate	patient feels letter is important	letter forms permanent record of hospital visit and kept for future reference. Patient may show letter to family and friends.	positive	does work
CMOC46	patient receives discharge letter for breaking good news	patient reminded of discussion	patient feels reassured and has "peace of mind"	positive	does work
CMOC47	patient receives discharge letter where appropriate (patient choice)	patient likes receiving letter	patient satisfaction increased	positive	does work
CMOC48	patient receives copy of discharge letter where appropriate	patient becomes aware of what GP knows	Patient reassured that GP knows about visit	positive	does work

For peer review only

CMOC	Context	Mechanism	Outcome	Effect assessment	Does it "work" or not?
CMOC49	Tickbox/template allows letter copies to patients to be monitored and audit trailed	HP becomes aware of practice of copying patients letters	Increased practice of patients receiving letters. Inconsistencies can be monitored for improving uptake.	positive	Does work
CMOC50	Letter acts as record of consultation and given to patient	Patient reminded of consultation	Patient recall increased and no need for patient to remember all consultation information	positive	Does work
CMOC51	Letter acts as record of consultation and given to patient	Patient prompted to use letter for administrative proceedings without need to contact GP or hospital	Letters can be used as proof of illness for benefit receipt, government support, disability applications and allowances, or time off work.	positive	Does work
CMOC52	Patient episode of care due to repeat or ongoing condition	Patient feels already informed about condition	Patient chooses not to receive letter preserving resources	positive	Does work
CMOC53	Patient receives letter with irrelevant or poorly phrased social disease or behaviour details	Patient feels judged and upset	Patient reflects on episode of care poorly and wellbeing negatively impacted	negative	Does not work
CMOC54	Letter provided to patient with additional patient information section	Patient understands summary	Patient knowledge increased and patient reassured that the important content points have been communicated.	positive	Does work
CMOC55	Clinician concern about patient understanding letter	Patient feels they do understand letter	Clinician concerns potentially unfounded. Patient values receiving letter	positive	Does work

For peer review only

COREQ (COnsolidated criteria for REporting Qualitative research) Checklist

A checklist of items that should be included in reports of qualitative research. You must report the page number in your manuscript where you consider each of the items listed in this checklist. If you have not included this information, either revise your manuscript accordingly before submitting or note N/A.

Topic	Item No.	Guide Questions/Description	Reported on Page No.
**Domain 1: Research team and reflexivity**			
Personal characteristics			
Interviewer/facilitator	1	Which author/s conducted the interview or focus group?	
Credentials	2	What were the researcher's credentials? E.g. PhD, MD	
Occupation	3	What was their occupation at the time of the study?	
Gender	4	Was the researcher male or female?	
Experience and training	5	What experience or training did the researcher have?	
Relationship with participants			
Relationship established	6	Was a relationship established prior to study commencement?	
Participant knowledge of the interviewer	7	What did the participants know about the researcher? e.g. personal goals, reasons for doing the research	
Interviewer characteristics	8	What characteristics were reported about the inter viewer/facilitator? e.g. Bias, assumptions, reasons and interests in the research topic	
**Domain 2: Study design**			
Theoretical framework			
Methodological orientation and Theory	9	What methodological orientation was stated to underpin the study? e.g. grounded theory, discourse analysis, ethnography, phenomenology, content analysis	
Participant selection			
Sampling	10	How were participants selected? e.g. purposive, convenience, consecutive, snowball	
Method of approach	11	How were participants approached? e.g. face-to-face, telephone, mail, email	
Sample size	12	How many participants were in the study?	
Non-participation	13	How many people refused to participate or dropped out? Reasons?	
Setting			
Setting of data collection	14	Where was the data collected? e.g. home, clinic, workplace	
Presence of non-participants	15	Was anyone else present besides the participants and researchers?	
Description of sample	16	What are the important characteristics of the sample? e.g. demographic data, date	
Data collection			
Interview guide	17	Were questions, prompts, guides provided by the authors? Was it pilot tested?	
Repeat interviews	18	Were repeat inter views carried out? If yes, how many?	
Audio/visual recording	19	Did the research use audio or visual recording to collect the data?	
Field notes	20	Were field notes made during and/or after the inter view or focus group?	
Duration	21	What was the duration of the inter views or focus group?	
Data saturation	22	Was data saturation discussed?	
Transcripts returned	23	Were transcripts returned to participants for comment and/or	

For peer review only

Topic	Item No.	Guide Questions/Description	Reported on Page No.
		correction?	
**Domain 3: analysis and findings**			
Data analysis			
Number of data coders	24	How many data coders coded the data?	
Description of the coding tree	25	Did authors provide a description of the coding tree?	
Derivation of themes	26	Were themes identified in advance or derived from the data?	
Software	27	What software, if applicable, was used to manage the data?	
Participant checking	28	Did participants provide feedback on the findings?	
Reporting			
Quotations presented	29	Were participant quotations presented to illustrate the themes/findings? Was each quotation identified? e.g. participant number	
Data and findings consistent	30	Was there consistency between the data presented and the findings?	
Clarity of major themes	31	Were major themes clearly presented in the findings?	
Clarity of minor themes	32	Is there a description of diverse cases or discussion of minor themes?	

Developed from: Tong A, Sainsbury P, Craig J. Consolidated criteria for reporting qualitative research (COREQ): a 32-item checklist for interviews and focus groups. *International Journal for Quality in Health Care*. 2007. Volume 19, Number 6: pp. 349 – 357

Once you have completed this checklist, please save a copy and upload it as part of your submission. DO NOT include this checklist as part of the main manuscript document. It must be uploaded as a separate file.

For peer review only

The Discharge Communication Study: a realist evaluation of discharge communication experiences of patients, General Practitioners, and hospital practitioners, alongside a corresponding discharge letter sample

Journal:	*BMJ Open*
Manuscript ID	bmjopen-2020-045465.R1
Article Type:	Original research
Date Submitted by the Author:	25-Jan-2021
Complete List of Authors:	Weetman, Katharine; University of Warwick Warwick Medical School, Unit of Academic Primary Care<br>Dale, Jeremy; University of Warwick Warwick Medical School, Unit of Academic Primary Care<br>Scott, Emma; University of Warwick Warwick Medical School, Unit of Academic Primary Care<br>Schnurr, Stephanie; University of Warwick, Applied Linguistics
**Primary Subject Heading**:	Health services research
Secondary Subject Heading:	General practice / Family practice, Health policy, Patient-centred medicine, Qualitative research, Communication
Keywords:	Health policy < HEALTH SERVICES ADMINISTRATION & MANAGEMENT, Protocols & guidelines < HEALTH SERVICES ADMINISTRATION & MANAGEMENT, INTERNAL MEDICINE, PRIMARY CARE, QUALITATIVE RESEARCH

SCHOLARONE™
Manuscripts

BMJ

I, the Submitting Author has the right to grant and does grant on behalf of all authors of the Work (as defined in the below author licence), an exclusive licence and/or a non-exclusive licence for contributions from authors who are: i) UK Crown employees; ii) where BMJ has agreed a CC-BY licence shall apply, and/or iii) in accordance with the terms applicable for US Federal Government officers or employees acting as part of their official duties; on a worldwide, perpetual, irrevocable, royalty-free basis to BMJ Publishing Group Ltd ("BMJ") its licensees and where the relevant Journal is co-owned by BMJ to the co-owners of the Journal, to publish the Work in this journal and any other BMJ products and to exploit all rights, as set out in our licence.

The Submitting Author accepts and understands that any supply made under these terms is made by BMJ to the Submitting Author unless you are acting as an employee on behalf of your employer or a postgraduate student of an affiliated institution which is paying any applicable article publishing charge ("APC") for Open Access articles. Where the Submitting Author wishes to make the Work available on an Open Access basis (and intends to pay the relevant APC), the terms of reuse of such Open Access shall be governed by a Creative Commons licence – details of these licences and which Creative Commons licence will apply to this Work are set out in our licence referred to above.

Other than as permitted in any relevant BMJ Author's Self Archiving Policies, I confirm this Work has not been accepted for publication elsewhere, is not being considered for publication elsewhere and does not duplicate material already published. I confirm all authors consent to publication of this Work and authorise the granting of this licence.

For peer review only

The Discharge Communication Study: a realist evaluation of discharge communication experiences of patients, General Practitioners, and hospital practitioners, alongside a corresponding discharge letter sample

Katharine Weetman[1], Jeremy Dale[2], Emma Scott[3], Stephanie Schnurr[4]

1. Unit of Academic Primary Care, Division of Health Sciences, Warwick Medical School, University of Warwick, Coventry, UK, CV4 7AL, **Early Career Fellow**
2. Unit of Academic Primary Care, Division of Health Sciences, Warwick Medical School, University of Warwick, Coventry, UK, CV4 7AL, **Professor**
3. Unit of Academic Primary Care, Division of Health Sciences, Warwick Medical School, University of Warwick, Coventry, UK, CV4 7AL, **Senior Research Fellow**
4. Applied Linguistics, University of Warwick, Coventry, UK, CV4 7AL, **Associate Professor**

Correspondence to:

Katharine Weetman
Warwick Medical School, University of Warwick, Coventry, UK, CV4 7AL
K.Weetman@warwick.ac.uk

Word count (excluding abstract & tables): 4,641

Key words: Communication; discharge letter; hospital discharge; patient information; discharge summaries, copy letters, doctor patient communication.

Study area: General health/ communication

For peer review only - http://bmjopen.bmj.com/site/about/guidelines.xhtml

TITLE

The Discharge Communication Study: a realist evaluation of discharge communication experiences of patients, General Practitioners, and hospital practitioners, alongside a corresponding discharge letter sample

ABSTRACT (300 words)

Objectives: To develop a programme theory for the intervention of patients receiving discharge letters.

Design: We used a realist evaluation approach and captured multiple perspectives of hospital discharge to refine our previously developed programme theory. General Practitioner (GP), patient and hospital clinician views of a single discharge event in which they were all involved were collected using semi-structured interviews and surveys. These were then triangulated to match the corresponding discharge letter. Data were qualitatively synthesised and compared in meta-matrices before interrogation with realist logic of analysis to develop the programme theory that maps out how patients receiving discharge letters works in specific contexts.

Setting: 14 GP practices and four hospital trusts in West Midlands, UK.

Participants: 10 complete matched cases (GP, patient, and hospital practitioner), and a further 26 cases in which a letter was matched with two out of the three participants.

Results: We identified 7 context mechanism outcome configurations not found through literature searching. These related to the broad concepts of: patient preference for receiving letters, patient comprehension of letters, patient-directed letters, patient harm, and clinician views on patients receiving letters. “Patient choice” was important to the success (or not) of the intervention. Other important contexts for positive effects included: letters written in plain English, lay explanations for jargon, verbal information also provided, no new information in letter, and patient choice acknowledged. Three key findings were: patient understanding is perhaps greater than clinicians perceive, clinician attitudes are a barrier to patients receiving letters, and that, negative outcomes more commonly manifested when patients had not received letters, rather than when they had.

Conclusions: We suggest how patients receiving discharge letters could be improved to enhance patient outcomes. Our programme theory has potential for use in different healthcare contexts and as a framework for policy development relating to patient discharge.

ARTICLE SUMMARY

Strengths and limitations of this study

- First study to compare and contrast matched views of patients, General Practitioners and hospital clinicians in relation to specific discharge letters.
- Realist theory facilitated understanding of not just whether patients should receive letters, but how this practice may “work” in different contexts and why.
- The qualitative methods enabled detailed gathering of the experiences, viewpoints, and attitudes of participants.
- The secondary analysis was limited by weaknesses in the primary dataset, including the sociodemographic diversity of the patients, range of conditions, and limited numbers of cases in which hospital clinician perspectives could be matched to those of GPs and patients.
- Evidence relating to children, mental health admissions, and those lacking capacity was not considered.

For peer review only - http://bmjopen.bmj.com/site/about/guidelines.xhtml

INTRODUCTION

Background

Effective communication during discharge care transitions is essential for patient safety and to reduce negative outcomes [(1)] such as hospital readmissions [(2)]. Despite this, studies [(3-5)] continue to reiterate that processes and content of discharge communication require improvement. Internationally, the practice of patients receiving letters varies but it is generally common for hospital doctors to write directly to General Practitioners (GPs) or equivalent [(6)]. UK standards and policies [(7-11)] currently outline that patients should receive copies of letters between physicians as a "right" [(11)] and that this is "good practice" [(7)], unless there is risk of harm. Initiatives such as "please write to me" [(8)] by the *Academy of Medical Royal Colleges* have sought to increase practice of patients receiving letters and suggested modifications such as using plain English to increase patient comprehensibility. A recent (2020) review by Rayner *et al.* [(6)] highlighted the value of writing to patients in order to enhance collaborative working and positive outcomes. Despite this, research [(12-14)], both within the UK and internationally, continues to report that patients receive letters inconsistently, the effects of which are unclear [(14, 15)]. Reasons for this inconsistency are little understood but physician attitudes such as concerns about perceived harm may be acting as barrier to policy uptake which has implications for patient experience and safety [(14)]. It is important to understand the extent to which this occurs purposefully, and how this affects patient experience and outcomes.

Our previous realist review [(14)] found conflicts between clinician and patient perspectives in relation to patients receiving discharge letters (e.g. perceived rates of patient understanding). Hence, the current study was designed to shed light on reasons for conflicts through investigating experiences from multiple viewpoints within the same discharge events. The objectives were to undertake an investigation of how patients receiving discharge letters may be improved alongside best practice recommendations and to develop a programme theory for patients receiving letters. As outlined in the work of Pawson [(16-19)], a "programme theory" is useful as it goes beyond consideration of "does it work" and instead seeks to explain *how* an intervention may be theorised to "work" to include within what contexts, for whom, why and to what extent [(16, 20)]. The research questions were:

1. How do the experiences of patients, GPs, and hospital practitioners differ and align within the multi-perspective discharge communication cases?
2. How does patients receiving discharge letters work (or not) and what are the important contexts associated with the desired positive effects?

For peer review only - http://bmjopen.bmj.com/site/about/guidelines.xhtml

This is the final paper in a series forming the Discharge Communication Study [21]; the others are summarised in box 1. Results relating to the GPs and patients are published [22, 23].

Box 1 Summary of discharge communication studies and results

GP study [22]

Methods

- 53 GPs were recruited from 18 practices within the West Midlands (UK) through the local Clinical Research Network and Warwick Medical School links with practices.
- They were asked to purposively sample [24] 14-24 recent (<3 weeks) discharge letters in accordance with the inclusion and exclusion criteria (see table 1)
- Each GP completed a discharge letter selection template (see supplementary file 1) with their discharge letter grading (successful or unsuccessful) and their comments.
- A subgroup of 26 GPs took part in an audio recorded interview or focus group; these took place face to face at GP practices and over the telephone (see supplementary file 2 for interview guide).

Main findings

- Key components within discharge letters (e.g. GP actions) associated with successful gradings.
- The importance of clarity and comprehensibility.

Patient study [23]

Methods

- The patients associated with each of the letters sampled by GPs were invited to take part in a 1-1 semi-structured interview at their home or GP surgery (see supplementary file 3 for interview guide).
- No relationship was established with participants prior to the study.
- All interview/focus group data were audio recorded and transcribed by KW who also took notes. Transcripts were not shown to participants.

Main findings

- 50 patients to whom the sample letters related took part in interviews.
- They generally wanted to receive copies of their discharge communication letter.
- Patients also suggested how letter comprehensibility may be improved (e.g. no acronyms).

Hospital practitioner study

Methods

- The hospital practitioners who wrote the letters sampled by GPs were invited to take part in a survey.

Main findings

- 46 hospital practitioners completed surveys.
- There were differences between what clinicians felt should be done and what occurred in practice e.g. 26 (56.5%) felt patients should always receive letters and 17 (37.0%) did this in practice.
- Some hospital practitioners expressed reservations around patients receiving letters.
- Many were unaware of the Department of Health guidelines on copying letters to patients [7].

For peer review only - http://bmjopen.bmj.com/site/about/guidelines.xhtml

METHODS

Study design

This study was a secondary analysis of a subset of data from the Discharge Communication Study, an exploratory mixed methods study based in the West Midlands, United Kingdom (UK) [21]; box 1 gives a brief summary of papers linked to the Discharge Communication Study. The intervention under scrutiny ‘patients receiving discharge letters’ was defined by the team as ‘the patient being given or sent any form of written (paper or digital) hospital discharge communication; this could be a direct copy, patient-directed letter, or a combination.’ Broadly, the data comprised three elements: (1) GP sampling and rating of discharge letters (“successful” or “unsuccessful”) and narrative interviews, (2) semi-structured interviews with patients to whom the letters related, (3) survey of hospital practitioners who wrote the sampled letters.

Settings

The setting for the study is outlined in the published study protocol [21]. It involved four hospital trusts and a diverse range of 18 GP practices in the West Midlands.

Recruitment and data collection

Recruitment and data collection took place, as detailed in previous publications [21-23] between August 2017 and September 2018. In brief, GPs were asked to screen (see table 1 for screening criteria) and select a sample of recently received discharge letters according to what they considered to be “successful” or “unsuccessful” letter exemplars; for each letter, GPs were asked to complete the selection proforma (supplementary file 1) and rate the letters “successful” or “unsuccessful”. There were no set criteria for letter ratings as the selection was based on each participating GP’s interpretation of what makes a successful or unsuccessful discharge letter. This purposive [24] letter sampling approach was intended to increase sample diversity and address the research questions within dichotomous contexts. All GPs involved in letter sampling were then invited to take part in a “narrative” [25] interview or focus group with KW (see supplementary file 2 for interview guide). All patients associated with the sampled discharge letters were sent an invitation pack by their GP practice; this invited them to take part in an audio recorded semi-structured interview with KW (see supplementary file 3 for interview guide). Finally, the hospital professionals who wrote or

For peer review only - http://bmjopen.bmj.com/site/about/guidelines.xhtml

signed the sampled discharge letters were sent an invitation pack by the research team; this invited them to take part in a survey on their evaluation of the discharge letter they wrote, their current practices, and their views about how discharge communication processes may be improved. Packs were sent by post and email as well as being internally distributed by hospital sites.

For this study, we re-interpreted data collected across all of the other studies. This involved a secondary analysis of a subset of the data which was drawn from sampled discharge letters that could be "matched" to at least two other dataset perspectives. Study specific ID codes allocated to the letters allowed cross-matching with participants to build multiple viewpoint cases termed "quartets" (mapping together four elements if complete, or "trios" if only one perspective missing - see figure 1).

The target was to build 30 quartet cases through recruiting at least 30 GPs, patients and hospital practitioners (HPs) (target n=90). Trio and quartet participants were not separately recruited from other studies within the project; instead, cases were built through the participant recruitment and data collection across all studies for the discharge communication project (see figure 2). Once participant data across studies were matched into trio and quartet cases, findings and data were subjected to a secondary level data analysis using a realist approach described below. This allowed highlighting of data convergence and divergence as well as the emergence of new findings which only became apparent through juxtaposition.

Table 1 Discharge letter inclusion and exclusion criteria

Inclusion criteria	• NHS adult (18+ years) patients recently discharged (≤3 weeks) from hospital following an episode of inpatient or outpatient care.<br>• Patient registered with the participating GP practice.<br>• Patient treated at and discharged from hospital trusts within Warwickshire, Coventry, Rugby, Herefordshire and Worcestershire.<br>• Cases where written discharge communication has been sent to the patient's GP.
Exclusion criteria	• Age <18 years.<br>• Patients who lack capacity to give informed consent to participate in the study (e.g. Alzheimer's, severe mental illness etc.) or are deemed by the GP to be unsuitable for participation (e.g. end of life).<br>• Patients discharged to providers or units other than their GP (e.g. discharge from hospital to a rehab unit).<br>• Discharge communication from mental health services.<br>• Communication about individuals who are considered unable to participate in an interview or focus group or survey conducted in English.<br>• Letter relates to patient who has expressed a general wish not to participate in research.

For peer review only - http://bmjopen.bmj.com/site/about/guidelines.xhtml

Analysis

The study was underpinned by a critical realist framework [26] and a generative view of causation, that is, not just whether an intervention works but in what contexts, how, for whom, and why [20]. A realist logic of analysis [16-18, 26] has the potential to account for complexity; discharge communication is complex in many ways such that the letter form (i.e. typed or handwritten) and format (i.e. narrative or templated) as well as the communicative abilities and attitudes of both writers and recipients may vary. This study took a pragmatic approach to realist evaluation [17, 27, 28] in order to apply realist logic to multiple perspective cases within single discharge events. The study drew on realist principles to generate a "programme theory" or theorised explanation of whether or not patients receiving letters "works" (or not) as well as outlining the important relating context [C], mechanism [M], and outcome [O] configurations (CMOCs). The programme theory from our previously conducted realist review [14] was used as the starting theory; this was further developed based on the primary data results and findings. Interrogation and synthesis of evidence for CMOCs used a realist analytic approach [18] to consider the same theory of whether or not "patients receiving letters" works in comparative settings [29]. Thus, analysis was grounded on the assumption that "outcomes" of the intervention may vary according to "context" [29]. All data were inspected for evidence of *"relevance"* [20, 29, 30] to the theory. Manual note-taking on data were then undertaken [14] and judgements were formed as to what any new CMOCs might plausibly be prior to integration into the programme theory.

Data relating to each group was initially analysed separately (see box 1). Findings across groups were then triangulated and a secondary analysis was undertaken using meta-matrices to compare and contrast data. Such triangulation has previously been used within healthcare research [31, 32], particularly in relation to healthcare consultations [33-35], to compare multiple perspectives. Multi-perspective case analysis involved re-review of the data for each case; findings from different participants within letter cases were re-read and juxtaposed to highlight agreements and disagreements. Narrative summaries for each case were then developed. Summaries were not intended to be comprehensive but select and include findings of relevance to the research questions. Analysis sought to reconcile previously identified literature disparities on this topic (see our realist review [14]) through highlighting source convergence and divergence in relation to "patients receiving letters".

For peer review only - http://bmjopen.bmj.com/site/about/guidelines.xhtml

Patient and public involvement

Around 30 patients were involved in the research design through identifying research priorities [36] by "ranking" potential research questions through completing surveys and taking part in discussions. Four persons with experience as carers from a pre-established panel also provided feedback on the readability and clarity of the patient information materials.

RESULTS

Recruitment

Figure 2 shows how data collection across all studies for the discharge communication project led to the formation of 26 trio cases (1 GP and HP, 3 patient and HP, 22 patient and GP) and 10 quartet cases (patient, GP, and HP). Table 2 summarises the data characteristics in terms of GP grading, patient gender and age, discharge episode type (inpatient, outpatient…), specialty, and hospital practitioner grade. The 10 quartet cases had an even divide of GP graded successful and unsuccessful letters. Four patients reported that they had previously received the discharge letter and six reported that they had not. Letters related to 6 specialties across four hospital trusts.

Table 2 trio and quartet characteristics

Characteristic	Trio cases (n=26)	Quartet cases (n=10)
GP grading	Successful: 18 (69.2%)<br>Unsuccessful: 8 (30.8%)	Successful: 5 (50.0%)<br>Unsuccessful: 5 (50.0%)
No. of GP practices and GPs	14 GP practices, 17 GPs	8 practices 9 GPs
Practice sizes	Small (<5,000 patients): 1 (7.1%)<br>Medium (5-10,000 patients): 8 (57.2%)<br>Large (10,000+ patients): 5 (35.7%)	Small (<5,000 patients): 0 (0.0%)<br>Medium (5-10,000 patients): 4 (50.0%)<br>Large (10,000+ patients): 4 (50.0%)
Patient age	Range: 27-87<br>Median: 67	Range: 59-77<br>Median: 71
Patient gender	Female: 14 (53.8%)<br>Male: 12 (46.2%)	Female: 3 (30.0%)<br>Male: 7 (70.0%)
Admission	Inpatient: 20 (76.9%)<br>Outpatient: 2 (7.7%)<br>Other*: 4 (15.4%)	Inpatient: 7 (70.0%)<br>Outpatient: 1 (10.0%)<br>Other*: 2 (20.0%)
Specialties	1. Urology: 2 (7.7%)<br>2. Respiratory: 1 (3.8%)<br>3. Accident & Emergency: 4 (15.5%)<br>4. General Surgery: 3 (11.5%)<br>5. Cardiology: 2 (7.7%)	1. Urology: 3 (30.0%)<br>2. Respiratory: 2 (20.0%)<br>3. Accident & Emergency: 1 (10.0%)<br>4. General Surgery: 2 (20.0%)<br>5. Cardiology: 1 (10.0%)<br>6. Trauma & Orthopaedics: 1 (10.0%)

For peer review only - http://bmjopen.bmj.com/site/about/guidelines.xhtml

	6. Trauma & Orthopaedics: 4 (15.5%)<br>7. Head and Neck: 1 (3.8%)<br>8. Endocrinology: 1 (3.8%)<br>9. Plastic Surgery: 1 (3.8%)<br>10. Neurosurgery: 1 (3.8%)<br>11. General Medicine: 4 (15.5%)<br>12. Internal Medicine: 1 (3.8%)<br>13. Renal Medicine: 1 (3.8%)	
Hospital grade of discharging physician	2 grade types:<br>Consultant: 20 (76.9%)<br>Core trainee or equivalent: 6 (23.1%)	4 grade types:<br>Consultant: 6 (60%)<br>Advanced clinical practitioner: 1 (10%)<br>Junior doctor: 2 (20%)<br>Senior house officer: 1 (10%)

**other may include but not be limited to admission types such as accident and emergency visit, day case procedure, or speciality assessment unit visit.*

Context mechanism outcome configurations

Narrative summaries for our data are in supplementary file 4 (trios) and 5 (quartets). Following a realist approach, findings were interrogated for theories and CMOCs of "relevance" [20, 29, 30] to patients receiving discharge letters. The following section describes the identified CMOCs and concepts. Sub-heading themes which structured our realist review [14] were used and iteratively modified. The 48 CMOCs from the realist review were also systematically interrogated in light of the new evidence; 7 new CMOCs were added. The final table of 55 CMOCs is in supplementary file 6.

Patient preference/choice

Of the 36 cases, 26 patients had received the discharge letter and 10 had not. Patients frequently emphasised positive effects of receiving letters such as increased satisfaction and a sense of involvement [12, 37] [CMOC2]. Patients explained that receiving letters can increase their autonomy and so encourage them to take control and *"ownership"* of their health [CMOC5, CMOC14]. In cases where patients had not received letters (C-E, H-J), patients reported difficulty retaining information and feeling unclear about what happened, their condition and how to manage it. On the other hand, in cases where patients had received letters [context, C](A, B, F, G), patients reported feeling informed and finding the letter useful as a reminder [mechanism, M] of what happened to increase recall [38, 39] [outcome, O] [CMOC15] and decrease the need to memorise information [CMOC50].

For peer review only - http://bmjopen.bmj.com/site/about/guidelines.xhtml

Past studies, across a range of settings, report that patient preference for receiving letters is high (79%-97%) [38-45]; this study supports this finding as patients generally indicated preference for discharge letter receipt. Despite this, both GPs and patients noted the inconsistent practice of patients receiving letters. A potential suggested solution was for letters to contain a template "tick box" [C] as to whether or not the patient has been given a letter copy so that it can be audited [O] and increase awareness of the practice [M] [CMOC49]. One new CMOC that emerged was that patients may use the letter [M] as a record [C] for providing evidence for administrative proceedings [O] (e.g. benefits) [CMOC51] or for care within unfamiliar settings (e.g. holidays). Broadly, impacts on patients' experiences were framed as more positive when patients had received discharge letters and more negative when they had not. Crucially, positive outcomes were typically only triggered within key contexts (e.g. letter factually accurate [CMOC15]). Our realist review found patients generally did not object to social habits being included in the letter as long as it had relevance [14]; our findings here caveated this notion in that this information should also be phrased with neutral non-judgemental language [C] to reduce likelihood of upset [M] which could diminish wellbeing [O] [CMOC53]. Crucially, patient preference was not 100% and it is important to consider those who may not wish to receive letters [CMOC40] through acknowledgments of *patient choice* [12, 40-42] [CMOC41]. Moreover, some patients may want to receive letters some of the time but not for every single care episode; patients identified this may apply in cases of repeat admissions for the same condition [C] where letters may be repetitive and not helpful [M] and so not requested [O] [CMOC52]. Systems of letter receipt must therefore account for individual case variation.

Patient comprehension

Findings supported previous evidence [40, 44, 46, 47], that patients may understand their letters [M] leading to improved patient knowledge and recall [O] as well as patients feeling empowered to take responsibility for their own health and so carrying out recommendations [CMOC12-15, CMOC54]. However, letters are not always stylistically tailored to patients' needs due to the presence of medical jargon and acronyms. Within some cases (e.g. case 6), the patient and GP agreed that the patient would have benefitted from use of lay terms in the letter to unravel the medical jargon. Case 5 highlighted that unexplained acronyms should be avoided for the sake of both patient and GP comprehensibility. There is a risk that patients receiving letters [C] may increase appointments or queries [O] as patients seek explanations of the letter contents [M] [48]. Nevertheless, in line with past work [45, 49], findings

For peer review only - http://bmjopen.bmj.com/site/about/guidelines.xhtml

were that this rarely occurs and indeed no study patients reported having made appointments for this purpose [CMOC7, CMOC11]. Furthermore, patients reported that the absence rather than receipt of the letter is what would prompt them to visit the GP [M] and thus increased patient information [C] may reduce rather than increase appointments [O] [CMOC11]. GPs suggested use of a “patient information” section on the letter [C] which provides a letter synopsis in the form of a lay summary to increase understanding [M] and improve patient knowledge and satisfaction [O] [CMOC54]. Patients and GPs agreed that letters should complement rather than substitute verbal information. This is seen in case 17 where the letter communicates a serious diagnosis to the patient and they report being given no other information from the hospital. Hence, letters should only be provided in the context of adequate patient counselling so that the letter is not communicating new information.

Personalised or patient-directed discharge letters

Personalised letters may increase resource use and workload (44, 47, 50) [CMOC25]. There were disagreements as to whether it would be more beneficial for patients to receive a separate personalised letter or the same letter as the GP; some clinicians felt personalised letters may improve patient comprehension (e.g. case 1) whereas patients generally preferred to receive the same copy as the GP for transparency and reassurance (e.g. case 3, 22, 23)[CMOC26]. Patients did suggest letter improvements in cases where the clinicians rated the letter successfully (cases B, I); patients felt letters should contain more information regarding how they can improve their condition and recommended patient actions.

Patient harm

Clinicians sometimes had concerns that patients receiving letters may cause harm such as patient anxiety or confusion. However, clinician concern was expressed in several cases where the patients emphasised the benefits of discharge letter receipt (cases B, C, E, G, H). Patients suggested that receiving letters [C] may reduce negative outcomes through reassuring them and reducing or settling anxiety [M] thereby supporting their wellbeing [O] [CMOC39] (case 8). Instances which subverted this trend primarily related to the letter quality (e.g. letter inaccuracies caused stress). One patient found that clear written information in bad news contexts [C] was particularly useful [M] as it allowed them to make

For peer review only - http://bmjopen.bmj.com/site/about/guidelines.xhtml

an informed end of life plan [O]. Suggestions to reduce risk of harm included ensuring the content is wholly factual and ensuring the patient consents to letter receipt [51] [CMOC41].

Clinician views

Supporting past literature, some clinicians were in favour [49, 52] [CMOC5, CMOC16] of the practice whilst others had reservations [12, 46] [CMOC6, CMOC35]. GPs appeared to be more in favour than hospital practitioners. Nonetheless, some GPs did express issues regarding the inherent need of letters to contain technical information which may not be patient comprehensible. Hospital practitioner concerns included: patient confusion and anxiety [13, 37, 43] [CMOC19], that the patient will not find the letter useful, that letters would need to be oversimplified [12, 53], and that receiving a letter may not be in the best interests of the patient (e.g. mental health cases). Clinician and GP perceived benefits [CMOC5] of patients receiving letters were: increased sense of patient inclusion, improved understanding or knowledge [50, 53], and increased transparency [46] [CMOC33]. Our realist review [14] suggested that patient understanding of their letters may be higher than clinicians perceive; this study further supports this notion. Comparably to previous literature, concern regarding "patient understanding" was common [12, 37, 46, 53] [CMOC6]. However, clinician and patient views were sometimes the antithesis of one another; there were cases where the clinician had concerns [C] regarding patient comprehensibility [M] in cases where the patient reported to have found the letter useful [O][CMOC55] (see cases A-C, E, G-H, J). Patients demonstrated resourcefulness through expounding that unknown terms can be looked up on the internet (case 19) as well as discretion [C] through appreciating that understanding the contents and implications [O] may not necessarily involve comprehending every word [M].

Programme theory

Our findings were used to refine the programme theory, using our realist review [14] as the starting point; changes made to the theory are highlighted in bold (see figure 3). All matched cases were re-read, annotated and interrogated for evidence. Relevant evidence [29, 30] was inspected and concepts drawn on to form the resultant programme theory in figure 3 which shows two main channels: patient copies of letters and patient personalised letters. Contexts for when patients receive letters still contained five key contexts for when this

For peer review only - http://bmjopen.bmj.com/site/about/guidelines.xhtml

intervention does work but context details were modified. Previously, the theory had four key contexts for when the intervention is theorised not to work; these were updated to include the new context of judgemental language in relation to social behaviour [CMOC53]. Outcomes of patients receiving separate personalised letters were modified; new negative outcomes were overly "basic" content and perceived potential secrecy between clinicians if they are sending and receiving separate letters. "Patient choice" was still a key influencer for likelihood of beneficial outcomes, and contextual influences such as resource provision and directives [CMOC49] were determiners of patients being given a choice of letter receipt [CMOC52].

DISCUSSION

Summary of findings

We undertook a realist evaluation (19, 27, 54, 55) to explore patient, GP and hospital clinician experiences of written discharge communications and hence test and refine the programme theory from our previous realist review (14). The modified programme theory (figure 3) maps out how patients receiving discharge letters works in specific contexts leading to different positive and negative outcomes. Positive outcomes and positive pathway components are indicated in figure 3 via green coloured text boxes whereas negative outcomes and negative pathway components are indicated in red. Any neutral components or those which can be either positive or negative (e.g., attitudes of clinicians) are in black. Analysis of the multi-perspective discharge events led to the emergence of findings not found in our previous review. Several changes to the initial theory were made to include 10 CMOC modifications and the addition of 7 new CMOCs not found through previous literature searching. No CMOCs were removed. Key contexts for positive outcomes included: letters written in plain English, lay explanations for jargon, written and verbal information provided, no new information in letter, and patient given choice of letter receipt.

While benefits (41, 56) and drawbacks (53, 57) of patients receiving discharge letters have been previously suggested, our study adds an understanding of *how* patients receiving letters *works* through outlining the important contexts and associated mechanisms that explain outcome patterns (58, 59). In addition, the multi-perspective analysis provided possible explanations for previously reported discrepancies identified through our realist review (14). One example of a discrepancy was that past work highlighted conspicuously inconsistent rates of patient understanding (12, 40, 46, 47, 60, 61). Data from this study revealed that even in cases where clinicians expressed concerns, patients generally reported to have understood

For peer review only - http://bmjopen.bmj.com/site/about/guidelines.xhtml

the letter and found it useful. Furthermore, patients often preferred receiving the same letter as the GP rather than a separate letter. Another disparity was in relation to "negative outcomes". A common clinician concern within the study and past literature [13, 37, 43] was that patients receiving letters may cause anxiety and harm. However, literature also reported that patients may find letters useful [12, 44, 47]. Our method highlighted that in several cases where clinicians had concerns, patients who received letters tended to emphasise the positive effects (e.g. increased knowledge). Indeed, patients stressed negative outcomes in contexts where they *had not* rather than *had* received letters. Some patients reported that receiving the letter alleviated anxiety thereby supporting their wellbeing through informing them of their admission, and any next steps, as well as providing reassurance that their GP was updated.

Strengths and weaknesses of the study

We followed RAMESES standards for realist evaluation [28, 62] and completed the COREQ checklist by Tong et al. [63]. To the best of our knowledge, this is the first study to triangulate matched perspectives of patients and clinicians in relation to specific discharge letters. This allowed reconciliation of disparities in the literature and so enabled refinement of the programme theory. Grounding the research in realist theory strengthened the applicability of findings as it facilitated an understanding of not just whether patients should receive letters, but how this practice may "work" as well as in what contexts and why [16, 17].

As with other realist evaluations [64], the results and findings are intended to have wide applicability to other settings, in this case, settings where adults may receive hospital discharge letters. However, it is important to note the contexts and those groups who were excluded or were under-represented in this study. The exclusion criteria restricted the programme theory such that evidence relating to children, solely to mental health, and those lacking capacity to consent was not considered. Moreover, participation bias may have resulted in the views of ethnic minorities and other marginalised groups being under-represented. The main weakness of the study was the small sample sizes in terms of numbers of patients, sociodemographic diversity of the patients, and range of conditions; for many of the discharge letters it was not possible to form a complete quartet. The study fell short of the target of building 30 quartets; the primary reason for this was under-recruitment of hospital practitioners. The low response rate of hospital practitioners was likely impacted by their lack of available time, our survey recruitment strategy, hospital rotations, and the time lapse between the practitioner writing the letter and receiving the survey invitation. The programme theory would have benefitted from being informed by a larger and more diverse sample of primary evidence. The matched cases relate to a specific geographic area and

For peer review only - http://bmjopen.bmj.com/site/about/guidelines.xhtml

hence will not have reflected the full range of hospital discharge communication practices that are present nationally. Analysis cannot be considered to be wholly objective due to the influence of researcher identity [65]. Therefore, “reflexivity” was practised throughout the research to reduce but not eradicate bias [65, 66]. Reflexivity was practised through keeping a research diary and regular research team discussion and reflection. Data analysis was also limited by the available evidence which was thin in relation to: dictating letters, the cost of patients receiving letters, doctor-patient relationships, and reasons for variation of practice. Further research is needed to explore these areas as well as the relevance of the programme theory to excluded and under-represented groups, such as those without capacity and children.

Meaning of the study: implications for clinicians and policy makers

The programme theory generated by this study draws on our previous review and primary data, and hence reflects evidence from 16 countries and over 16,000 participants. As such, the theory has both national and international relevance and is likely to be applicable to different healthcare settings. It generally supports policies [7-9, 11] that patients should be offered copies of letters between physicians. Although sending patients’ letters, to include discharge letters, has been recommended practice for almost 20 years [7], uptake remains inconsistent [12-14]. Although national guidelines exist [7-10, 67, 68], each hospital may have its own discharge policy; this means that patients may have different discharge experiences and receive different discharge communications depending on the hospital, discharging physician, and reason for admission, as exemplified in this study. This needs to be addressed with more standardised practices which account for individual preferences and are grounded by *patient choice* with the exception of where there is a risk of “harm”, as defined in guideline documents [7]. Patients have a right to receive their letters [11] and should not be denied the opportunity to receive letters based on the perception that their understanding may be low. Although patients may have limited health literacy, they demonstrated resourcefulness and resilience for accessing letter content by looking up unknown terms on the internet and also appreciated that understanding the important features and main directives of a letter does not necessarily involve comprehending every word. Thus, patient understanding is perhaps greater than perceived and the presence of clinical terminology alone is not reason enough to exclude patients from communications. Overall, our study found that negative outcomes more commonly manifested when patients had not received letters, rather than when they had. This included contexts where the clinicians had concern about patient understanding and yet the patient reported to have

For peer review only - http://bmjopen.bmj.com/site/about/guidelines.xhtml

found the letter of value. It may be inferred that within certain contexts, clinician concerns about patients receiving letters are perhaps unfounded. Thus, clinician attitudes and risk averse behaviour may be acting as a barrier to uptake of this practice.

This research has provided a modified programme theory which demonstrates how policy makers and clinicians may effectively involve patients in their care through provision of written communications. Our theory outlines how both positive and negative outcomes may be produced through this intervention and highlights the importance of contextual considerations [55]. As outlined in previous realist evaluations [59], an advantage of this approach is the relevance of the resultant theory to policy makers as it informs how policy may be adapted to particular purposes and the specific contexts needed to achieve the desired outcomes. An example is the importance of the contextual factor “patient choice of letter receipt” to producing positive outcomes; this is of relevance to policy makers as it explains how best practice of patients receiving letters may be adapted to “work” and how research may be implemented into practice and policy. Nonetheless, future work should endeavour to test and refine the programme theory through interrogation of new evidence and measurement of primary and secondary outcomes. This will support the development of interventions that lead to more effective communication between hospital and primary care health professionals, and hence positive patient outcomes.

CONCLUSION

Sharing information and effective discharge communication with patients should be a priority to improve patient experience and the safety of patient care. This study has yielded insights into ways in which practices of patients receiving discharge letters could be improved to enhance patient experience and outcomes. Key findings were: clinicians may underestimate patients’ capacity to comprehend discharge letters, patient choice is important for positive outcomes, absence rather than presence of information may be more associated with negative outcomes, and clinician attitudes may be acting as a barrier to patients receiving letters. Our programme theory draws on previous research and experiences of clinicians and patients. The theory has potential for use in different healthcare contexts and as a framework for policy development on patient discharge.

Acknowledgements: The authors would like to express thanks to the GPs, patients, and hospital practitioners who informed the study design as well as those who took part in the study. We would like to thank the West Midlands Clinical Research Network for their support with research recruitment. We would also like to thank the Institute of Advanced Study at the

For peer review only - http://bmjopen.bmj.com/site/about/guidelines.xhtml

University of Warwick for their support during the write-up of this paper. Finally, we would like to thank Hugh Rayner for providing feedback on an earlier version of the manuscript.

Author contributions: KW, ES, SS and JD substantially contributed to the design and conception of the work and collaborated with KW on data interpretation. KW drafted the manuscript which was then critically revised by JD, ES and SS. All authors approved the final manuscript for publication and agree to be accountable for all aspects of the work.

Funding: This work was supported by the Economic and Social Research Council [grant number ES/J500203/1] and Clinical Commissioning Groups of Coventry & Rugby and South Warwickshire. Funding for the open access charges for publication of this manuscript was provided by the UKRI fund. The funders had no involvement in analysis or preparation of the manuscript.

Competing interests: None declared.

Patient and public involvement: The public and other stakeholder groups were involved in the design and selection of research questions for this research.

Patient consent for publication: Not required.

Ethics approval: Ethics approval was granted by the UK Health Research Authority in July 2017 (Integrated Research Application System ID: 219871, Research Ethics Committee reference: 17/WM/0170, sponsor: University of Warwick).

Data sharing statement: The narrative summaries for data are provided as supplementary files. The full raw datasets for the study are not publicly available due to the sensitive and identifiable nature of the data. Despite names and other identifiers being removed, the in-depth nature of case information may mean that participants could be identified.

References

1.Groene R.O., Orrego C., Sunol R., *et al.* "It's like two worlds apart": an analysis of vulnerable patient handover practices at discharge from hospital. *BMJ Qual Saf*. 2012;21 Suppl 1:i67-75.[Accessed: 10/06/20].
2.Lorenzati B., Quaranta C., Perotto M., *et al.* Discharge communication is an important underestimated problem in emergency department. *Internal & Emergency Medicine*. 2016;11(1):157-8. Available from: https://dx.doi.org/10.1007/s11739-015-1351-0 [Accessed: 09/07/2020].
3.Rapport F., Hibbert P., Baysari M., *et al.* What do patients really want? An in-depth examination of patient experience in four Australian hospitals. *BMC Health Serv Res*. 2019;19(1):38.[Accessed: 17/07/2020].
4.Flink M., Bergenbrant Glas S., Airosa F., *et al.* Patient-centered handovers between hospital and primary health care: an assessment of medical records. *Int J Med Inform*. 2015;84(5):355-62.[Accessed: 10/06/20].
5.Beaton A., O'Leary K., Thorburn J., *et al.* Improving patient experience and outcomes following serious injury. *N Z Med J*. 2019;132(1494):15-25.[Accessed: 10/06/20].
6.Rayner H., Hickey, M., Logan, I., Mathers, N., Rees, P., Shah, R. Writing outpatient letters to patients. *BMJ*. 2020;368:m24. Available from: https://www.bmj.com/content/bmj/368/bmj.m24.full.pdf [Accessed: 10/07/2020].
7.*Department of Health*. Copying letters to patients: good practice guidelines. 2003. Available from https://webarchive.nationalarchives.gov.uk/20120504030618/http://www.dh.gov.uk/pr

For peer review only - http://bmjopen.bmj.com/site/about/guidelines.xhtml

od_consum_dh/groups/dh_digitalassets/@dh/@en/documents/digitalasset/dh_4086054.pdf

8.The Academy of Medical Royal Colleges. Please, write to me: Writing outpatient clinic letters to patients. 2018. Available from https://www.aomrc.org.uk/reports-guidance/please-write-to-me-writing-outpatient-clinic-letters-to-patients-guidance/

9.National Institute for Health and Care Excellence (NICE). Patient experience in adult NHS services: improving the experience of care for people using adult NHS services 2012. Available from: https://www.nice.org.uk/guidance/cg138.

10.Professional Record Standards Body. Implementation guidance report eDischarge standard. Better records for better care2019. Available from https://theprsb.org/standards/healthandcarerecords/

11.*Department of Health*. The NHS Plan: A Plan for Investment a Plan for Reform. London: HMSO; 2000. Available from https://webarchive.nationalarchives.gov.uk/20121102184216/http://www.dh.gov.uk/en/Publicationsandstatistics/Publications/PublicationsPolicyAndGuidance/DH_4002960

12.Baxter S., Farrell K., Brown C., *et al.* Where have all the copy letters gone? A review of current practice in professional-patient correspondence. *Patient Educ Couns*. 2008;71(2):259-64. Available from: https://dx.doi.org/10.1016/j.pec.2007.12.002 [Accessed: 10/06/20].

13.Boaden R., Harris C. Copying letters to patients—will it happen? *Fam Prac*. 2005;22:141–3. Available from: https://academic.oup.com/fampra/article/22/2/141/522310 [Accessed: 09/07/2020].

14.Weetman K., Wong G., Scott E., *et al.* Improving best practice for patients receiving hospital discharge letters: a realist review. *BMJ Open*. 2019;9(6):e027588. Available from: https://bmjopen.bmj.com/content/bmjopen/9/6/e027588.full.pdf [Accessed: 10/06/20].

15.Harris E., Rob P., Underwood J., *et al.* Should patients still be copied into their letters? A rapid review. *Patient Educ Couns*. 2018;101(12):2065-82. Available from: https://www.sciencedirect.com/science/article/abs/pii/S0738399118303136?via%3Dihub [Accessed: 09/07/2020].

16.Pawson R. Middle range theory and program theory evaluation: From provenance to practice. In: Vaessen J., Leeuw, F.L., editor. *Mind the gap Perspectives on policy evaluation and the social sciences*. New Brunswick, NJ and London: Transaction Publishers; 2010. p. 171-203.

17.Pawson R. *The science of evaluation: a realist manifesto*. London: Sage; 2013.

18.Pawson R. *Evidence-based policy: a realist perspective*. London, UK: Sage; 2006.

19.Pawson R., & Tilley, N. *An introduction to scientific realist evaluation.* 1997 2020/09/02. In: Evaluation for the 21st Century: A Handbook [Internet]. Thousand Oaks, California: SAGE Publications, Inc.; [405-18]. https://methods.sagepub.com/book/evaluation-for-the-21st-century.

20.Pawson R., Greenhalgh T., Harvey G., *et al.* Realist review--a new method of systematic review designed for complex policy interventions. *J Health Serv Res Policy*. 2005;10 Suppl 1:21-34. Available from: https://journals.sagepub.com/doi/abs/10.1258/1355819054308530 [Accessed: 09/07/2020].

21.Weetman K., Dale J., Scott E., *et al.* The Discharge Communication Study: research protocol for a mixed methods study to investigate and triangulate discharge communication experiences of patients, GPs, and hospital professionals, alongside a corresponding discharge letter sample. *BMC Health Services Research*. 2019;19(1):825. Available from: https://doi.org/10.1186/s12913-019-4612-1 [Accessed: 10/06/20].

22.Weetman K., Dale J., Spencer R., *et al.* GP perspectives on hospital discharge letters: an interview and focus group study. *BJGP Open*. 2020

For peer review only - http://bmjopen.bmj.com/site/about/guidelines.xhtml

https://bjgpopen.org/content/4/2/bjgpopen20X101031. Available from: https://bjgpopen.org/content/4/2/bjgpopen20X101031 [Accessed: 10/06/20].
23. Weetman K., Dale J., Scott E., *et al.* Adult patient perspectives on receiving hospital discharge letters: a corpus analysis of patient interviews. *BMC Health Services Research*. 2020;20(1):537. Available from: https://doi.org/10.1186/s12913-020-05250-1 [Accessed: 24/6/20].
24. Teddlie C., Yu F. Mixed methods sampling: A typology with examples. *Journal of mixed methods research*. 2007;1(1):77-100. Available from: https://journals.sagepub.com/doi/10.1177/1558689806292430 [Accessed: 09/07/2020].
25. Stuckey H.L. Three types of interviews: Qualitative research methods in social health. *J Diabetes Res & Clin Prac*. 2013;1(2):56.[Accessed].
26. Pawson R., Tilley N. *Realistic Evaluation. Evaluation*. London: Sage; 1999.
27. Jagosh J., Bush P.L., Salsberg J., *et al.* A realist evaluation of community-based participatory research: partnership synergy, trust building and related ripple effects. *BMC Public Health*. 2015;15:725. Available from: https://pubmed.ncbi.nlm.nih.gov/26223523/ [Accessed: 21/7/2020].
28. Wong G., Westhorp G., Greenhalgh J., *et al.* Quality and reporting standards, resources, training materials and information for realist evaluation: the RAMESES II project. *NIHR Journals*. 2017;Health Services and Delivery Research(5). Available from: https://pubmed.ncbi.nlm.nih.gov/29072890/ [Accessed: 21/7/2020].
29. Pawson R., Greenhalgh, T., Harvey, G. & Walshe, K. . Realist synthesis: an introduction.' *ESRC Research Methods Programme*. 2004 Available at: https://goo.gl/1Rz2Ry. Available from: Available at: https://goo.gl/1Rz2Ry [Accessed: 04/01/17].
30. Pawson R. Digging for nuggets: how 'bad' research can yield 'good' evidence. *International Journal of Social Research Methodology*. 2006;9(2):127-42. Available from: https://www.tandfonline.com/doi/abs/10.1080/13645570600595314 [Accessed: 09/07/2020].
31. Farmer T., Robinson, K.. Elliott, S. J., Eyles, J. Developing and implementing a triangulation protocol for qualitative health research. *Qual Health Res*. 2006;16(3):377-94. Available from: https://journals.sagepub.com/doi/abs/10.1177/1049732305285708 [Accessed: 09/07/2020].
32. Begley C.M. Using triangulation in nursing research. *Journal of Advanced Nursing*. 1996;24(1):122-8. Available from: https://onlinelibrary.wiley.com/doi/abs/10.1046/j.1365-2648.1996.15217.x [Accessed: 09/07/2020].
33. Mendick N., Young, B., Holcombe, C., Salmon, P. The ethics of responsibility and ownership in decision-making about treatment for breast cancer: triangulation of consultation with patient and surgeon perspectives. *Soc Sci Med*. 2010;70(12):1904-11. Available from: https://www.sciencedirect.com/science/article/abs/pii/S027795361000225X?via%3Dihub [Accessed: 09/07/2020].
34. Salmon P., Mendick, N., Young, B. Integrative qualitative communication analysis of consultation and patient and practitioner perspectives: towards a theory of authentic caring in clinical relationships. *Patient Educ Couns*. 2011;82(3):448-54. Available from: https://www.sciencedirect.com/science/article/abs/pii/S0738399110006257?via%3Dihub [Accessed: 09/07/2020].
35. Durif-Bruckert C., Roux, P., Morelle, M., Mignotte, H., Faure, C., Moumjid-Ferdjaoui, N. Shared decision-making in medical encounters regarding breast cancer treatment: the contribution of methodological triangulation. *Eur J Cancer Care (Engl)*. 2015;24(4):461-72. Available from: https://onlinelibrary.wiley.com/doi/abs/10.1111/ecc.12214 [Accessed: 09/07/2020].

For peer review only - http://bmjopen.bmj.com/site/about/guidelines.xhtml

36.Cowan K. O., S. James Lind Alliance Guidebook. Southampton: James Lind Alliance; 2013.
37.Tomkins C.S., Braid J.J., Williams H.C. Do dermatology outpatients value a copy of the letter sent to their general practitioner? In what way and at what cost? *Clin Exp Dermatol*. 2004;29(1):81-6. Available from: https://onlinelibrary.wiley.com/doi/abs/10.1111/j.1365-2230.2004.01437.x?sid=nlm%3Apubmed [Accessed: 10/06/20].
38.Antoniou A., Saunders M., Bourner R., *et al.* would you like to see yours? *Bull R Coll Surg Engl*. 2007;89(2):62-4. Available from: https://publishing.rcseng.ac.uk/doi/10.1308/147363507X169936 [Accessed: 09/07/2020].
39.Krishna Y., Damato B.E. Patient attitudes to receiving copies of outpatient clinic letters from the ocular oncologist to the referring ophthalmologist and GP. *Eye (Lond)*. 2005;19(11):1200-4. Available from: https://www.nature.com/articles/6701740 [Accessed: 09/07/2020].
40.Fenton C., Al-Ani A., Trinh A., *et al.* Impact of providing patients with copies of their medical correspondence: a randomised controlled study. *Intern Med J*. 2017;47(1):68-75. Available from: https://onlinelibrary.wiley.com/doi/abs/10.1111/imj.13252 [Accessed: 10/06/20].
41.O'Driscoll B.R., Koch J., Paschalides C. Copying letters to patients: Most patients want copies of letters from outpatient clinics and find them useful. *BMJ*. 2003;327(7412). Available from: http://www.ncbi.nlm.nih.gov/pmc/articles/PMC188516/ [Accessed: 10/07/2020].
42.Rao M., Fogarty P. What did the doctor say? *J Obstet Gynecol*. 2007;27(5):479-80. Available from: https://dx.doi.org/10.1080/01443610701405853 [Accessed: 09/07/2020].
43.Treacy K., Elborn J.S., Rendall J., *et al.* Copying letters to patients with cystic fibrosis (CF): letter content and patient perceptions of benefit. *J Cyst Fibros*. 2008;7(6):511-4. Available from: https://www.cysticfibrosisjournal.com/article/S1569-1993(08)00061-1/fulltext [Accessed: 10/06/20].
44.Brodie T., Lewis D. A survey of patient views on receiving vascular outpatient letters. *Eur J Vasc Endovasc Surg*. 2010;39(1):5-10. Available from: https://www.ejves.com/article/S1078-5884(09)00500-0/fulltext [Accessed: 09/07/2020].
45.Sharma D., O'Brien S., Hardy K. Copying letters to patients: What patients think - A questionnaire survey. *Clinician in Manage*. 2007;15(2):75-8. Available from: https://www.ncbi.nlm.nih.gov/pmc/articles/PMC2121286/ [Accessed: 09/07/2020].
46.Baumann W., Schussler, L., Bertram, M., Benser, J., Kumpers, S., Hermes-Moll, K. Oncologists' letters for breast cancer patients. *Oncol Res Treat*. 2016;39:184-5. Available from: http://dx.doi.org/10.1159/000449050 [Accessed: 10/06/20].
47.Pinder E., Jefferys S., Loeffler M. Patient Satisfaction: Receiving a copy of the GP letter following fracture or elective orthopaedic clinic. *BMJ Qual Improv Rep*. 2013;2(2). Available from: http://bmjopenquality.bmj.com/content/bmjqir/2/2/u202144.w1085.full.pdf [Accessed: 09/07/2020].
48.Liapi A., Robb P.J., Akthar A. Copying clinic letters to patients: a survey of patient attitudes. *J Laryngol Otol*. 2006;121(6):588-91. Available from: https://www.cambridge.org/core/article/copying-clinic-letters-to-patients-a-survey-of-patient-attitudes/9683993BFBE9720C5C9C13741F285713 [Accessed: 17/07/2020].
49.Brockbank K. Copying patient letters - Making it work. *Clin Gov*. 2005;10(3):231-40. Available from: http://dx.doi.org/10.1108/14777270510627590 [Accessed: 10/06/20].
50.McConnell D., Butow P., Tattersall M. Audiotapes and letters to patients: the practice and views of oncologists, surgeons and general practitioners. *Br J Cancer*. 1999;79:1782-8.[Accessed: 10/06/20].

For peer review only - http://bmjopen.bmj.com/site/about/guidelines.xhtml

51.The Newcastle upon Tyne Hospitals NHS Foundation Trust. The Newcastle upon Tyne Hospitals NHS Foundation Trust: Sharing Letters with Patients Policy. 2019. Available from http://www.newcastle-hospitals.org.uk/downloads/policies/Operational/SharingLetterswithPatients201901.pdf
52.Bench S.D., Heelas K., White C., *et al.* Providing critical care patients with a personalised discharge summary: a questionnaire survey and retrospective analysis exploring feasibility and effectiveness. *Intensive & Critical Care Nursing*. 2014;30(2):69-76. Available from: http://www.intensivecriticalcarenursing.com/article/S0964-3397(13)00090-6/fulltext [Accessed: 17/07/2020].
53.O'Reilly M., Cahill M., Perry I.J. Writing to patients: 'putting the patient in the picture'. *Ir Med J*. 2005;98(2):58-60. Available from: https://pubmed.ncbi.nlm.nih.gov/15835515/ [Accessed: 09/07/2020].
54.Martin P., Tannenbaum, C. A realist evaluation of patients' decisions to deprescribe in the EMPOWER trial. *BMJ Open*. 2017;7(4):e015959. Available from: https://bmjopen.bmj.com/content/bmjopen/7/4/e015959.full.pdf [Accessed: 09/09/2020].
55.Crampton P., Mehdizadeh L., Page M., *et al.* Realist evaluation of UK medical education quality assurance. *BMJ Open*. 2019;9(12):e033614. Available from: https://bmjopen.bmj.com/content/bmjopen/9/12/e033614.full.pdf [Accessed: 09/09/2020].
56.Saunders N.C., Georgalas C., Blaney S.P., *et al.* Does receiving a copy of correspondence improve patients' satisfaction with their out-patient consultation? *J Laryngol Otol*. 2003;117(2):126-9. Available from: https://dx.doi.org/10.1258/002221503762624576 [Accessed: 09/07/2020].
57.Hallowell N. Providing letters to patients. Patients find summary letters useful. *BMJ*. 1998;316(7147):1830. Available from: https://www.bmj.com/content/316/7147/1830.3 [Accessed: 09/07/2020].
58.Kerr H., Price, J., Nicholl, H., O'Halloran, P. Facilitating transition from children's to adult services for young adults with life-limiting conditions (TASYL): Programme theory developed from a mixed methods realist evaluation. *International Journal of Nursing Studies*. 2018;86:125-38. Available from: http://www.sciencedirect.com/science/article/pii/S0020748918301536 [Accessed: 09/09/2020].
59.Willis C.E., Reid S., Elliott C., *et al.* A realist evaluation of a physical activity participation intervention for children and youth with disabilities: what works, for whom, in what circumstances, and how? *BMC Pediatrics*. 2018;18(1):113. Available from: https://doi.org/10.1186/s12887-018-1089-8 [Accessed: 09/09/2020].
60.Lin M.J., Tirosh A.G., Landry A. Examining patient comprehension of emergency department discharge instructions: Who says they understand when they do not? *Internal & Emergency Medicine*. 2015;10(8):993-1002. Available from: https://dx.doi.org/10.1007/s11739-015-1311-8 [Accessed: 09/07/2020].
61.Choudhry A.J., Baghdadi Y.M., Wagie A.E., *et al.* Readability of discharge summaries: with what level of information are we dismissing our patients? *American Journal of Surgery*. 2016;211(3):631-6. Available from: https://dx.doi.org/10.1016/j.amjsurg.2015.12.005 [Accessed: 09/07/2020].
62.Wong G., Westhorp G., Manzano A., *et al.* RAMESES II reporting standards for realist evaluations. *BMC medicine*. 2016;14(1):96-. Available from: https://pubmed.ncbi.nlm.nih.gov/27342217 [Accessed: 21/7/2020].
63.Tong A., Sainsbury P., Craig J. Consolidated criteria for reporting qualitative research (COREQ): a 32-item checklist for interviews and focus groups. *Int J Qual Health Care*. 2007;19(6):349-57. Available from: https://academic.oup.com/intqhc/article/19/6/349/1791966 [Accessed: 09/07/2020].
64.De Sutter M., De Sutter, A., Sundahl, N., Declercq, T., Decat, P. Inter-professional collaboration reduces the burden of caring for patients with mental illnesses in

For peer review only - http://bmjopen.bmj.com/site/about/guidelines.xhtml

primary healthcare. A realist evaluation study. *European Journal of General Practice*. 2019;25(4):236-42. Available from: https://doi.org/10.1080/13814788.2019.1640209 [Accessed: 09/09/2020].

65. Malterud K. Qualitative research: standards, challenges, and guidelines. *The lancet*. 2001;358(9280):483-8. Available from: https://www.thelancet.com/journals/lancet/article/PIIS0140-6736(01)05627-6/fulltext [Accessed: 09/07/2020].

66. Mays N., Pope C. Qualitative research in health care: Assessing quality in qualitative research. *BMJ*. 2000;320(7226):50. Available from: https://www.ncbi.nlm.nih.gov/pmc/articles/PMC1117321/ [Accessed: 09/07/2020].

67. Royal College of Physicians. Standards for the clinical structure and content of patient records. 2013. Available from https://www.rcplondon.ac.uk/projects/outputs/standards-clinical-structure-and-content-patient-records

68. NHS Digital. The PRSB Standards for the Structure and Content of Health and Care Records. Professional Record Standards Body (PRSB); 2018. Available from https://theprsb.org/standards/healthandcarerecords/

List of figure headings

Figure 1 Multiple-perspective "quartet" case wherein comparisons occur between experiences associated with the same discharge letter

Figure 2 Recruitment uptake across studies for the project to show how trio and quartet cases were formed

Figure 3 Resultant programme theory that maps out how patients receiving discharge letters works (or not)

For peer review only - http://bmjopen.bmj.com/site/about/guidelines.xhtml

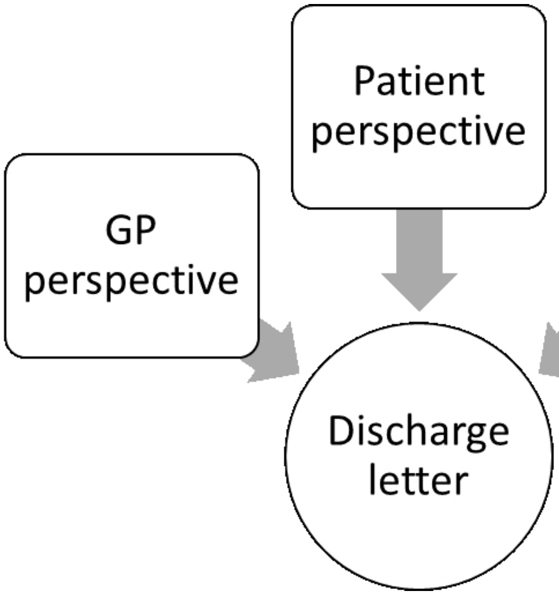

Figure 1 Multiple-perspective “quartet” case wherein comparisons occur between experiences associated with the same discharge letter

73x43mm (300 x 300 DPI)

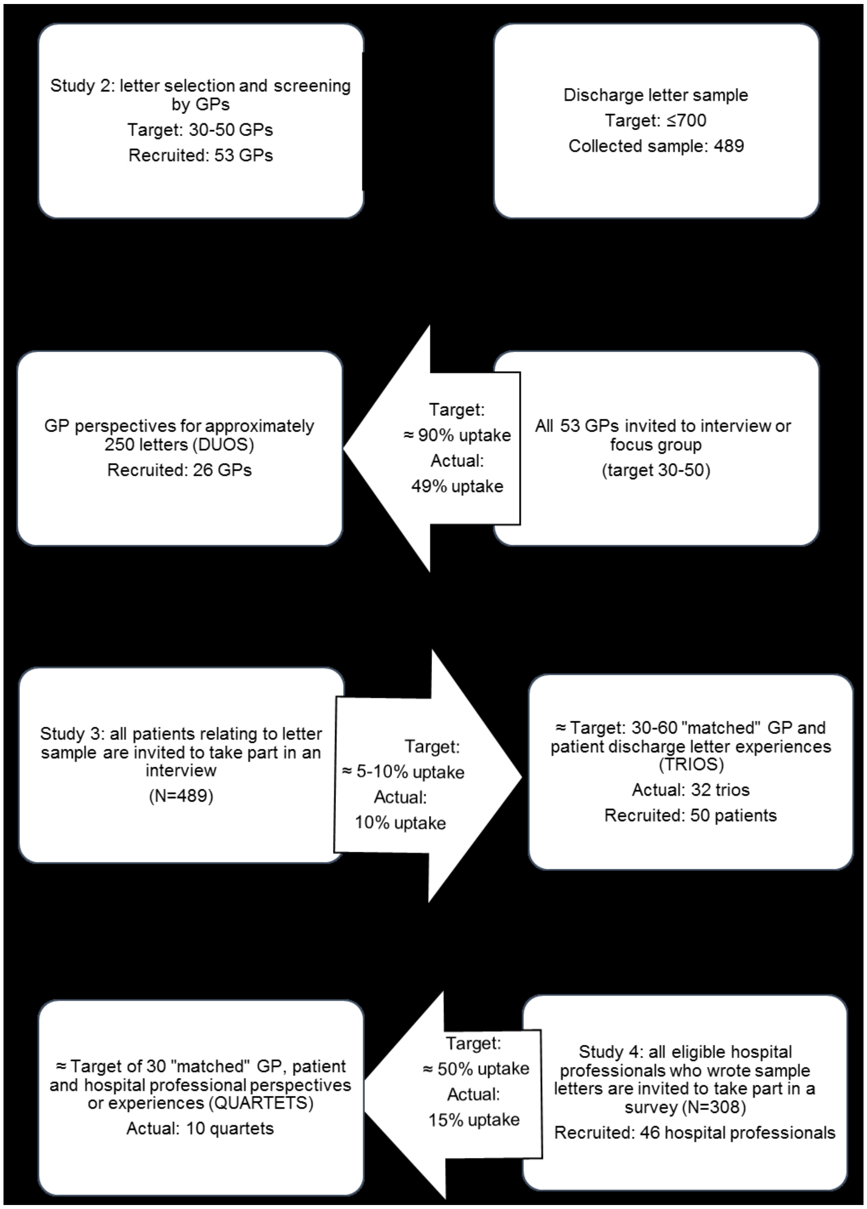

Figure 2 Recruitment uptake across studies for the project to show how trio and quartet cases were formed

85x119mm (300 x 300 DPI)

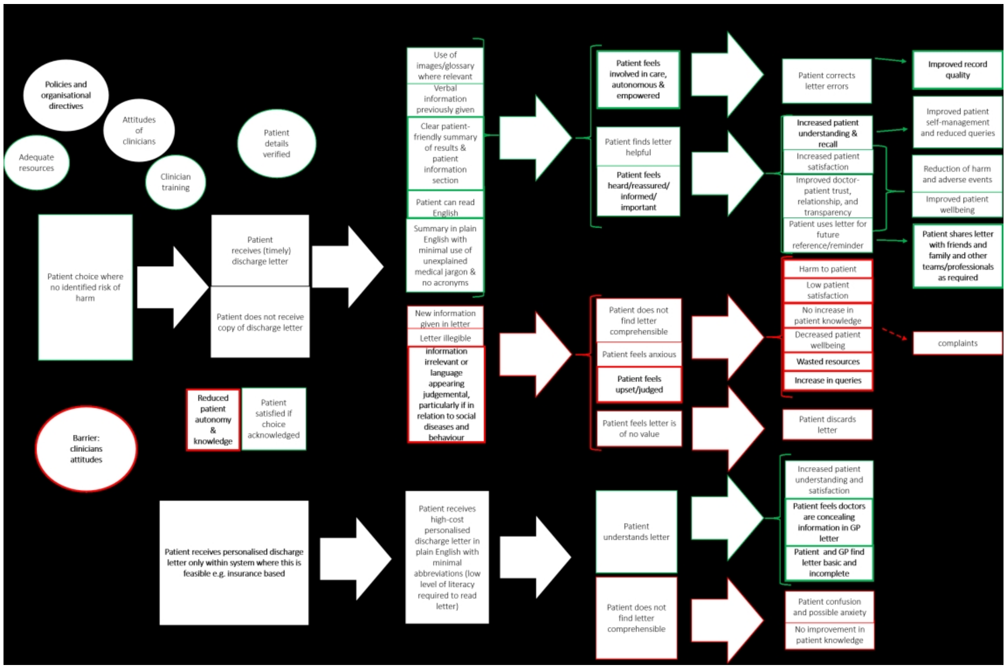

Figure 3 Resultant programme theory that maps out how patients receiving discharge letters works (or not)

234x155mm (300 x 300 DPI)

No. of letters selected	Patient name (to be removed during redaction)	Patient Unique research ID (to be added during redaction)	Categorisation (**Unsuccessful** OR **successful** discharge letter example)	Reason for selection & categorisation (e.g. any key good or bad points about letter)
EXAMPLE (Before redaction)<br><br>*(after redaction)*	*Mr Joe Smith*<br><br>[redacted]	 <br>*P0001*	*Unsuccessful*<br><br>*Unsuccessful*	*Bad points:*<br>*Medication alterations poorly outlined and information given to patient not explained*
1				
2				
3				

More rows to be added as needed…

For peer review only

GP interview and focus group guide

Interviewer opening question:

Please tell me about your experience(s) of patients receiving written discharge communication?

The rest of interview or focus group will continue in a conversational manner discussing GPs views and experiences on patients receiving written discharge communication and how the discharge communication process can be improved.

For peer review only

Possible interviewer prompts:

- What are your experiences of discharge communication as a GP?
- How do you think discharge communication can be improved?
- Please tell me your views on the discharge letters you selected?
- How would you suggest to improve these letters?
- In your opinions, is this letter suitable for a/the patient?
- What are your views on patients receiving letters?
- What do you think are important content items for good quality discharge letters?
- In your view what are the effects and outcomes of poor quality discharge letters?

Patient interview schedule

I: Interviewer (member of the research team) *Action points Q= Question

I: ***Q1: Please tell me about your experiences of receiving any form of written discharge communication? This can be either a direct copy of the letter sent to your GP or a discharge letter specifically addressed to yourself.***

Q2: When you were discharged from hospital on DATE, what information were your given?

****if patient able to be shown letter copy as per protocol, show patient their letter****

Q3: How did you feel about the information you were given?

Q4: What written information would you like to be given or sent when being discharged from hospital and why?

Q5: Would you prefer to receive a direct copy of the letter sent to your GP or a separate letter specifically addressed to yourself?

Q6: Would you like to always be given this letter or would you prefer to choose each time you are discharged?

Q7: How do you think the process of patients receiving written discharge communication can be improved?

Q8: Is there anything else you would like to talk to me about today related to written discharge communication?

Discussion may continue in a relaxed conversational manner and researcher may ask additional questions related to anything else relevant mentioned by the patient.

For peer review only - http://bmjopen.bmj.com/site/about/guidelines.xhtml

Trio meta-matrix with narrative summaries (S=successful, US=unsuccessful)

Trio case	GP grading	GP comment and interview/focus group findings	Patient interview findings	HP survey findings	Summary of main trio findings
1	S	Although GP graded letter successful due to clear diagnosis and findings, they did comment that the patient management plan was unclear. GP asserted that they felt patients should receive letters as it informs the patient and is a "safety net" for ensuring follow up plans are actioned.		HP gave letter high quality score of "9/9" and 9s in all other areas including GP care management plan except HP gave letter "4/9" for patient comprehensibility. HP concern that patients receiving letters may cause anxiety and distress. HP answered that it would be more appropriate for patients to receive personalised letters.	Although letter graded successful, GP did identify issues. Letter given a top score of "9" by HP. GP and HP appear to have differing views on whether patients should receive copies of their discharge letters with HP expressing concern and GP focussing on benefits.
2	S		Patient generally pleased with discharge experience and happy to have received copy of the letter. Patient likes to be informed. Patient suggests some issues with understanding medical terminology and says that they would prefer to receive patient personalised letter. Patient would prefer choice of receiving letter at discharge.	HP gave overall quality score of "7/9" and patient comprehensibility score of "9/9". HP reports to always copy patients into letters and believes patients should have choice of receiving letters. Answers that patients should receive GP copy of discharge letter.	HP and patient agree about patients receiving letters but appear to disagree over the form that this should take – patient favours personalised correspondence whereas HP favours patients receiving copies of what is sent to the GP.

For peer review only - http://bmjopen.bmj.com/site/about/guidelines.xhtml

Trio case	GP grading	GP comment and interview/focus group findings	Patient interview findings	HP survey findings	Summary of main trio findings
3	S		Patient overall seems pleased with communication and adds that they were given written and verbal information but only as they asked for a copy of the written information and that this was obtained after discharge. Patient describes follow up information in letter is unclear. Patient happy to receive copy of what GP receives and thinks it is reassuring to view the correspondence between doctors for transparency. Patient would prefer more detailed explanations in letter.	HP gives quality score of “8/9” with patient comprehensibility score of “9/9”. Answers that patients should receive personalised letters and that patients should be given a choice. HP reports that despite hospital policy and their views on patient choice, they have never given a patient a discharge letter copy. HP believes that part of discharge letter should be given to patient and this is what is meant by personalised, not for two summaries to be generated.	HP given letter top score for patient comprehensibility but patient does report some issues and possible improvements which could be made to letter. Patient and HP in agreement over patient choice of receiving letters but disagreement over form.
4	S		Patient says they were impressed with information provided; they were given a discharge letter copy. Patient thinks patients should receive letters automatically.	HP gave overall letter score of “9/9” and patient comprehensibility score of “9/9”. HP reports to give patients letters most of the time and thinks patients should receive GP copy in opt out style system.	Broad agreement between HP and patient within this trio case.
5	US	Unclear procedure due to acronyms not comprehensible to GP; for this reason, unclear what had been done. GP thinks abbreviations should be written out in full for clarity both for the sake of the patient and themselves.	Patient received letter after long discharge delay in hospital. Patient pleased to have received letter. Patient says they cannot understand all of letter but that they are aware they can ask the GP if they want to understand more.		Patient assumes GP understands all of letter and is a source of information for interpretation when GP does not due to use of uncommon abbreviations in letter.

For peer review only - http://bmjopen.bmj.com/site/about/guidelines.xhtml

Trio case	GP grading	GP comment and interview/focus group findings	Patient interview findings	HP survey findings	Summary of main trio findings
6	S	GP considered letter successful as follow up arranged. GP perceives use of acronyms in letter probably not comprehensible to patient. GP thinks use of lay terms in letter may be useful for patient understanding.	Patient thinks letter should ideally be emailed. Patient reports not being given much information and only received letter as relative went to hospital to get a copy after discharge. Patient feels discharge is not always clear and more time needs to be put in to ensure patient understanding. Patient felt letter generally inadequate and unsure of some of medical terms and acronyms in letter, patient states acronyms should not be used and terminology should be explained in lay terms.		GP and patient in agreement that letter format not entirely accessible to patient. Agreement over ways to rectify this issue through avoidance of acronyms and explanations of medical terminology in lay terms.
7	S	Letter graded successful as follow up clear. GP perceives letter written in patient friendly language.	Patient reports no difficulties with letter understanding but does note inaccuracies in letter.		GP and patient appear to agree on patient understanding.
8	US	Letter graded unsuccessful as drug changes and reasons for these unclear.	Patient reports being very pleased to have received copy of discharge letter having been given limited information in regard to previous discharges. Patient felt receiving letter supported their wellbeing. Patient conveys that receiving letter means that they can be actively involved in their own care and thus increase patient autonomy.		Patients receiving letters may support and improve patient wellbeing.
9	S	GP graded letter successful as it gave full details of investigations and findings and a working diagnosis. Important in GP view for patient to be given plan of action and instructions.	Patient reports not to have been given a copy of the letter. Patient would have preferred to have been given written information to ensure that they do not forget anything.		Patient and GP in agreement that patient did not receive a Letter and both appear to support practice of patients receiving letters.

For peer review only

Trio case	GP grading	GP comment and interview/focus group findings	Patient interview findings	HP survey findings	Summary of main trio findings
10	S	Letter graded successful as clear notes. Generally, letter informative and clear. GP raises possible issues with patient understanding due to presence of jargon and abbreviations; GP notes some patients would be fine with not understanding these elements whereas some patients will want to know more and may bring letter to GP with queries. GP says that there is a certain amount of technical information that needs to be passed between doctors but to improve patient understanding the letter should be clear and concise with use of lay language.	Patient given a copy of the letter. Patient reports medication information is very useful and clear but notes some issues with abbreviations for which they suggest an abbreviation chart. Patient suggests use of lay terms to make information clearer. Patient says receiving letter decreases the need to see the GP post-discharge.		GP and patient agreed that unexplained abbreviations may not be clear to patient and in order to increase patient understanding, acronyms and abbreviations should be spelt out in full and jargon should be accompanied by lay explanations.
11	S	Letter graded successful as detailed and clear plan. GP did note actions for patient and what the patient told unclear.	Received discharge letter. Patient suggestion that medical terminology could be better explained for patient. Suggestion that verbal explanatory information should accompany letter.		Patient felt in order to increase their understanding, jargon should be accompanied by lay explanations.
12	S	Letter graded successful as clear medication information and plan. Generally, GP happy with letter but is not sure how understandable this letter would be to patient. GP feels clinical summary and medication information would be useful to patient and that it is useful for patient to have a copy of the letter.	Patient received letter. Patient found letter information adequate and found medication information particularly useful. Patient felt information and detail in the letter was perhaps excessive and could be shortened and simplified.		GP and patient in agreement that discharge letter can usefully provide up to date medication information for patient. Patient felt letter contents could be simplified to increase its usefulness to them.

Trio case	GP grading	GP comment and interview/focus group findings	Patient interview findings	HP survey findings	Summary of main trio findings
13	S	Letter graded successful as clear medication information and follow up arranged. GP felt it was useful that letter says drugs started and stopped and reasons why. GP felt instructions to patient and follow up very clear. GP feels letter is appropriate and likely to be useful and comprehensible to patient.	Patient showed preference for receiving copies and did receive a copy in this case which they found useful. Patient liked that letter was simple and comprehensive but also brief. Suggestion that letter could be emailed to accelerate process.		GP and patient in agreement about letter usefulness and comprehensibility to patient.
14	S	Letter graded successful due to level of detail. GP reported issues with hospitals presuming GPs have access to system to view results when they often do not. Although GP graded letter successful, GP did comment that the letter would benefit from more information regarding the clinical summary and admission details. GP assesses letter as appropriate for patient.	Patient given discharge letter from hospital. Patient happy with this information, they felt it was clear what was wrong, what was going to happen next and medication information. Patient reports no problems with reading or understanding letter. Patient feels letter could have more detail. Patient thinks letter system should be opt out and patients should ideally receive personalised letters. Patient suggests use of lay terms to increase letter usefulness.		GP and patient in agreement about letter usefulness and comprehensibility to patient as well as level of detail for letter to be useful. Patient suggests use of lay terms to increase usefulness of letter to patient.
15	US	GP reports issues with the fact that the doctor writing the letter has not seen the patient. GP actions in letter described as ambiguous and inaccuracies noted by GP. The GP felt generally the letter is appropriate for the patient but raises concerns that the vague and unclear parts of the letter may cause patient anxiety. GP suggests how letter could meet needs of both GP and patient through simple interpretations of results and brief summarising of technical information to include breakdown of acronyms. GP felt unexplained acronyms should be avoided for the sake of patient understanding.	Patient not received letter and felt discharge communication process was poor. On letter review, patient was unclear on some of the medical terms in letter. Patient would have preferred to have been given copy of letter by hospital. Patient felt written discharge correspondence to patients should be mandatory.		GP suggests use of lay terms and simple interpretations to increase usefulness of letter to patient. Patient felt patient correspondence after discharge should be mandatory. GP felt acronyms should be avoided for the sake of understanding and clarity for patient. GP and patient in agreement that discharge communication unsuccessful.

For peer review only

Trio case	GP grading	GP comment and interview/focus group findings	Patient interview findings	HP survey findings	Summary of main trio findings
16	S	GP commented that patient not given a copy and they felt that the patient should have and that the letter would have been entirely appropriate for the patient. GP feels letter may have been reassuring for patient. GP comments that sharing letters with patients is the gold standard. Discharge plan simple and letter successful as concise and clear.	Patient reports being copied into recent letters but has found some of the letter contents technical. Despite this patient would prefer to receive copies of the letter sent to the GP rather than a patient personalised letter. Patient feels happy when they receive letters.		GP preference and patient preference for patients receiving letters. GP and patient disparity about whether or not patient received a copy of their recent discharge letter.
17	US	Letter graded unsuccessful as limited information regarding medication and investigations. GP found medication information unclear as well as working diagnosis. GP unsure whether or not letter wording would cause patient anxiety due to the diagnosis sounding serious. GP unsure whether letter language comprehensible to patient as many technical medical terms. GP thinks for safety netting, it is useful for the patient to know what the follow up plans are. GP reports information given to patients seems variable.	Patient says they were given discharge letter but with no accompanying verbal information or opportunity to ask questions. Patient reports feeling disappointed with discharge communication. Patient feels letter is not entirely accurate and that there have been ramifications as a result of this. Patient saw serious diagnosis for first time in letter which was slightly worrying.		GP and patient seem to be in agreement that discharge communication unsuccessful and that it is not ideal for the patient to be finding out about a potentially serious diagnosis for the first time in a letter with no accompanying counselling.
18	S	GP thinks patients need to know what is happening via a simple letter in lay language. Letter has handwritten pencil annotations which are unclear. Letter graded as successful due to good clinical summary and clear GP actions. GP concerns that receiving this letter may make patient feel anxious. GP raised issues with current prevalence of inaccuracies in discharge letters.	Patient says that they like to receive letters as they like to know what is going on. Patient feels discharge communication is good as long as they get a copy of the discharge letter.		GP and patient do not seem to be in agreement about patient appropriateness of letter. GP perceives letter may cause patient anxiety when the patient did not report this.

For peer review only - http://bmjopen.bmj.com/site/about/guidelines.xhtml

Trio case	GP grading	GP comment and interview/focus group findings	Patient interview findings	HP survey findings	Summary of main trio findings
19	S	Letter graded successful as clear diagnosis, summary, medication, diagnosis and plan. Nothing missing from the letter in GP view. To make letter clearer to patient, GP suggests jargon could be broken down and explained.	Patient happy to have received something written down so that they did not have to remember it. Patient mentions jargon not all initially clear but also says terms can be easily looked up on the internet or through other means. Patient likes to receive the same information as their GP.		GP concerned that patient may not understand letter and that letters such as this may need explaining. Patient happy to have received letter and notes resources such as internet that can be used to look up unknown terms.
20	US	Letter graded unsuccessful due to lack of medication details. Letter appropriate for patient only if they had knowledge of the information previously. GP thinks it is OK for patients to get copies as long as the letter is clear and meaningful to the patient otherwise the GP will need to spend time explaining letters to patients.	Patient seems somewhat indifferent to receiving letters and is most concerned that a copy is received by the GP. Patient would like to be given choice about receiving letter despite feeling that they often do not need a copy. Patient notes no faults with the letter.		Patient and GP disagree about quality of letter.
21	S	GP comments that letter is good quality and sufficiently detailed. GP feels generally letters are appropriate for patients and that it is useful for patients to have record of treatment and medications.	Patient values receiving letters and can understand them and finds them comprehensible. Broadly, patient impressed with letters they have received including the most recent.		GP and patient in agreement that letter suitable and useful for patient.
22	US	GP feels letter contains limited detail and no results of investigations or information regarding treatment. Due to lack of information, letter requires GP follow up to clarify details. GP unsure if this letter would be useful to a patient due to the lack of detail.	Patient pleased to have received copy of the discharge letter. Patient found letter very helpful. Patient prefers to receive copy of what is sent to the GP and unsure why anyone would want anything different. Patient cannot see way to improve letter.		GP and patient disagree on quality of letter.

For peer review only - http://bmjopen.bmj.com/site/about/guidelines.xhtml

Trio case	GP grading	GP comment and interview/focus group findings	Patient interview findings	HP survey findings	Summary of main trio findings
23	US	Letter grading due to the fact that the letter does not make sense to GP and is generally inadequate.	Patient likes receiving letters and to know what is going on. Patient reported no problems with letter or receiving it. Patient likes to receive a copy the same as what the GP receives.		GP and patient disagree on letter quality.
24	S	GP cannot think of case where it would not be appropriate for the patient to have a copy of the letter. GP believes patients receiving letters promotes and encourages autonomy and patient informed-ness and can also be reassuring. GP feels overall letter is clear and succinct.	Patient notes verbal and written information was conflicting. Patient pleased to have received letter and felt it was informative. Patent thinks patients need to know what happened, medication information and follow up plan. Patient feels letter system should be opt out to reduce the risk of patients mistakably not receiving letters.		GP and patient seem to agree on the benefits of patients receiving letters – that it can inform on condition and what is next.
25	S	GP expresses concerns with patients comprehending medical terms in discharge letters. GP does add that often patients having letters is useful particularly for GP home visits. GP expounds difficulty writing a letter to meet the needs of two audiences – GP and patient.	Patient reports being given limited information at the time of discharge. Patient notes a few inaccuracies on letter which made them feel uneasy about the rest of the letter and its accuracy, content, and quality. Broadly, patient did not feel the discharge experience was particularly good.		GP and patient slightly disagree on letter quality – GP grades as successful but patient does not describe communication and discharge experience positively.
26	S	GP graded letter successful as findings and plan clear. GP feels no new information should be communicated to the patient in the discharge letter. GP thinks that whether or not it is useful for patient to have a copy of the letter depends on the content and quality of letter. GP feels notes letters should never be handwritten as this can be unclear and thinks generally processes need improving to support better communication.	Patient reports being given limited information and no copy of the letter. Patient was left feeling slightly confused about what was going on. Patient would prefer to always receive copies of letter and for this to be the same as what the GP receives.		GP and patient in agreement that patient receiving letter can be useful.

For peer review only - http://bmjopen.bmj.com/site/about/guidelines.xhtml

Quartet meta-matrix with narrative summaries (*US=unsuccessful, S=successful)

Quartet case	GP grading*	GP comment and interview/focus group findings	Patient interview findings	HP survey findings	Main quartet findings
A	US	Letter graded unsuccessful by GP as diagnosis and reason for admission unclear as blank on letter template. GP unclear of cause of patient symptoms and presenting complaint and whether this cause is known to hospital. GP raises possible issues with patient understanding due to presence of jargon and abbreviations. GP thinks avoiding acronyms and use of lay terms in letter may be useful for patient understanding and notes that letter should be provided within context of adequate patient counselling. GP suggests patient information section on letter. GP feels template letters are good as they avoid things being missed. GP likes to know diagnosis, admission and discharge date, consultant details, medication, procedures and results, and patient awareness of diagnosis. GP feels blanks on summaries should not be permitted as unclear.	Patient received copy of letter but did not seem too pleased as they noticed inaccuracies on the letter which made them feel upset/angry. However, patient does find it useful to receive letter so that they can remedy discrepancies. Patient feels someone should go through letter with patients prior to discharge to reduce inaccuracies and ensure patient understanding. Patient prefers to receive direct copy of GP letter. Patient feels letter should have contained name of discharging physician.	HP gave overall letter a quality score of “6/9” with diagnosis information as “2/9” and patient comprehensibility as “2/9”. HP felt patients should have a choice about receiving letters and that they should receive a GP copy. HP notes issues with letters being completed by most junior doctors, some of whom may not be on the corresponding consultant speciality team leading to issues. The HP comments that they tend to dictate letters which allows more information to be inputted as the template can be limiting.	Apparent agreement across all three groups that letter is somewhat unsuccessful. All groups raise issues with letter accuracy and HP notes this is likely due to junior status of completing doctor. GP and HP seem to agree patients should receive letter and patient agrees with this noting that had they not received the letter; they would not have been able to rectify the errors. Patient and GP agree that letter should be provided within the context of patient counselling.
B	US	GP comments that they have no way of knowing whether or not patient received letter. GP feels letter is not patient appropriate and could cause patient to feel anxious due to amount of medical language. GP adds that to improve letter, lay language for patient could be used. GP comments that it is good there are no handwritten sections on letter and that the findings are clear. GP feels patients need to know the procedure and results and follow up. GP comments that it is useful when patients receive letters because it helps them understand the action plan. GP feels that discharge letters need improving in terms of timeliness, factual accuracy, details regarding	Patient been given a copy of letter; it was in an unsealed envelope so they read it. Patient notes that follow up stated on letter has not happened. Patient notes they were lucky to have someone with them in hospital who remembered information as they did not due to effects of anaesthesia. Patient would have preferred interpretative simple summary of results. Patient mentions importance of considerations of the individual	HP gave overall quality score of “5/9” with patient comprehensibility score of “7/9”. HP felt patients should receive choice of receiving letters and that this should be a GP copy. HP notes that they do not always have much time to complete discharge summaries and so must be brief. HP notes completing summaries which are timely but also informative and accurate is very challenging.	GP concerned that patient may not understand letter and that letters such as this may need explaining. Patient happy to have received letter and notes resources such as internet that can be used to look up unknown terms. Lower quality of letter perhaps explained by HP comments regarding the time pressures of completing summaries in their role.

For peer review only - http://bmjopen.bmj.com/site/about/guidelines.xhtml

Quartet case	GP grading*	GP comment and interview/focus group findings	Patient interview findings	HP survey findings	Main quartet findings
		what has happened, and plan of action. GP says that GPs are not responsible for chasing results and yet letters request this of them.	and patient choice. Patient notes that unfamiliar terms can easily be searched on internet.	HP notes that they feel their discharge letters are generally adequate but some HPs include only brief details.	
C	S	Successful grading as all information clear and concise including diagnosis and treatment plan. GP feels unexplained acronyms should be minimised for clarity for both GP and patient. GP notes inconsistency of patients receiving letters. GP raises concerns with patient understanding letter due to acronyms, one of which the GP is unfamiliar with, and medical terminology. GP feels that letter should clearly summarise the results in patient-friendly language to make content clearer (e.g. it should be stated that test results were normal for reassurance). GP feels the important items for letters are diagnosis, reason for admission, clinical summary, treatment and results, medication, and follow up and GP actions. GP feels letters are currently very variable in terms of quality. GP thinks patients should only not be given letters in cases of harm. GP comments that the "blank" GP action on letter is confusing and if there is no action this should be explicitly stated for clarity.	Patient has letter and notes that this is useful so if they go abroad they could show the letter to any clinicians looking after them as relevant. Patient notes that different patients may want different levels of information particularly in regard to bad news. Patient reports that they understand letter and are happy with it although they would have preferred to have been given a copy of the letter through the hospital rather than because they took part in the research. Patient suggests letter could be improved by being written in plain English. Patient notes the importance of adequate patient counselling. Patient values knowing next steps.	HP gives letter quality score of "8/9" across all categories to include patient comprehensibility. HP thinks patients should receive a choice of receipt and that the form should be personalised letters. HP rates their letters highly but adds no comments as to why.	GP expresses concerns regarding the patient understanding letter but patient notes that they did understand the contents. However, the GP and patient agree that the letter would be more useful if it was written in plain English with minimal or no acronyms. The HP seems unaware of the acronym issues. The HP feels patients would benefit from personalised letters but patient says they have preference for receiving a copy of what the GP receives. Letter seems to be evaluated as successful across population groups.
D	S	GP thinks patients receive letters variably. GP notes that language in letters is often very medical and so not suitable for the patient without explanation. The GP asserts that letters can be written in a straightforward way for the patient. GP feels patients should receive letters and says this can make patients feel more included in their care. GP feels letter is a bit brief in regard to results and follow up. Good elements of the letter are that tests have been overviewed. The GP feels a summary of the results to include interpretations	Patient says they did not receive a copy of the discharge letter but they would have liked one had it been offered. Patient would have preferred results to have been clearer and letter to make use of lay terms. Patient would like to be given letter every time they attend hospital. Patient suggests letter could be improved by clearer summary of	Letter given "1/9" by HP across quality scores. HP comments that the letter is poor because it was generated by a computer and was not written by themselves.. HP writes that the computer is unable to select the salient information and communicate it and so sometimes they send	HP and GP seem to agree that computerised templates are not particularly helpful. Groups broadly agree about letter quality. All groups agree patients should receive letters.

For peer review only - http://bmjopen.bmj.com/site/about/guidelines.xhtml

Quartet case	GP grading*	GP comment and interview/focus group findings	Patient interview findings	HP survey findings	Main quartet findings
		would be useful for the patient and the GP. The GP makes a general comment on the dangers of rapid hospital requests post-discharge.	what happened, medication, treatment, and follow up plans.	a separate letter to the GP with the important information.	
E	S	Letter graded successful as reason for admission and follow up plan were clear as were actions for GP and medication changes. GP favours that GP action in letter not blank but clear that the GP does not need to undertake further actions. GP feels the letter would be appropriate and useful to patient but may be improved by use of lay terms. GP notes patients receive letters inconsistently but they think it is useful for patients to receive copies particularly in regard to medication information. GP notes difficulty of writing letter that is patient friendly whilst meeting technical needs of GP. GP feels information in letter is quite medical and may be confusing/concerning for a patient; GP suggests lay explanations would help. However, GP does note letter would likely be useful for the patient so they are aware of the follow up plan. GP thinks important elements for letters are tests and results, diagnosis, GP action points. GP suggests patients are given abbreviated copies to include diagnosis, medications, and follow ups.	Patient reports that they had not received copy of letter but they would have liked to have done despite that the letter communicated bad news and a serious diagnosis. Patient would prefer copy of what goes to the GP and that this is useful so they can refer back to it so they are not dependent upon remembering information. Patient would like information in the letter relating to what happened and next steps.	HP rates letter “8” in all quality categories including GP information and patient comprehensibility. The HP notes producing summaries on a weekend when they are understaffed is a barrier to producing high quality letters. The HP feels their letter is clear and informative. The HP comments that the [hospital B] discharge templates are superior to the [hospital A] ones as they allow more freedom with inputting information.	The HP reports they always copy patients into letters and yet the patient reported they had not received a copy of the letter. There seems to be agreement across the groups that the letter was successful. GP expresses concern about patient understanding due to medical terms. The patient noted no understanding issues and found the letter useful.
F	US	Letter graded unsuccessful as unclear diagnosis and medication information. GP suggests that letter could be improved by medication information being put at the end of the letter rather than the beginning as this may cloud other important information. GP comments that positive aspects of the letter such as the inclusion of investigations, management plan, and actions for GP. Another letter improvement would be to specify if any blood tests need repeating and if so which ones and when. GP feels patients should receive letters.	Patient reports that they had received a copy of the discharge letter although one page missing when compared with GP copy. Patient found the medication information unclear. Patient also felt the diagnosis information was unclear and that they were given conflicting verbal and written information. The patient comments that they would like to receive a discharge letter every	HP grades letter an “8/9” for overall quality. HP notes restrictive template of summary can be a barrier to providing detail. The HP comments that upon reviewing the diagnosis it is unclear and they should/could have explained the presenting complaint better. The HP comments on the frustration that reports cannot be cut and pasted into the summary and	GP and patient seem to agree that letter requires improvements and that the medication information is unclear. All agree diagnosis information is unclear.

Quartet case	GP grading*	GP comment and interview/focus group findings	Patient interview findings	HP survey findings	Main quartet findings
			time they are discharged from hospital.	that the templates have restricting word counts.	
G	S	Discharge letter successful as it was concise with clear reason for admission, treatment, follow up, information given to patient, investigations and results. GP values that the medication changes in the letter are clear which is useful. GP thinks patients should receive letters but notes issues with jargon. GP feels current quality of discharge letters is variable and many letters have incomplete medication lists and insufficient detail regarding tests carried out and GP actions.	Patient reports being given copy of letter which they were happy with. Patient notes communication difficulties of being transferred between care providers. Patient felt medication information was a bit unclear and that when they were discharged, they still did not know the cause of their condition.	HP gives quality score of "6/9" and patient comprehensibility score of "3/9". HP thinks patients should receive GP copies but not always. The HP comments that their spelling and grammar let them down but they do feel the management plan and diagnosis in the letter are succinct and informative.	Agreement between GP and patient as letter contained clear follow up and diagnosis but HP rates letter quality lower due their spelling and grammar mistakes.
H	US	Letter graded unsuccessful as no diagnosis and medication list incomplete. GP does note that there is a follow up plan which is helpful but without the diagnosis the letter is not clear enough. GP notes this letter does not contain enough detail. GP feels patients should receive letters but raises issues with unexplained medical terms. GP feels it is useful for patients to have record of medication and treatment. GP feels patient understanding could be improved through adequate patient counselling regarding discharge letter information.	Patient felt unclear of what the problem was when they discharged due to little information received. Patient reports that they did not receive a copy of the discharge letter but they would have liked to have done. Patient suggests that a patient personalised letter may be more valuable but that they would want both letters. Patient mentions use of internet for looking up unknown terms.	HP gives letter a "6/9" for quality and patient comprehensibility but rates diagnosis information a "2/9" as on reflection they feel this is unclear. The HP thinks the follow up information is also poor. HP thinks patients should receive GP copies and always be given a choice of receipt. The HP feels the letter could have been improved by specifying the differential diagnoses in light of the presenting complaint.	Diagnosis information indicated as unsuccessful across all three groups. GP raises issues with patients understanding medical terms. Patient mentioned no issues with letter contents and said that terms can easily be internet searched.
I	S	Successful grading as clear, inclusive of relevant information, and explained what information and advice given to the patient which the GP reports is not always included on summaries but very important. GP suggests issues with patients understanding letters particularly regarding medication changes and feels letters need to be written in plain English and lay language with	Patient reports to be given verbal information only and no letter which they did not find helpful. They would like to receive letters to include more detailed management and recommendations information. Patient wants letter to contain	HP gives scores of "9/9" for all categories except patient comprehensibility which they give "7/9". HP claims to always copy patients into letters. HP commented that the letter was successful.	GP feels abbreviations need to be avoided in letters as these are not patient friendly. Patient and GP agreed that letter should be written in plain English with explained terms. GP and patient agree that patient actions and

For peer review only - http://bmjopen.bmj.com/site/about/guidelines.xhtml

Quartet case	GP grading*	GP comment and interview/focus group findings	Patient interview findings	HP survey findings	Main quartet findings
		minimal or no abbreviations. GP feels patients receiving letters is a good idea but needs to be accompanied by adequate patient counselling and letters should clearly highlight if the patient is required to take any action. GP notes that a successful letter is not a long letter.	specific information about what is wrong, medication, and how condition can be improved. Patient feels receiving verbal & written information is useful.		recommendations need to be explicit and clearer in the letter.
J	US	Unsuccessful grading due to lack of clear findings and follow up plan. GP feels the letter should have included clear details of the discharging physician and also information given to the patient alongside presentation of clinical findings. GP comments that the letter is particularly unclear as it is handwritten and illegible and so they feel uncertain of the exact procedure that the patient has had and the outcome. GP feels that this specific letter would not be helpful to the patient as it contains no information or advice or follow up details. GP also comments that the letter contains too many medical terms which would be hard for the patient to understand. GP notes general usefulness of patients receiving copies but says the letter should accompany counselling. The GP feels letters should always be typed.	Patient reports difficulties remembering the verbal information they were given as no letter. Patient was given a letter for the GP but as it was in a sealed envelope, they did not open it. Patient suggests they should have been given advice for condition and management, details of any follow up and medications, and expectations of recovery. Patient would prefer to receive a direct copy of what is sent to the GP and thinks patients should always be given letters as information can be easily forgotten.	HP gives letter quality score of "2/9" and notes it was actually produced by someone else more junior on their team but the letter has their name on. The HP rated the letter poorly across quality scales but did not provide any details as to how the letter could have been improved.	GP feels nothing in this particular letter would be of use to patient. Patient had trouble remembering the verbal information. Agreement across all three groups that discharge communication poor and unsuccessful. GP notes the illegibility of the letter due to handwritten form. The patient and HP focus on the content brevity. GP and patient agree that patient needs to know advice and follow up plans.

For peer review only

Table of Developed CMOCs (context, mechanism, outcomes configurations)

CMOC	Context	Mechanism	Outcome	Effect assessment	Does it "work" or not?
CMOC1	patient not offered letter	patient feels less involved in care	reduced patient autonomy	negative	does not work
CMOC2	patient offered opportunity to receive letter(s)/patient choice respected	patient feels more informed and involved in care	increased patient autonomy and increased involvement of patients in treatment, care and communications	positive	does work
CMOC3	large clear posters displaying patients right to choose and importance of correct contact information	patient realises they should inform hospital of address changes and preferences	lowered risk of confidentiality breach	positive	does work
CMOC4	NHS drive for patient-led care (influence or context)	clinicians increasingly offering patient choice of receiving letter/sharing information with patients	increased patient empowerment	positive	does work
CMOC5	clinician views letters to patients are beneficial e.g. increases transparency, compliance, trust, patient satisfaction, patient understanding and recall	clinician feels patient should be offered letter	potential increase in patient autonomy & satisfaction	positive	does work
CMOC6	Clinicians views letters to patients as not beneficial e.g. letter not comprehensible to patient, medico-legal issues, increased cost and staff workload, patient harm	clinician feels patient should not be offered letter	no patient autonomy	N/A	unclear

For peer review only

CMOC	Context	Mechanism	Outcome	Effect assessment	Does it "work" or not?
	(anxiety, distress, and confusion) and issues around confidentiality				
CMOC7	NHS guidance that all hospital-GP correspondence should be copied to patient as a "right" where appropriate and if patients agree (unless risk of serious harm or legal issues)	clinicians increasingly offering patient choice of receiving letter	increased use of NHS resources to send letters but patient benefits through increased understanding & potential reduction in patient queries (costs balanced)	positive	does work
CMOC8	Data Protection Act 1998 (UK)	Patients may become aware of their right to know what is written & stored about them	Patients informed of their stored electronic information (increased transparency)	positive	does work
CMOC9	doctor copies letters to patient	patient trusts doctor more	improved doctor-patient relationship	positive	does work
CMOC10	patient offered choice of receiving letters	patient chooses to receive letters	Increased administrative staff workload and costs of printing & posting letters	negative	unclear
CMOC11	patient offered choice of receiving letters	patient chooses to receive letters	reduced queries and GP visits and reduced hospital re-admissions (limited evidence)	positive	does work
CMOC12	structured discharge letters written clearly in plain English (pref. 5th grade level) with medical jargon explained with lay terms, no value judgements of patients and minimal abbreviations	patients understand letter	increased patient knowledge	positive	does work

CMOC	Context	Mechanism	Outcome	Effect assessment	Does it "work" or not?
CMOC13	doctors provided training in letter writing & record keeping (contextual influence) leading to doctors write letters of higher quality and more appropriate for patients	patients understand letter	Increased patient knowledge/potential increase in doctor confidence in letter writing	positive	does work
CMOC14	patient preference for letter copies acknowledged and patient offered choice of receiving letter	patients feel able to express their preference	decreased strain on resources & increased patient autonomy & satisfaction	positive	does work
CMOC15	patient provided written & verbal information to include sufficient counselling	patient reflects on written record of information for reference	increased patient knowledge of care plan, recall and acceptance of illness or condition	positive	does work
CMOC16	Human Rights Act (1998) and Race Revelations Act (2000) - clinicians equally offer all patients letter copies regardless of background	clinician feels all patients should be offered letter	increased equality and accessibility of information to patients	positive	does work
CMOC17	Use of pictures/pictographs/cartoons with written information	patients understand letter	Patient benefits from improved understanding e.g. adherence to agreed care plan	positive	does work
CMOC18	verbal information only	patient may not be able to retain information	reduced patient recall	negative	does not work
CMOC19	professionals who are not involved/limited involvement with patient writes letter	professional does not understand patient plan	letter quality reduced/increased risk of harm	negative	does not work
CMOC20	patient hospital visit of sensitive nature and/or patient lacks capacity e.g. psychotic episode, dementia	patient finds letter distressing and/or confusing	harm to patient	negative	does not work

For peer review only

CMOC	Context	Mechanism	Outcome	Effect assessment	Does it "work" or not?
CMOC21	Patient letter written above patient educational level or in a language the patient does not read	patient finds letter difficult to understand	patient is confused with no increased knowledge of care/possible misinterpretation of care instructions	negative	does not work
CMOC22	letter contains inaccurate information	patient identifies inaccuracies	patient notifies hospital/GP of inaccuracies and corrections are made leading to improved record keeping	positive	does work
CMOC23	patient receives discharge letter	patient does not understand entirety of letter	patient sources answers (internet, GP, friend or relative)	positive	does work
CMOC24	Patient specific letter sent to patient	patient finds letter clear	improved patient comprehension	positive	does work
CMOC25	Patient specific letter sent to patient	Clinician produces two letters	increased staff workload and costs	negative	does not work
CMOC26	Patient specific letter sent to patient	Patient identifies information sent to GP and patient is different	medico-legal concerns could be raised over letter discrepancies and any withheld information	negative	does not work
CMOC27	hospital sends patient discharge letter without verifying patient contact details without notifying patient	hospital worker does not identify and correct incorrect information	potential breach of patient confidentiality	negative	does not work
CMOC28	hospital routinely checks patient addresses and sends discharge letters to patient marked confidential using full name	hospital worker identifies and corrects incorrect information	patient receives letter, minimal risk of patient confidentiality breach	positive	does work
CMOC29	patient receives discharge letter	patient may feel they have questions relating to letter	patient contacts health provider with queries (evidence suggests minimal impact and queries)	positive	unclear

For peer review only

CMOC	Context	Mechanism	Outcome	Effect assessment	Does it "work" or not?
CMOC30	discharge letter/summary dictated in front of patient	patient queries any inaccuracies	letter less likely to contain inaccuracies	positive	does work
CMOC31	Hospital gives patient letter to deliver to GP	patient may find they are unable to make delivery or patient does not like being asked to perform this task	GP does not always receive letter. Patient satisfaction low.	negative	does not work
CMOC32	Patient receives letter not written at appropriate level for them	patient does not understand letter	patient feels confused and dissatisfied with discharge care	negative	does not work
CMOC33	Patient has anxiety that doctors talk about things behind their backs	patient who receives letter feels reassured that there is no hidden information	decreased patient anxiety and improved doctor-patient relationship through transparency	positive	does work
CMOC34	patient receives discharge letter	Patient feels they are important to clinician	patient is impressed with letter and feels clinician has an interest	positive	does work
CMOC35	choice about whether letter is sent to patient	clinician feels letters would be a disaster and inappropriate for patient	patient does not receive letter(s)	N/A	unclear
CMOC36	patient receives discharge letter	Patient feels indifferent	no impact on patient	N/A	unclear
CMOC37	patient receives discharge letter with bad news	Patient finds letter initially distressing	letter causes initial distress but final outcome that patient finds letter helpful and aids recall and acceptance of condition	positive	does work
CMOC38	letter sent to patient containing information not discussed with patient or abnormal results	patient feels distressed and anxious reading letter	patient harm/unethical practice	negative	does not work
CMOC39	patient worried about diagnosis and receives letter	patient understanding helped by letter	patient feels less anxious due to being more informed	positive	does work

For peer review only

CMOC	Context	Mechanism	Outcome	Effect assessment	Does it "work" or not?
CMOC40	patient preference for letter copies not acknowledged	Patient dissatisfied to have received letter	decreased patient satisfaction	negative	does not work
CMOC41	patient offered choice of receiving letters (opt out)	patient enabled to decide on letter preference	patient may or may not receive letter depending on their preference in relation to the particular care episode resulting in higher patient satisfaction. Increased rate of patients receiving letters	positive	does work
CMOC42	patient who feels copies of letters are not necessary for themselves	Patient pleased not to be given letter	patient satisfied, secondary outcomes: costs and time saved	positive	does work
CMOC43	patient receives discharge letter where appropriate	patient understands letter	patient finds letter informative and helpful. Patient wellbeing boosted and supported	positive	does work
CMOC44	patient receives discharge letter where appropriate	patient feels involved in care plan	patient ensures follow up plan is followed and books any necessary tests etc.	positive	does work
CMOC45	patient receives discharge letter where appropriate	patient feels letter is important	letter forms permanent record of hospital visit and kept for future reference. Patient may show letter to family and friends.	positive	does work
CMOC46	patient receives discharge letter for breaking good news	patient reminded of discussion	patient feels reassured and has "peace of mind"	positive	does work
CMOC47	patient receives discharge letter where appropriate (patient choice)	patient likes receiving letter	patient satisfaction increased	positive	does work
CMOC48	patient receives copy of discharge letter where appropriate	patient becomes aware of what GP knows	Patient reassured that GP knows about visit	positive	does work

For peer review only

CMOC	Context	Mechanism	Outcome	Effect assessment	Does it "work" or not?
CMOC49	Tickbox/template allows letter copies to patients to be monitored and audit trailed	HP becomes aware of practice of copying patients letters	Increased practice of patients receiving letters. Inconsistencies can be monitored for improving uptake.	positive	Does work
CMOC50	Letter acts as record of consultation and given to patient	Patient reminded of consultation	Patient recall increased and no need for patient to remember all consultation information	positive	Does work
CMOC51	Letter acts as record of consultation and given to patient	Patient prompted to use letter for administrative proceedings without need to contact GP or hospital	Letters can be used as proof of illness for benefit receipt, government support, disability applications and allowances, or time off work.	positive	Does work
CMOC52	Patient episode of care due to repeat or ongoing condition	Patient feels already informed about condition	Patient chooses not to receive letter preserving resources	positive	Does work
CMOC53	Patient receives letter with irrelevant or poorly phrased social disease or behaviour details	Patient feels judged and upset	Patient reflects on episode of care poorly and wellbeing negatively impacted	negative	Does not work
CMOC54	Letter provided to patient with additional patient information section	Patient understands summary	Patient knowledge increased and patient reassured that the important content points have been communicated.	positive	Does work
CMOC55	Clinician concern about patient understanding letter	Patient feels they do understand letter	Clinician concerns potentially unfounded. Patient values receiving letter	positive	Does work

For peer review only

COREQ (COnsolidated criteria for REporting Qualitative research) Checklist

A checklist of items that should be included in reports of qualitative research. You must report the page number in your manuscript where you consider each of the items listed in this checklist. If you have not included this information, either revise your manuscript accordingly before submitting or note N/A.

Topic	Item No.	Guide Questions/Description	Reported on Page No.
**Domain 1: Research team and reflexivity**			
Personal characteristics			
Interviewer/facilitator	1	Which author/s conducted the interview or focus group?	
Credentials	2	What were the researcher's credentials? E.g. PhD, MD	
Occupation	3	What was their occupation at the time of the study?	
Gender	4	Was the researcher male or female?	
Experience and training	5	What experience or training did the researcher have?	
Relationship with participants			
Relationship established	6	Was a relationship established prior to study commencement?	
Participant knowledge of the interviewer	7	What did the participants know about the researcher? e.g. personal goals, reasons for doing the research	
Interviewer characteristics	8	What characteristics were reported about the inter viewer/facilitator? e.g. Bias, assumptions, reasons and interests in the research topic	
**Domain 2: Study design**			
Theoretical framework			
Methodological orientation and Theory	9	What methodological orientation was stated to underpin the study? e.g. grounded theory, discourse analysis, ethnography, phenomenology, content analysis	
Participant selection			
Sampling	10	How were participants selected? e.g. purposive, convenience, consecutive, snowball	
Method of approach	11	How were participants approached? e.g. face-to-face, telephone, mail, email	
Sample size	12	How many participants were in the study?	
Non-participation	13	How many people refused to participate or dropped out? Reasons?	
Setting			
Setting of data collection	14	Where was the data collected? e.g. home, clinic, workplace	
Presence of non-participants	15	Was anyone else present besides the participants and researchers?	
Description of sample	16	What are the important characteristics of the sample? e.g. demographic data, date	
Data collection			
Interview guide	17	Were questions, prompts, guides provided by the authors? Was it pilot tested?	
Repeat interviews	18	Were repeat inter views carried out? If yes, how many?	
Audio/visual recording	19	Did the research use audio or visual recording to collect the data?	
Field notes	20	Were field notes made during and/or after the inter view or focus group?	
Duration	21	What was the duration of the inter views or focus group?	
Data saturation	22	Was data saturation discussed?	
Transcripts returned	23	Were transcripts returned to participants for comment and/or	

For peer review only

Topic	Item No.	Guide Questions/Description	Reported on Page No.
		correction?	
**Domain 3: analysis and findings**			
Data analysis			
Number of data coders	24	How many data coders coded the data?	
Description of the coding tree	25	Did authors provide a description of the coding tree?	
Derivation of themes	26	Were themes identified in advance or derived from the data?	
Software	27	What software, if applicable, was used to manage the data?	
Participant checking	28	Did participants provide feedback on the findings?	
Reporting			
Quotations presented	29	Were participant quotations presented to illustrate the themes/findings? Was each quotation identified? e.g. participant number	
Data and findings consistent	30	Was there consistency between the data presented and the findings?	
Clarity of major themes	31	Were major themes clearly presented in the findings?	
Clarity of minor themes	32	Is there a description of diverse cases or discussion of minor themes?	

Developed from: Tong A, Sainsbury P, Craig J. Consolidated criteria for reporting qualitative research (COREQ): a 32-item checklist for interviews and focus groups. *International Journal for Quality in Health Care*. 2007. Volume 19, Number 6: pp. 349 – 357

Once you have completed this checklist, please save a copy and upload it as part of your submission. DO NOT include this checklist as part of the main manuscript document. It must be uploaded as a separate file.

For peer review only

BMJ Open

The Discharge Communication Study: A Realist Evaluation of Discharge Communication Experiences of Patients, General Practitioners, and Hospital Practitioners, Alongside a Corresponding Discharge Letter Sample

Journal:	*BMJ Open*
Manuscript ID	bmjopen-2020-045465.R2
Article Type:	Original research
Date Submitted by the Author:	07-Jul-2021

Complete List of Authors:	Weetman, Katharine; University of Warwick Warwick Medical School, Unit of Academic Primary Care<br>Dale, Jeremy; University of Warwick Warwick Medical School, Unit of Academic Primary Care<br>Scott, Emma; University of Warwick Warwick Medical School, Unit of Academic Primary Care<br>Schnurr, Stephanie; University of Warwick, Applied Linguistics

**Primary Subject Heading**:	Health services research
Secondary Subject Heading:	General practice / Family practice, Health policy, Patient-centred medicine, Qualitative research, Communication
Keywords:	Health policy < HEALTH SERVICES ADMINISTRATION & MANAGEMENT, Protocols & guidelines < HEALTH SERVICES ADMINISTRATION & MANAGEMENT, INTERNAL MEDICINE, PRIMARY CARE, QUALITATIVE RESEARCH

SCHOLARONE™
Manuscripts

I, the Submitting Author has the right to grant and does grant on behalf of all authors of the Work (as defined in the below author licence), an exclusive licence and/or a non-exclusive licence for contributions from authors who are: i) UK Crown employees; ii) where BMJ has agreed a CC-BY licence shall apply, and/or iii) in accordance with the terms applicable for US Federal Government officers or employees acting as part of their official duties; on a worldwide, perpetual, irrevocable, royalty-free basis to BMJ Publishing Group Ltd ("BMJ") its licensees and where the relevant Journal is co-owned by BMJ to the co-owners of the Journal, to publish the Work in this journal and any other BMJ products and to exploit all rights, as set out in our licence.

The Submitting Author accepts and understands that any supply made under these terms is made by BMJ to the Submitting Author unless you are acting as an employee on behalf of your employer or a postgraduate student of an affiliated institution which is paying any applicable article publishing charge ("APC") for Open Access articles. Where the Submitting Author wishes to make the Work available on an Open Access basis (and intends to pay the relevant APC), the terms of reuse of such Open Access shall be governed by a Creative Commons licence – details of these licences and which Creative Commons licence will apply to this Work are set out in our licence referred to above.

Other than as permitted in any relevant BMJ Author's Self Archiving Policies, I confirm this Work has not been accepted for publication elsewhere, is not being considered for publication elsewhere and does not duplicate material already published. I confirm all authors consent to publication of this Work and authorise the granting of this licence.

For peer review only

The Discharge Communication Study: A Realist Evaluation of Discharge Communication Experiences of Patients, General Practitioners, and Hospital Practitioners, Alongside a Corresponding Discharge Letter Sample

Katharine Weetman[1], Jeremy Dale[2], Emma Scott[3], Stephanie Schnurr[4]

1. Unit of Academic Primary Care, Division of Health Sciences, Warwick Medical School, University of Warwick, Coventry, UK, CV4 7AL, **Early Career Fellow**
2. Unit of Academic Primary Care, Division of Health Sciences, Warwick Medical School, University of Warwick, Coventry, UK, CV4 7AL, **Professor**
3. Unit of Academic Primary Care, Division of Health Sciences, Warwick Medical School, University of Warwick, Coventry, UK, CV4 7AL, **Senior Research Fellow**
4. Applied Linguistics, University of Warwick, Coventry, UK, CV4 7AL, **Associate Professor**

Correspondence to:

Katharine Weetman
Warwick Medical School, University of Warwick, Coventry, UK, CV4 7AL
Katharine.Weetman@warwick.ac.uk

Word count (excluding abstract & tables): 4,641

Key words: Communication; discharge letter; hospital discharge; patient information; discharge summaries, copy letters, doctor patient communication.

Study area: General health/ communication

For peer review only - http://bmjopen.bmj.com/site/about/guidelines.xhtml

TITLE

The Discharge Communication Study: A Realist Evaluation of Discharge Communication Experiences of Patients, General Practitioners, and Hospital Practitioners, Alongside a Corresponding Discharge Letter Sample

ABSTRACT (300 words)

Objectives: To develop a programme theory for the intervention of patients receiving discharge letters.

Design: We used a realist evaluation approach and captured multiple perspectives of hospital discharge to refine our previously developed programme theory. General practitioner (GP), patient and hospital clinician views of a single discharge event in which they were all involved were collected using semi-structured interviews and surveys. These were then triangulated to match the corresponding discharge letter. Data were qualitatively synthesised and compared in meta-matrices before interrogation with realist logic of analysis to develop the programme theory that maps out how patients receiving discharge letters works in specific contexts.

Setting: 14 GP practices and four hospital trusts in West Midlands, UK.

Participants: 10 complete matched cases (GP, patient, and hospital practitioner), and a further 26 cases in which a letter was matched with two out of the three participants.

Results: We identified 7 context mechanism outcome configurations not found through literature searching. These related to the broad concepts of: patient preference for receiving letters, patient comprehension of letters, patient-directed letters, patient harm, and clinician views on patients receiving letters. "Patient choice" was important to the success (or not) of the intervention. Other important contexts for positive effects included: letters written in plain English, lay explanations for jargon, verbal information also provided, no new information in letter, and patient choice acknowledged. Three key findings were: patient understanding is perhaps greater than clinicians perceive, clinician attitudes are a barrier to patients receiving letters, and that, negative outcomes more commonly manifested when patients had not received letters, rather than when they had.

Conclusions: We suggest how patients receiving discharge letters could be improved to enhance patient outcomes. Our programme theory has potential for use in different healthcare contexts and as a framework for policy development relating to patient discharge.

ARTICLE SUMMARY

Strengths and limitations of this study

- First study to compare and contrast matched views of patients, general practitioners and hospital clinicians in relation to specific discharge letters.
- Realist theory facilitated understanding of not just whether patients should receive letters, but how this practice may "work" in different contexts and why.
- The qualitative methods enabled detailed gathering of the experiences, viewpoints, and attitudes of participants.
- The secondary analysis was limited by weaknesses in the primary dataset, including the sociodemographic diversity of the patients, range of conditions, and limited numbers of cases in which hospital clinician perspectives could be matched to those of GPs and patients.
- Evidence relating to children, mental health admissions, and those lacking capacity was not considered.

For peer review only - http://bmjopen.bmj.com/site/about/guidelines.xhtml

INTRODUCTION

Background

Effective communication during discharge care transitions is essential for patient safety and to reduce negative outcomes [1] such as hospital readmissions [2]. Despite this, studies [3-5] continue to reiterate that processes and content of discharge communication require improvement. Internationally, the practice of patients receiving letters varies but it is generally common for hospital doctors to write directly to general practitioners (GPs) or equivalent [6]. UK standards and policies [7-11] currently outline that patients should receive copies of letters between physicians as a "right" [11] and that this is "good practice" [7], unless there is risk of harm. Initiatives such as "please write to me" [8] by the *Academy of Medical Royal Colleges* have sought to increase practice of patients receiving letters and suggested modifications such as using plain English to increase patient comprehensibility. A recent (2020) review by Rayner *et al.* [6] highlighted the value of writing to patients in order to enhance collaborative working and positive outcomes. Despite this, research [12-14], both within the UK and internationally, continues to report that patients receive letters inconsistently, the effects of which are unclear [14, 15]. Reasons for this inconsistency are little understood but physician attitudes such as concerns about perceived harm may be acting as barrier to policy uptake which has implications for patient experience and safety [14]. It is important to understand the extent to which this occurs purposefully, and how this affects patient experience and outcomes.

Our previous realist review [14] found conflicts between clinician and patient perspectives in relation to patients receiving discharge letters (e.g. perceived rates of patient understanding). Hence, the current study was designed to shed light on reasons for conflicts through investigating experiences from multiple viewpoints within the same discharge events. The objectives were to undertake an investigation of how patients receiving discharge letters may be improved alongside best practice recommendations and to develop a programme theory for patients receiving letters. As outlined in the work of Pawson [16-19], a "programme theory" is useful as it goes beyond consideration of "does it work" and instead seeks to explain *how* an intervention may be theorised to "work" to include within what contexts, for whom, why and to what extent [16, 20]. The research questions were:

1. How do the experiences of patients, GPs, and hospital practitioners differ and align within the multi-perspective discharge communication cases?
2. How does patients receiving discharge letters work (or not) and what are the important contexts associated with the desired positive effects?

For peer review only - http://bmjopen.bmj.com/site/about/guidelines.xhtml

This is the final paper in a series forming the Discharge Communication Study [21]; the others are summarised in box 1. Results relating to the GPs and patients are published [22, 23].

Box 1 Summary of discharge communication studies and results

GP study [22]

Methods

- 53 GPs were recruited from 18 practices within the West Midlands (UK) through the local Clinical Research Network and Warwick Medical School links with practices.
- They were asked to purposively sample [24] 14-24 recent (<3 weeks) discharge letters in accordance with the inclusion and exclusion criteria (see table 1)
- Each GP completed a discharge letter selection template (see supplementary file 1) with their discharge letter grading (successful or unsuccessful) and their comments.
- A subgroup of 26 GPs took part in an audio recorded interview or focus group; these took place face to face at GP practices and over the telephone (see supplementary file 2 for interview guide).

Main findings

- Key components within discharge letters (e.g., GP actions) associated with successful gradings.
- The importance of clarity and comprehensibility.

Patient study [23]

Methods

- The patients associated with each of the letters sampled by GPs were invited to take part in a 1-1 semi-structured interview at their home or GP surgery (see supplementary file 3 for interview guide).
- No relationship was established with participants prior to the study.
- All interview/focus group data were audio recorded and transcribed by KW who also took notes. Transcripts were not shown to participants.

Main findings

- 50 patients to whom the sample letters related took part in interviews.
- They generally wanted to receive copies of their discharge communication letter.
- Patients also suggested how letter comprehensibility may be improved (e.g., no acronyms).

Hospital practitioner study

Methods

- The hospital practitioners who wrote the letters sampled by GPs were invited to take part in a survey.

Main findings

- 46 hospital practitioners completed surveys.
- There were differences between what clinicians felt should be done and what occurred in practice e.g., 26 (56.5%) felt patients should always receive letters and 17 (37.0%) did this in practice.
- Some hospital practitioners expressed reservations around patients receiving letters.
- Many were unaware of the Department of Health guidelines on copying letters to patients [7].

For peer review only - http://bmjopen.bmj.com/site/about/guidelines.xhtml

METHODS

Study design

This study was a secondary analysis of a subset of data from the Discharge Communication Study, an exploratory mixed methods study based in the West Midlands, United Kingdom (UK) [21]; box 1 gives a brief summary of papers linked to the Discharge Communication Study. The intervention under scrutiny 'patients receiving discharge letters' was defined by the team as 'the patient being given or sent any form of written (paper or digital) hospital discharge communication; this could be a direct copy, patient-directed letter, or a combination.' Broadly, the data comprised three elements: (1) GP sampling and rating of discharge letters ("successful" or "unsuccessful") and narrative interviews, (2) semi-structured interviews with patients to whom the letters related, (3) survey of hospital practitioners who wrote the sampled letters.

Settings

The setting for the study is outlined in the published study protocol [21]. It involved four hospital trusts and a diverse range of 18 GP practices in the West Midlands.

Recruitment and data collection

Recruitment and data collection took place, as detailed in previous publications [21-23, 25] between August 2017 and September 2018. In brief, GPs were asked to screen (see table 1 for screening criteria) and select a sample of recently received discharge letters according to what they considered to be "successful" or "unsuccessful" letter exemplars; for each letter, GPs were asked to complete the selection proforma (supplementary file 1) and rate the letters "successful" or "unsuccessful" [25]. There were no set criteria for letter ratings as the selection was based on each participating GP's interpretation of what makes a successful or unsuccessful discharge letter [25]. This purposive [24] letter sampling approach was intended to increase sample diversity and address the research questions within dichotomous contexts. All GPs involved in letter sampling were then invited to take part in a "narrative" [26] interview or focus group with KW (see supplementary file 2 for interview guide). All patients associated with the sampled discharge letters were sent an invitation pack by their GP practice; this invited them to take part in an audio recorded semi-structured interview with KW (see supplementary file 3 for interview guide). Finally, the hospital

For peer review only - http://bmjopen.bmj.com/site/about/guidelines.xhtml

professionals who wrote or signed the sampled discharge letters were sent an invitation pack by the research team; this invited them to take part in a survey on their evaluation of the discharge letter they wrote, their current practices, and their views about how discharge communication processes may be improved [25]. Packs were sent by post and email as well as being internally distributed by hospital sites.

For this study, we re-interpreted data collected across all of the other studies. This involved a secondary analysis of a subset of the data which was drawn from sampled discharge letters that could be "matched" to at least two other dataset perspectives. Study specific ID codes allocated to the letters allowed cross-matching with participants to build multiple viewpoint cases termed "quartets" (mapping together four elements if complete, or "trios" if only one perspective missing - see figure 1).

The target was to build 30 quartet cases through recruiting at least 30 GPs, patients and hospital practitioners (HPs) (target n=90). Trio and quartet participants were not separately recruited from other studies within the project; instead, cases were built through the participant recruitment and data collection across all studies for the discharge communication project (see figure 2). Once participant data across studies were matched into trio and quartet cases, findings and data were subjected to a secondary level data analysis using a realist approach described below. This allowed highlighting of data convergence and divergence as well as the emergence of new findings which only became apparent through juxtaposition.

Table 1 Discharge letter inclusion and exclusion criteria [21]

Inclusion criteria	• NHS adult (18+ years) patients recently discharged (≤3 weeks) from hospital following an episode of inpatient or outpatient care.<br>• Patient registered with the participating GP practice.<br>• Patient treated at and discharged from hospital trusts within Warwickshire, Coventry, Rugby, Herefordshire and Worcestershire.<br>• Cases where written discharge communication has been sent to the patient's GP.
Exclusion criteria	• Age <18 years.<br>• Patients who lack capacity to give informed consent to participate in the study (e.g., Alzheimer's, severe mental illness etc.) or are deemed by the GP to be unsuitable for participation (e.g., end of life).<br>• Patients discharged to providers or units other than their GP (e.g., discharge from hospital to a rehab unit).<br>• Discharge communication from mental health services.<br>• Communication about individuals who are considered unable to participate in an interview or focus group or survey conducted in English.<br>• Letter relates to patient who has expressed a general wish not to participate in research.

For peer review only - http://bmjopen.bmj.com/site/about/guidelines.xhtml

Analysis

The study was underpinned by a critical realist framework [27] and a generative view of causation, that is, not just whether an intervention works but in what contexts, how, for whom, and why [20]. A realist logic of analysis [16-18, 27] has the potential to account for complexity; discharge communication is complex in many ways such that the letter form (i.e., typed or handwritten) and format (i.e., narrative or templated) as well as the communicative abilities and attitudes of both writers and recipients may vary. This study took a pragmatic approach to realist evaluation [17, 28, 29] in order to apply realist logic to multiple perspective cases within single discharge events. The study drew on realist principles to generate a "programme theory" or theorised explanation of whether or not patients receiving letters "works" (or not) as well as outlining the important relating context [C], mechanism [M], and outcome [O] configurations (CMOCs). The programme theory from our previously conducted realist review [14] was used as the starting theory; this was further developed based on the primary data results and findings. Interrogation and synthesis of evidence for CMOCs used a realist analytic approach [18] to consider the same theory of whether or not "patients receiving letters" works in comparative settings [30]. Thus, analysis was grounded on the assumption that "outcomes" of the intervention may vary according to "context" [30]. All data were inspected for evidence of *"relevance"* [20, 30, 31] to the theory. Manual note-taking on data were then undertaken [14] and judgements were formed as to what any new CMOCs might plausibly be prior to integration into the programme theory.

Data relating to each group was initially analysed separately (see box 1). Findings across groups were then triangulated and a secondary analysis was undertaken using meta-matrices to compare and contrast data. Such triangulation has previously been used within healthcare research [32, 33], particularly in relation to healthcare consultations [34-36], to compare multiple perspectives. Multi-perspective case analysis involved re-review of the data for each case; findings from different participants within letter cases were re-read and juxtaposed to highlight agreements and disagreements. Narrative summaries for each case were then developed. Summaries were not intended to be comprehensive but select and include findings of relevance to the research questions. Analysis sought to reconcile previously identified literature disparities on this topic (see our realist review [14]) through highlighting source convergence and divergence in relation to "patients receiving letters".

For peer review only - http://bmjopen.bmj.com/site/about/guidelines.xhtml

Patient and public involvement

Around 30 patients were involved in the research design through identifying research priorities [37] by "ranking" potential research questions through completing surveys and taking part in discussions. Four persons with experience as carers from a pre-established panel also provided feedback on the readability and clarity of the patient information materials.

RESULTS

Recruitment

Figure 2 shows how data collection across all studies for the discharge communication project led to the formation of 26 trio cases (1 GP and HP, 3 patient and HP, 22 patient and GP) and 10 quartet cases (patient, GP, and HP). Table 2 summarises the data characteristics in terms of GP grading, patient gender and age, discharge episode type (inpatient, outpatient…), specialty, and hospital practitioner grade. The 10 quartet cases had an even divide of GP graded successful and unsuccessful letters. Four patients reported that they had previously received the discharge letter and six reported that they had not. Letters related to 6 specialties across four hospital trusts.

Table 2 trio and quartet characteristics

Characteristic	Trio cases (n=26)	Quartet cases (n=10)
GP grading	Successful: 18 (69.2%)<br>Unsuccessful: 8 (30.8%)	Successful: 5 (50.0%)<br>Unsuccessful: 5 (50.0%)
No. of GP practices and GPs	14 GP practices, 17 GPs	8 practices 9 GPs
Practice sizes	Small (<5,000 patients): 1 (7.1%)<br>Medium (5-10,000 patients): 8 (57.2%)<br>Large (10,000+ patients): 5 (35.7%)	Small (<5,000 patients): 0 (0.0%)<br>Medium (5-10,000 patients): 4 (50.0%)<br>Large (10,000+ patients): 4 (50.0%)
Patient age	Range: 27-87<br>Median: 67	Range: 59-77<br>Median: 71
Patient gender	Female: 14 (53.8%)<br>Male: 12 (46.2%)	Female: 3 (30.0%)<br>Male: 7 (70.0%)
Admission	Inpatient: 20 (76.9%)<br>Outpatient: 2 (7.7%)<br>Other*: 4 (15.4%)	Inpatient: 7 (70.0%)<br>Outpatient: 1 (10.0%)<br>Other*: 2 (20.0%)
Specialties	1. Urology: 2 (7.7%)<br>2. Respiratory: 1 (3.8%)<br>3. Accident & Emergency: 4 (15.5%)<br>4. General Surgery: 3 (11.5%)<br>5. Cardiology: 2 (7.7%)	1. Urology: 3 (30.0%)<br>2. Respiratory: 2 (20.0%)<br>3. Accident & Emergency: 1 (10.0%)<br>4. General Surgery: 2 (20.0%)<br>5. Cardiology: 1 (10.0%)<br>6. Trauma & Orthopaedics: 1 (10.0%)

For peer review only - http://bmjopen.bmj.com/site/about/guidelines.xhtml

	6. Trauma & Orthopaedics: 4 (15.5%)<br>7. Head and Neck: 1 (3.8%)<br>8. Endocrinology: 1 (3.8%)<br>9. Plastic Surgery: 1 (3.8%)<br>10. Neurosurgery: 1 (3.8%)<br>11. General Medicine: 4 (15.5%)<br>12. Internal Medicine: 1 (3.8%)<br>13. Renal Medicine: 1 (3.8%)	
Hospital grade of discharging physician	2 grade types:<br>Consultant: 20 (76.9%)<br>Core trainee or equivalent: 6 (23.1%)	4 grade types:<br>Consultant: 6 (60%)<br>Advanced clinical practitioner: 1 (10%)<br>Junior doctor: 2 (20%)<br>Senior house officer: 1 (10%)

**Other may include but not be limited to admission types such as accident and emergency visit, day case procedure, or speciality assessment unit visit.*

Context mechanism outcome configurations

Narrative summaries for our data are in supplementary file 4 (trios) and 5 (quartets). Following a realist approach, findings were interrogated for theories and CMOCs of "relevance" (20, 30, 31) to patients receiving discharge letters. The following section describes the identified CMOCs and concepts. Sub-heading themes which structured our realist review (14) were used and iteratively modified. The 48 CMOCs from the realist review were also systematically interrogated in light of the new evidence; 7 new CMOCs were added. The final table of 55 CMOCs is in supplementary file 6.

Patient preference/choice

Of the 36 cases, 26 patients had received the discharge letter and 10 had not. Patients frequently emphasised positive effects of receiving letters such as increased satisfaction and a sense of involvement (12, 38) [CMOC2]. Patients explained that receiving letters can increase their autonomy and so encourage them to take control and *"ownership"* of their health [CMOC5, CMOC14]. In cases where patients had not received letters (C-E, H-J), patients reported difficulty retaining information and feeling unclear about what happened, their condition and how to manage it. On the other hand, in cases where patients had received letters [context, C] (A, B, F, G), patients reported feeling informed and finding the letter useful as a reminder [mechanism, M] of what happened to increase recall (39, 40) [outcome, O] [CMOC15] and decrease the need to memorise information [CMOC50].

For peer review only - http://bmjopen.bmj.com/site/about/guidelines.xhtml

Past studies, across a range of settings, report that patient preference for receiving letters is high (79%-97%) [39-46]; this study supports this finding as patients generally indicated preference for discharge letter receipt. Despite this, both GPs and patients noted the inconsistent practice of patients receiving letters. A potential suggested solution was for letters to contain a template "tick box" [C] as to whether or not the patient has been given a letter copy so that it can be audited [O] and increase awareness of the practice [M] [CMOC49]. One new CMOC that emerged was that patients may use the letter [M] as a record [C] for providing evidence for administrative proceedings [O] (e.g., benefits) [CMOC51] or for care within unfamiliar settings (e.g., holidays). Broadly, impacts on patients' experiences were framed as more positive when patients had received discharge letters and more negative when they had not. Crucially, positive outcomes were typically only triggered within key contexts (e.g., letter factually accurate [CMOC15]). Our realist review found patients generally did not object to social habits being included in the letter as long as it had relevance [14]; our findings here caveated this notion in that this information should also be phrased with neutral non-judgemental language [C] to reduce likelihood of upset [M] which could diminish wellbeing [O] [CMOC53]. Crucially, patient preference was not 100% and it is important to consider those who may not wish to receive letters [CMOC40] through acknowledgments of *patient choice* [12, 41-43] [CMOC41]. Moreover, some patients may want to receive letters some of the time but not for every single care episode; patients identified this may apply in cases of repeat admissions for the same condition [C] where letters may be repetitive and not helpful [M] and so not requested [O] [CMOC52]. Systems of letter receipt must therefore account for individual case variation.

Patient comprehension

Findings supported previous evidence [41, 45, 47, 48], that patients may understand their letters [M] leading to improved patient knowledge and recall [O] as well as patients feeling empowered to take responsibility for their own health and so carrying out recommendations [CMOC12-15, CMOC54]. However, letters are not always stylistically tailored to patients' needs due to the presence of medical jargon and acronyms. Within some cases (e.g., case 6), the patient and GP agreed that the patient would have benefitted from use of lay terms in the letter to unravel the medical jargon. Case 5 highlighted that unexplained acronyms should be avoided for the sake of both patient and GP comprehensibility. There is a risk that patients receiving letters [C] may increase appointments or queries [O] as patients seek explanations of the letter contents [M] [49]. Nevertheless, in line with past work [46, 50], findings

For peer review only - http://bmjopen.bmj.com/site/about/guidelines.xhtml

were that this rarely occurs and indeed no study patients reported having made appointments for this purpose [CMOC7, CMOC11]. Furthermore, patients reported that the absence rather than receipt of the letter is what would prompt them to visit the GP [M] and thus increased patient information [C] may reduce rather than increase appointments [O] [CMOC11]. GPs suggested use of a "patient information" section on the letter [C] which provides a letter synopsis in the form of a lay summary to increase understanding [M] and improve patient knowledge and satisfaction [O] [CMOC54]. Patients and GPs agreed that letters should complement rather than substitute verbal information. This is seen in case 17 where the letter communicates a serious diagnosis to the patient and they report being given no other information from the hospital. Hence, letters should only be provided in the context of adequate patient counselling so that the letter is not communicating new information.

Personalised or patient-directed discharge letters

Personalised letters may increase resource use and workload (45, 48, 51) [CMOC25]. There were disagreements as to whether it would be more beneficial for patients to receive a separate personalised letter or the same letter as the GP; some clinicians felt personalised letters may improve patient comprehension (e.g., case 1) whereas patients generally preferred to receive the same copy as the GP for transparency and reassurance (e.g., case 3, 22, 23) [CMOC26]. Patients did suggest letter improvements in cases where the clinicians rated the letter successfully (cases B, I); patients felt letters should contain more information regarding how they can improve their condition and recommended patient actions.

Patient harm

Clinicians sometimes had concerns that patients receiving letters may cause harm such as patient anxiety or confusion. However, clinician concern was expressed in several cases where the patients emphasised the benefits of discharge letter receipt (cases B, C, E, G, H). Patients suggested that receiving letters [C] may reduce negative outcomes through reassuring them and reducing or settling anxiety [M] thereby supporting their wellbeing [O] [CMOC39] (case 8). Instances which subverted this trend primarily related to the letter quality (e.g., letter inaccuracies caused stress). One patient found that clear written information in bad news contexts [C] was particularly useful [M] as it allowed them to make

For peer review only - http://bmjopen.bmj.com/site/about/guidelines.xhtml

an informed end of life plan [O]. Suggestions to reduce risk of harm included ensuring the content is wholly factual and ensuring the patient consents to letter receipt [52] [CMOC41].

Clinician views

Supporting past literature, some clinicians were in favour [50, 53] [CMOC5, CMOC16] of the practice whilst others had reservations [12, 47] [CMOC6, CMOC35]. GPs appeared to be more in favour than hospital practitioners. Nonetheless, some GPs did express issues regarding the inherent need of letters to contain technical information which may not be patient comprehensible. Hospital practitioner concerns included: patient confusion and anxiety [13, 38, 44] [CMOC19], that the patient will not find the letter useful, that letters would need to be oversimplified [12, 54], and that receiving a letter may not be in the best interests of the patient (e.g. mental health cases). Clinician and GP perceived benefits [CMOC5] of patients receiving letters were: increased sense of patient inclusion, improved understanding or knowledge [51, 54], and increased transparency [47] [CMOC33]. Our realist review [14] suggested that patient understanding of their letters may be higher than clinicians perceive; this study further supports this notion. Comparably to previous literature, concern regarding "patient understanding" was common [12, 38, 47, 54] [CMOC6]. However, clinician and patient views were sometimes the antithesis of one another; there were cases where the clinician had concerns [C] regarding patient comprehensibility [M] in cases where the patient reported to have found the letter useful [O][CMOC55] (see cases A-C, E, G-H, J). Patients demonstrated resourcefulness through expounding that unknown terms can be looked up on the internet (case 19) as well as discretion [C] through appreciating that understanding the contents and implications [O] may not necessarily involve comprehending every word [M].

Programme theory

Our findings were used to refine the programme theory, using our realist review [14] as the starting point; changes made to the theory are highlighted in bold (see figure 3). All matched cases were re-read, annotated and interrogated for evidence. Relevant evidence [30, 31] was inspected and concepts drawn on to form the resultant programme theory in figure 3 which shows two main channels: patient copies of letters and patient personalised letters. Contexts for when patients receive letters still contained five key contexts for when this

For peer review only - http://bmjopen.bmj.com/site/about/guidelines.xhtml

intervention does work but context details were modified. Previously, the theory had four key contexts for when the intervention is theorised not to work; these were updated to include the new context of judgemental language in relation to social behaviour [CMOC53]. Outcomes of patients receiving separate personalised letters were modified; new negative outcomes were overly "basic" content and perceived potential secrecy between clinicians if they are sending and receiving separate letters. "Patient choice" was still a key influencer for likelihood of beneficial outcomes, and contextual influences such as resource provision and directives [CMOC49] were determiners of patients being given a choice of letter receipt [CMOC52].

DISCUSSION

Summary of findings

We undertook a realist evaluation [19, 28, 55, 56] to explore patient, GP and hospital clinician experiences of written discharge communications and hence test and refine the programme theory from our previous realist review [14]. The modified programme theory (figure 3) maps out how patients receiving discharge letters works in specific contexts leading to different positive and negative outcomes. Positive outcomes and positive pathway components are indicated in figure 3 via green coloured text boxes whereas negative outcomes and negative pathway components are indicated in red. Any neutral components or those which can be either positive or negative (e.g., attitudes of clinicians) are in black. Analysis of the multi-perspective discharge events led to the emergence of findings not found in our previous review. Several changes to the initial theory were made to include 10 CMOC modifications and the addition of 7 new CMOCs not found through previous literature searching. No CMOCs were removed. Key contexts for positive outcomes included: letters written in plain English, lay explanations for jargon, written and verbal information provided, no new information in letter, and patient given choice of letter receipt.

While benefits [42, 57] and drawbacks [54, 58] of patients receiving discharge letters have been previously suggested, our study adds an understanding of *how* patients receiving letters *works* through outlining the important contexts and associated mechanisms that explain outcome patterns [59, 60]. In addition, the multi-perspective analysis provided possible explanations for previously reported discrepancies identified through our realist review [14]. One example of a discrepancy was that past work highlighted conspicuously inconsistent rates of patient understanding [12, 41, 47, 48, 61, 62]. Data from this study revealed that even in cases where clinicians expressed concerns, patients generally reported to have understood

For peer review only - http://bmjopen.bmj.com/site/about/guidelines.xhtml

the letter and found it useful. Furthermore, patients often preferred receiving the same letter as the GP rather than a separate letter. Another disparity was in relation to “negative outcomes”. A common clinician concern within the study and past literature (13, 38, 44) was that patients receiving letters may cause anxiety and harm. However, literature also reported that patients may find letters useful (12, 45, 48). Our method highlighted that in several cases where clinicians had concerns, patients who received letters tended to emphasise the positive effects (e.g., increased knowledge). Indeed, patients stressed negative outcomes in contexts where they *had not* rather than *had* received letters. Some patients reported that receiving the letter alleviated anxiety thereby supporting their wellbeing through informing them of their admission, and any next steps, as well as providing reassurance that their GP was updated.

Strengths and weaknesses of the study

We followed RAMESES standards for realist evaluation (29, 63) and completed the COREQ checklist by Tong et al. (64). To the best of our knowledge, this is the first study to triangulate matched perspectives of patients and clinicians in relation to specific discharge letters. This allowed reconciliation of disparities in the literature and so enabled refinement of the programme theory. Grounding the research in realist theory strengthened the applicability of findings as it facilitated an understanding of not just whether patients should receive letters, but how this practice may “work” as well as in what contexts and why (16, 17).

As with other realist evaluations (65), the results and findings are intended to have wide applicability to other settings, in this case, settings where adults may receive hospital discharge letters. However, it is important to note the contexts and those groups who were excluded or were under-represented in this study. The exclusion criteria restricted the programme theory such that evidence relating to children, solely to mental health, and those lacking capacity to consent was not considered. Moreover, participation bias may have resulted in the views of ethnic minorities and other marginalised groups being under-represented. The main weakness of the study was the small sample sizes in terms of numbers of patients, sociodemographic diversity of the patients, and range of conditions; for many of the discharge letters it was not possible to form a complete quartet. The study fell short of the target of building 30 quartets; the primary reason for this was under-recruitment of hospital practitioners. The low response rate of hospital practitioners was likely impacted by their lack of available time, our survey recruitment strategy, hospital rotations, and the time lapse between the practitioner writing the letter and receiving the survey invitation. The programme theory would have benefitted from being informed by a larger and more diverse sample of primary evidence. The matched cases relate to a specific geographic area and

For peer review only - http://bmjopen.bmj.com/site/about/guidelines.xhtml

hence will not have reflected the full range of hospital discharge communication practices that are present nationally. Analysis cannot be considered to be wholly objective due to the influence of researcher identity [66]. Therefore, “reflexivity” was practised throughout the research to reduce but not eradicate bias [66, 67]. Reflexivity was practised through keeping a research diary and regular research team discussion and reflection. Data analysis was also limited by the available evidence which was thin in relation to: dictating letters, the cost of patients receiving letters, doctor-patient relationships, and reasons for variation of practice. Further research is needed to explore these areas as well as the relevance of the programme theory to excluded and under-represented groups, such as those without capacity and children.

Meaning of the study: implications for clinicians and policy makers

The programme theory generated by this study draws on our previous review and primary data, and hence reflects evidence from 16 countries and over 16,000 participants. As such, the theory has both national and international relevance and is likely to be applicable to different healthcare settings. It generally supports policies [7-9, 11] that patients should be offered copies of letters between physicians. Although sending patients’ letters, to include discharge letters, has been recommended practice for almost 20 years [7], uptake remains inconsistent [12-14]. Although national guidelines exist [7-10, 68, 69], each hospital may have its own discharge policy; this means that patients may have different discharge experiences and receive different discharge communications depending on the hospital, discharging physician, and reason for admission, as exemplified in this study. This needs to be addressed with more standardised practices which account for individual preferences and are grounded by *patient choice* with the exception of where there is a risk of “harm”, as defined in guideline documents [7]. Patients have a right to receive their letters [11] and should not be denied the opportunity to receive letters based on the perception that their understanding may be low. Although patients may have limited health literacy, they demonstrated resourcefulness and resilience for accessing letter content by looking up unknown terms on the internet and also appreciated that understanding the important features and main directives of a letter does not necessarily involve comprehending every word. Thus, patient understanding is perhaps greater than perceived and the presence of clinical terminology alone is not reason enough to exclude patients from communications. Overall, our study found that negative outcomes more commonly manifested when patients had not received letters, rather than when they had. This included contexts where the clinicians had concern about patient understanding and yet the patient reported to have

For peer review only - http://bmjopen.bmj.com/site/about/guidelines.xhtml

found the letter of value. It may be inferred that within certain contexts, clinician concerns about patients receiving letters are perhaps unfounded. Thus, clinician attitudes and risk averse behaviour may be acting as a barrier to uptake of this practice.

This research has provided a modified programme theory which demonstrates how policy makers and clinicians may effectively involve patients in their care through provision of written communications. Our theory outlines how both positive and negative outcomes may be produced through this intervention and highlights the importance of contextual considerations [(56)]. As outlined in previous realist evaluations [(60)], an advantage of this approach is the relevance of the resultant theory to policy makers as it informs how policy may be adapted to particular purposes and the specific contexts needed to achieve the desired outcomes. An example is the importance of the contextual factor “patient choice of letter receipt” to producing positive outcomes; this is of relevance to policy makers as it explains how best practice of patients receiving letters may be adapted to “work” and how research may be implemented into practice and policy. Nonetheless, future work should endeavour to test and refine the programme theory through interrogation of new evidence and measurement of primary and secondary outcomes. This will support the development of interventions that lead to more effective communication between hospital and primary care health professionals, and hence positive patient outcomes.

CONCLUSION

Sharing information and effective discharge communication with patients should be a priority to improve patient experience and the safety of patient care. This study has yielded insights into ways in which practices of patients receiving discharge letters could be improved to enhance patient experience and outcomes. Key findings were: clinicians may underestimate patients’ capacity to comprehend discharge letters, patient choice is important for positive outcomes, absence rather than presence of information may be more associated with negative outcomes, and clinician attitudes may be acting as a barrier to patients receiving letters. Our programme theory draws on previous research and experiences of clinicians and patients. The theory has potential for use in different healthcare contexts and as a framework for policy development on patient discharge.

Acknowledgements: The authors would like to express thanks to the GPs, patients, and hospital practitioners who informed the study design as well as those who took part in the study. We would like to thank the West Midlands Clinical Research Network for their support with research recruitment. We would also like to thank the Institute of Advanced Study at the

For peer review only - http://bmjopen.bmj.com/site/about/guidelines.xhtml

University of Warwick for their support during the write-up of this paper. Finally, we would like to thank Hugh Rayner for providing feedback on an earlier version of the manuscript.

Author contributions: KW, ES, SS and JD substantially contributed to the design and conception of the work and collaborated with KW on data interpretation. KW drafted the manuscript which was then critically revised by JD, ES and SS. All authors approved the final manuscript for publication and agree to be accountable for all aspects of the work.

Funding: This work was supported by the Economic and Social Research Council [grant number ES/J500203/1] and Clinical Commissioning Groups of Coventry & Rugby and South Warwickshire. Funding for the open access charges for publication of this manuscript was provided by the UKRI fund. The funders had no involvement in analysis or preparation of the manuscript.

Competing interests: None declared.

Patient and public involvement: The public and other stakeholder groups were involved in the design and selection of research questions for this research.

Patient consent for publication: Not required.

Ethics approval: Ethics approval was granted by the UK Health Research Authority in July 2017 (Integrated Research Application System ID: 219871, Research Ethics Committee reference: 17/WM/0170, sponsor: University of Warwick).

Data sharing statement: The narrative summaries for data are provided as supplementary files. The full raw datasets for the study are not publicly available due to the sensitive and identifiable nature of the data. Despite names and other identifiers being removed, the in-depth nature of case information may mean that participants could be identified.

References

1.Groene R.O., Orrego C., Sunol R., *et al.* "It's like two worlds apart": an analysis of vulnerable patient handover practices at discharge from hospital. *BMJ Qual Saf*. 2012;21 Suppl 1:i67-75. Available from: https://qualitysafety.bmj.com/content/21/Suppl_1/i67.long [Accessed: 10/06/20].

2.Lorenzati B., Quaranta C., Perotto M., *et al.* Discharge communication is an important underestimated problem in emergency department. *Internal & Emergency Medicine*. 2016;11(1):157-8. Available from: https://dx.doi.org/10.1007/s11739-015-1351-0 [Accessed: 09/07/2020].

3.Rapport F., Hibbert P., Baysari M., *et al.* What do patients really want? An in-depth examination of patient experience in four Australian hospitals. *BMC Health Serv Res*. 2019;19(1):38.[Accessed: 17/07/2020].

4.Flink M., Bergenbrant Glas S., Airosa F., *et al.* Patient-centered handovers between hospital and primary health care: an assessment of medical records. *Int J Med Inform*. 2015;84(5):355-62.[Accessed: 10/06/20].

5.Beaton A., O'Leary K., Thorburn J., *et al.* Improving patient experience and outcomes following serious injury. *N Z Med J*. 2019;132(1494):15-25.[Accessed: 10/06/20].

6.Rayner H., Hickey, M., Logan, I., Mathers, N., Rees, P., Shah, R. Writing outpatient letters to patients. *BMJ*. 2020;368:m24. Available from: https://www.bmj.com/content/bmj/368/bmj.m24.full.pdf [Accessed: 10/07/2020].

7.*Department of Health*. Copying letters to patients: good practice guidelines. 2003. Available from

For peer review only - http://bmjopen.bmj.com/site/about/guidelines.xhtml

https://webarchive.nationalarchives.gov.uk/20120504030618/http://www.dh.gov.uk/prod_consum_dh/groups/dh_digitalassets/@dh/@en/documents/digitalasset/dh_4086054.pdf
8.The Academy of Medical Royal Colleges. Please, write to me: Writing outpatient clinic letters to patients. 2018. Available from https://www.aomrc.org.uk/reports-guidance/please-write-to-me-writing-outpatient-clinic-letters-to-patients-guidance/
9.National Institute for Health and Care Excellence (NICE). Patient experience in adult NHS services: improving the experience of care for people using adult NHS services. *Clinical guideline [CG138]*. 2012 https://www.nice.org.uk/guidance/cg138. Available from: https://www.nice.org.uk/guidance/cg138 [Accessed: 25/02/2019].
10.Professional Record Standards Body. Implementation guidance report eDischarge standard. Better records for better care2019. Available from https://theprsb.org/standards/healthandcarerecords/
11.*Department of Health*. The NHS Plan: A Plan for Investment a Plan for Reform. London: HMSO; 2000. Available from https://webarchive.nationalarchives.gov.uk/20121102184216/http://www.dh.gov.uk/en/Publicationsandstatistics/Publications/PublicationsPolicyAndGuidance/DH_4002960
12.Baxter S., Farrell K., Brown C., *et al.* Where have all the copy letters gone? A review of current practice in professional-patient correspondence. *Patient Educ Couns*. 2008;71(2):259-64. Available from: https://dx.doi.org/10.1016/j.pec.2007.12.002 [Accessed: 10/06/20].
13.Boaden R., Harris C. Copying letters to patients—will it happen? *Fam Prac*. 2005;22:141–3. Available from: https://academic.oup.com/fampra/article/22/2/141/522310 [Accessed: 09/07/2020].
14.Weetman K., Wong G., Scott E., *et al.* Improving best practice for patients receiving hospital discharge letters: a realist review. *BMJ Open*. 2019;9(6):e027588. Available from: https://bmjopen.bmj.com/content/bmjopen/9/6/e027588.full.pdf [Accessed: 10/06/20].
15.Harris E., Rob P., Underwood J., *et al.* Should patients still be copied into their letters? A rapid review. *Patient Educ Couns*. 2018;101(12):2065-82. Available from: https://www.sciencedirect.com/science/article/abs/pii/S0738399118303136?via%3Dihub [Accessed: 09/07/2020].
16.Pawson R. Middle range theory and program theory evaluation: From provenance to practice. In: Vaessen J., Leeuw, F.L., editor. *Mind the gap Perspectives on policy evaluation and the social sciences*. New Brunswick, NJ and London: Transaction Publishers; 2010. p. 171-203.
17.Pawson R. *The science of evaluation: a realist manifesto*. London: Sage; 2013.
18.Pawson R. *Evidence-based policy: a realist perspective*. London, UK: Sage; 2006.
19.Pawson R., & Tilley, N. *An introduction to scientific realist evaluation.* 1997 2020/09/02. In: Evaluation for the 21st Century: A Handbook [Internet]. Thousand Oaks, California: SAGE Publications, Inc.; [405-18]. https://methods.sagepub.com/book/evaluation-for-the-21st-century.
20.Pawson R., Greenhalgh T., Harvey G., *et al.* Realist review--a new method of systematic review designed for complex policy interventions. *J Health Serv Res Policy*. 2005;10 Suppl 1:21-34. Available from: https://journals.sagepub.com/doi/abs/10.1258/1355819054308530 [Accessed: 09/07/2020].
21.Weetman K., Dale J., Scott E., *et al.* The Discharge Communication Study: research protocol for a mixed methods study to investigate and triangulate discharge communication experiences of patients, GPs, and hospital professionals, alongside a corresponding discharge letter sample. *BMC Health Services Research*. 2019;19(1):825. Available from: https://doi.org/10.1186/s12913-019-4612-1 [Accessed: 10/06/20].

For peer review only - http://bmjopen.bmj.com/site/about/guidelines.xhtml

22.Weetman K., Dale J., Spencer R., *et al.* GP perspectives on hospital discharge letters: an interview and focus group study. *BJGP Open*. 2020 https://bjgpopen.org/content/4/2/bjgpopen20X101031. Available from: https://bjgpopen.org/content/4/2/bjgpopen20X101031 [Accessed: 10/06/20].
23.Weetman K., Dale J., Scott E., *et al.* Adult patient perspectives on receiving hospital discharge letters: a corpus analysis of patient interviews. *BMC Health Services Research*. 2020;20(1):537. Available from: https://doi.org/10.1186/s12913-020-05250-1 [Accessed: 24/6/20].
24.Teddlie C., Yu F. Mixed methods sampling: A typology with examples. *Journal of mixed methods research*. 2007;1(1):77-100. Available from: https://journals.sagepub.com/doi/10.1177/1558689806292430 [Accessed: 09/07/2020].
25.Weetman K., Spencer R., Dale J., *et al.* What makes a "successful" or "unsuccessful" discharge letter? Hospital clinician and General Practitioner assessments of the quality of discharge letters. *BMC Health Services Research*. 2021;21(1):349. Available from: https://doi.org/10.1186/s12913-021-06345-z [Accessed: 18/05/21].
26.Stuckey H.L. Three types of interviews: Qualitative research methods in social health. *J Diabetes Res & Clin Prac*. 2013;1(2):56.
27.Pawson R., Tilley N. *Realistic Evaluation. Evaluation*. London: Sage; 1999.
28.Jagosh J., Bush P.L., Salsberg J., *et al.* A realist evaluation of community-based participatory research: partnership synergy, trust building and related ripple effects. *BMC Public Health*. 2015;15:725. Available from: https://pubmed.ncbi.nlm.nih.gov/26223523/ [Accessed: 21/7/2020].
29.Wong G., Westhorp G., Greenhalgh J., *et al.* Quality and reporting standards, resources, training materials and information for realist evaluation: the RAMESES II project. *NIHR Journals*. 2017;Health Services and Delivery Research(5). Available from: https://pubmed.ncbi.nlm.nih.gov/29072890/ [Accessed: 21/7/2020].
30.Pawson R., Greenhalgh, T., Harvey, G. & Walshe, K. . Realist synthesis: an introduction.' *ESRC Research Methods Programme*. 2004 Available at: https://goo.gl/1Rz2Ry. Available from: Available at: https://goo.gl/1Rz2Ry [Accessed: 04/01/17].
31.Pawson R. Digging for nuggets: how 'bad' research can yield 'good' evidence. *International Journal of Social Research Methodology*. 2006;9(2):127-42. Available from: https://www.tandfonline.com/doi/abs/10.1080/13645570600595314 [Accessed: 09/07/2020].
32.Farmer T., Robinson, K.. Elliott, S. J., Eyles, J. Developing and implementing a triangulation protocol for qualitative health research. *Qual Health Res*. 2006;16(3):377-94. Available from: https://journals.sagepub.com/doi/abs/10.1177/1049732305285708 [Accessed: 09/07/2020].
33.Begley C.M. Using triangulation in nursing research. *Journal of Advanced Nursing*. 1996;24(1):122-8. Available from: https://onlinelibrary.wiley.com/doi/abs/10.1046/j.1365-2648.1996.15217.x [Accessed: 09/07/2020].
34.Mendick N., Young, B., Holcombe, C., Salmon, P. The ethics of responsibility and ownership in decision-making about treatment for breast cancer: triangulation of consultation with patient and surgeon perspectives. *Soc Sci Med*. 2010;70(12):1904-11. Available from: https://www.sciencedirect.com/science/article/abs/pii/S027795361000225X?via%3Dihub [Accessed: 09/07/2020].
35.Salmon P., Mendick, N., Young, B. Integrative qualitative communication analysis of consultation and patient and practitioner perspectives: towards a theory of authentic caring in clinical relationships. *Patient Educ Couns*. 2011;82(3):448-54. Available from: https://www.sciencedirect.com/science/article/abs/pii/S0738399110006257?via%3Dihub [Accessed: 09/07/2020].

For peer review only - http://bmjopen.bmj.com/site/about/guidelines.xhtml

36.Durif-Bruckert C., Roux, P., Morelle, M., Mignotte, H., Faure, C., Moumjid-Ferdjaoui, N. Shared decision-making in medical encounters regarding breast cancer treatment: the contribution of methodological triangulation. *Eur J Cancer Care (Engl)*. 2015;24(4):461-72. Available from: https://onlinelibrary.wiley.com/doi/abs/10.1111/ecc.12214 [Accessed: 09/07/2020].
37.Cowan K. O., S. James Lind Alliance Guidebook. Southampton: James Lind Alliance; 2013.
38.Tomkins C.S., Braid J.J., Williams H.C. Do dermatology outpatients value a copy of the letter sent to their general practitioner? In what way and at what cost? *Clin Exp Dermatol*. 2004;29(1):81-6. Available from: https://onlinelibrary.wiley.com/doi/abs/10.1111/j.1365-2230.2004.01437.x?sid=nlm%3Apubmed [Accessed: 10/06/20].
39.Antoniou A., Saunders M., Bourner R., *et al.* would you like to see yours? *Bull R Coll Surg Engl*. 2007;89(2):62-4. Available from: https://publishing.rcseng.ac.uk/doi/10.1308/147363507X169936 [Accessed: 09/07/2020].
40.Krishna Y., Damato B.E. Patient attitudes to receiving copies of outpatient clinic letters from the ocular oncologist to the referring ophthalmologist and GP. *Eye (Lond)*. 2005;19(11):1200-4. Available from: https://www.nature.com/articles/6701740 [Accessed: 09/07/2020].
41.Fenton C., Al-Ani A., Trinh A., *et al.* Impact of providing patients with copies of their medical correspondence: a randomised controlled study. *Intern Med J*. 2017;47(1):68-75. Available from: https://onlinelibrary.wiley.com/doi/abs/10.1111/imj.13252 [Accessed: 10/06/20].
42.O'Driscoll B.R., Koch J., Paschalides C. Copying letters to patients: Most patients want copies of letters from outpatient clinics and find them useful. *BMJ*. 2003;327(7412). Available from: http://www.ncbi.nlm.nih.gov/pmc/articles/PMC188516/ [Accessed: 10/07/2020].
43.Rao M., Fogarty P. What did the doctor say? *J Obstet Gynecol*. 2007;27(5):479-80. Available from: https://dx.doi.org/10.1080/01443610701405853 [Accessed: 09/07/2020].
44.Treacy K., Elborn J.S., Rendall J., *et al.* Copying letters to patients with cystic fibrosis (CF): letter content and patient perceptions of benefit. *J Cyst Fibros*. 2008;7(6):511-4. Available from: https://www.cysticfibrosisjournal.com/article/S1569-1993(08)00061-1/fulltext [Accessed: 10/06/20].
45.Brodie T., Lewis D. A survey of patient views on receiving vascular outpatient letters. *Eur J Vasc Endovasc Surg*. 2010;39(1):5-10. Available from: https://www.ejves.com/article/S1078-5884(09)00500-0/fulltext [Accessed: 09/07/2020].
46.Sharma D., O'Brien S., Hardy K. Copying letters to patients: What patients think - A questionnaire survey. *Clinician in Manage*. 2007;15(2):75-8. Available from: https://www.ncbi.nlm.nih.gov/pmc/articles/PMC2121286/ [Accessed: 09/07/2020].
47.Baumann W., Schussler, L., Bertram, M., Benser, J., Kumpers, S., Hermes-Moll, K. Oncologists' letters for breast cancer patients. *Oncol Res Treat*. 2016;39:184-5. Available from: http://dx.doi.org/10.1159/000449050 [Accessed: 10/06/20].
48.Pinder E., Jefferys S., Loeffler M. Patient Satisfaction: Receiving a copy of the GP letter following fracture or elective orthopaedic clinic. *BMJ Qual Improv Rep*. 2013;2(2). Available from: http://bmjopenquality.bmj.com/content/bmjqir/2/2/u202144.w1085.full.pdf [Accessed: 09/07/2020].
49.Liapi A., Robb P.J., Akthar A. Copying clinic letters to patients: a survey of patient attitudes. *J Laryngol Otol*. 2006;121(6):588-91. Available from: https://www.cambridge.org/core/article/copying-clinic-letters-to-patients-a-survey-of-patient-attitudes/9683993BFBE9720C5C9C13741F285713 [Accessed: 17/07/2020].

For peer review only - http://bmjopen.bmj.com/site/about/guidelines.xhtml

50.Brockbank K. Copying patient letters - Making it work. *Clin Gov*. 2005;10(3):231-40. Available from: http://dx.doi.org/10.1108/14777270510627590 [Accessed: 10/06/20].
51.McConnell D., Butow P., Tattersall M. Audiotapes and letters to patients: the practice and views of oncologists, surgeons and general practitioners. *Br J Cancer*. 1999;79:1782-8.[Accessed: 10/06/20].
52.The Newcastle upon Tyne Hospitals NHS Foundation Trust. The Newcastle upon Tyne Hospitals NHS Foundation Trust: Sharing Letters with Patients Policy. 2019. Available from http://www.newcastle-hospitals.org.uk/downloads/policies/Operational/SharingLetterswithPatients201901.pdf
53.Bench S.D., Heelas K., White C., *et al.* Providing critical care patients with a personalised discharge summary: a questionnaire survey and retrospective analysis exploring feasibility and effectiveness. *Intensive & Critical Care Nursing*. 2014;30(2):69-76. Available from: http://www.intensivecriticalcarenursing.com/article/S0964-3397(13)00090-6/fulltext [Accessed: 17/07/2020].
54.O'Reilly M., Cahill M., Perry I.J. Writing to patients: 'putting the patient in the picture'. *Ir Med J*. 2005;98(2):58-60. Available from: https://pubmed.ncbi.nlm.nih.gov/15835515/ [Accessed: 09/07/2020].
55.Martin P., Tannenbaum, C. A realist evaluation of patients' decisions to deprescribe in the EMPOWER trial. *BMJ Open*. 2017;7(4):e015959. Available from: https://bmjopen.bmj.com/content/bmjopen/7/4/e015959.full.pdf [Accessed: 09/09/2020].
56.Crampton P., Mehdizadeh L., Page M., *et al.* Realist evaluation of UK medical education quality assurance. *BMJ Open*. 2019;9(12):e033614. Available from: https://bmjopen.bmj.com/content/bmjopen/9/12/e033614.full.pdf [Accessed: 09/09/2020].
57.Saunders N.C., Georgalas C., Blaney S.P., *et al.* Does receiving a copy of correspondence improve patients' satisfaction with their out-patient consultation? *J Laryngol Otol*. 2003;117(2):126-9. Available from: https://dx.doi.org/10.1258/002221503762624576 [Accessed: 09/07/2020].
58.Hallowell N. Providing letters to patients. Patients find summary letters useful. *BMJ*. 1998;316(7147):1830. Available from: https://www.bmj.com/content/316/7147/1830.3 [Accessed: 09/07/2020].
59.Kerr H., Price, J., Nicholl, H., O'Halloran, P. Facilitating transition from children's to adult services for young adults with life-limiting conditions (TASYL): Programme theory developed from a mixed methods realist evaluation. *International Journal of Nursing Studies*. 2018;86:125-38. Available from: http://www.sciencedirect.com/science/article/pii/S0020748918301536 [Accessed: 09/09/2020].
60.Willis C.E., Reid S., Elliott C., *et al.* A realist evaluation of a physical activity participation intervention for children and youth with disabilities: what works, for whom, in what circumstances, and how? *BMC Pediatrics*. 2018;18(1):113. Available from: https://doi.org/10.1186/s12887-018-1089-8 [Accessed: 09/09/2020].
61.Lin M.J., Tirosh A.G., Landry A. Examining patient comprehension of emergency department discharge instructions: Who says they understand when they do not? *Internal & Emergency Medicine*. 2015;10(8):993-1002. Available from: https://dx.doi.org/10.1007/s11739-015-1311-8 [Accessed: 09/07/2020].
62.Choudhry A.J., Baghdadi Y.M., Wagie A.E., *et al.* Readability of discharge summaries: with what level of information are we dismissing our patients? *American Journal of Surgery*. 2016;211(3):631-6. Available from: https://dx.doi.org/10.1016/j.amjsurg.2015.12.005 [Accessed: 09/07/2020].
63.Wong G., Westhorp G., Manzano A., *et al.* RAMESES II reporting standards for realist evaluations. *BMC medicine*. 2016;14(1):96-. Available from: https://pubmed.ncbi.nlm.nih.gov/27342217 [Accessed: 21/7/2020].

For peer review only - http://bmjopen.bmj.com/site/about/guidelines.xhtml

64.Tong A., Sainsbury P., Craig J. Consolidated criteria for reporting qualitative research (COREQ): a 32-item checklist for interviews and focus groups. *Int J Qual Health Care*. 2007;19(6):349-57. Available from: https://academic.oup.com/intqhc/article/19/6/349/1791966 [Accessed: 09/07/2020].
65.De Sutter M., De Sutter, A., Sundahl, N., Declercq, T., Decat, P. Inter-professional collaboration reduces the burden of caring for patients with mental illnesses in primary healthcare. A realist evaluation study. *European Journal of General Practice*. 2019;25(4):236-42. Available from: https://doi.org/10.1080/13814788.2019.1640209 [Accessed: 09/09/2020].
66.Malterud K. Qualitative research: standards, challenges, and guidelines. *The lancet*. 2001;358(9280):483-8. Available from: https://www.thelancet.com/journals/lancet/article/PIIS0140-6736(01)05627-6/fulltext [Accessed: 09/07/2020].
67.Mays N., Pope C. Qualitative research in health care: Assessing quality in qualitative research. *BMJ*. 2000;320(7226):50. Available from: https://www.ncbi.nlm.nih.gov/pmc/articles/PMC1117321/ [Accessed: 09/07/2020].
68.Royal College of Physicians. Standards for the clinical structure and content of patient records. 2013. Available from https://www.rcplondon.ac.uk/projects/outputs/standards-clinical-structure-and-content-patient-records
69.NHS Digital. The PRSB Standards for the Structure and Content of Health and Care Records. Professional Record Standards Body (PRSB); 2018. Available from https://theprsb.org/standards/healthandcarerecords/

List of figure headings

Figure 1 Multiple-perspective "quartet" case wherein comparisons occur between experiences associated with the same discharge letter

Figure 2 Recruitment uptake across studies for the project to show how trio and quartet cases were formed

Figure 3 Resultant programme theory that maps out how patients receiving discharge letters works (or not)

For peer review only

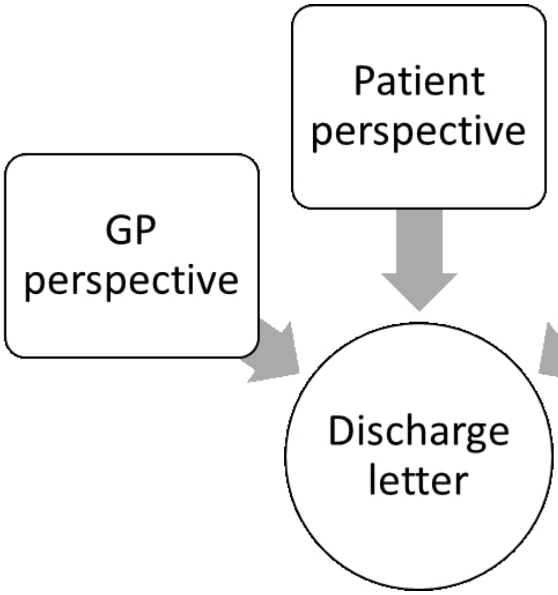

Figure 1 Multiple-perspective “quartet” case wherein comparisons occur between experiences associated with the same discharge letter

73x43mm (300 x 300 DPI)

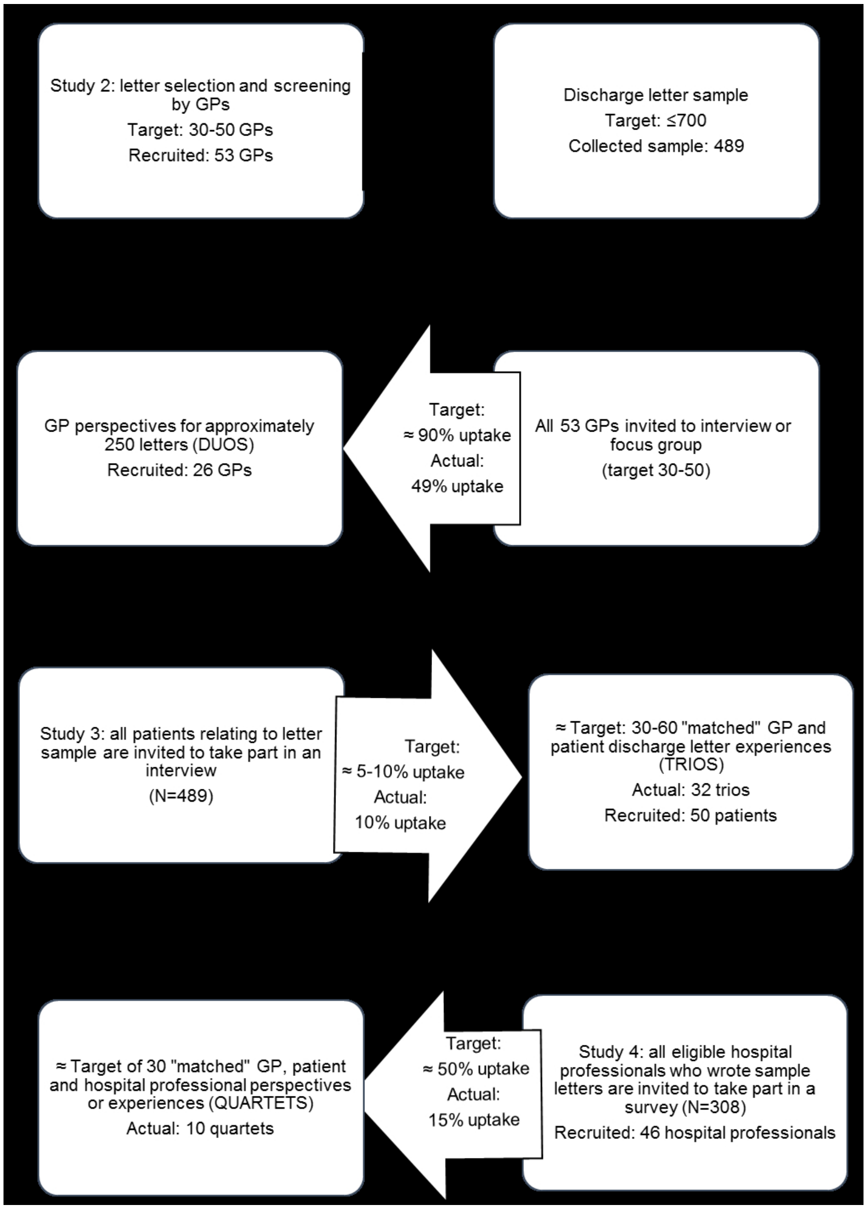

Figure 2 Recruitment uptake across studies for the project to show how trio and quartet cases were formed

85x119mm (300 x 300 DPI)

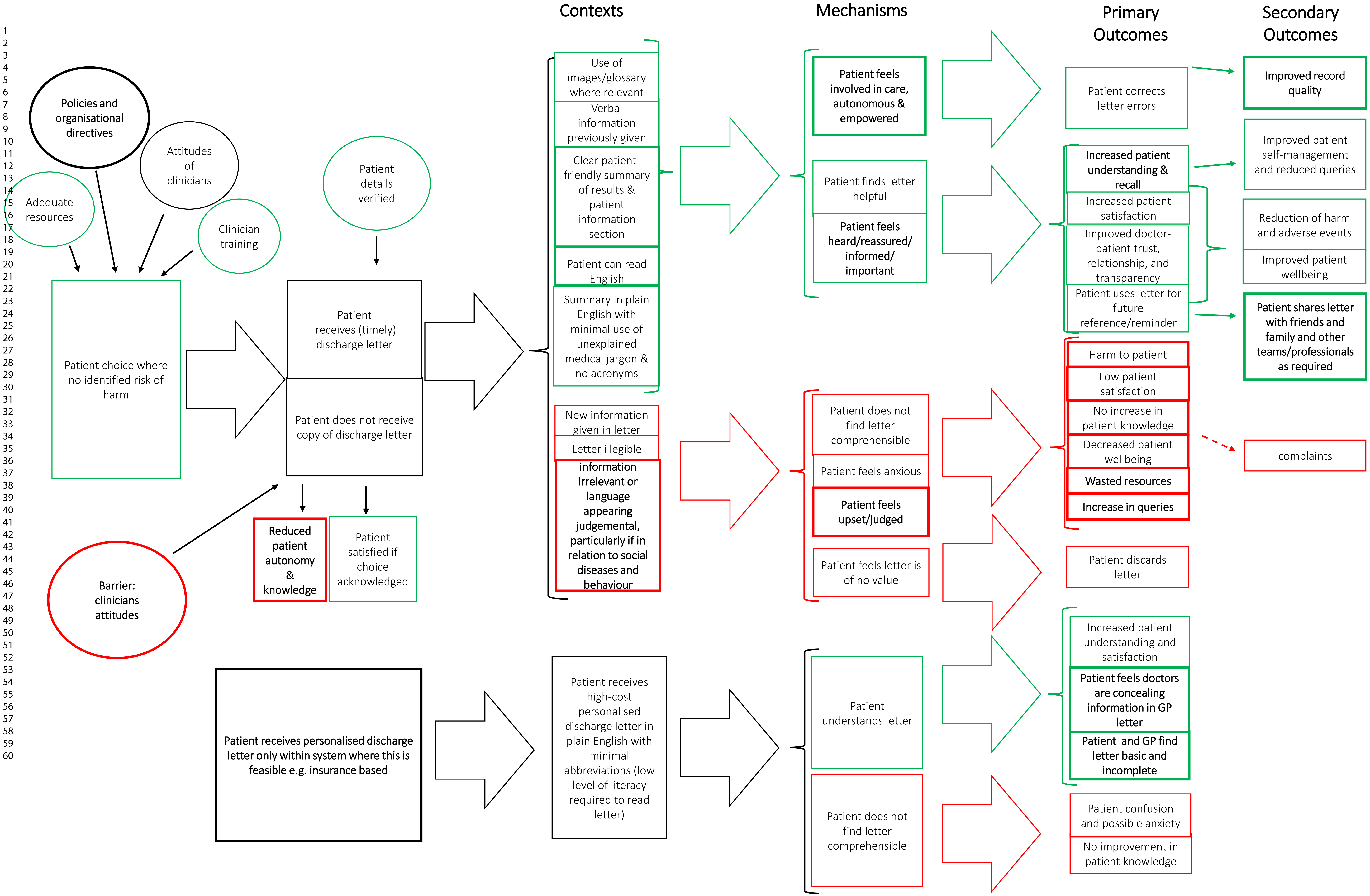

*Positive outcomes and positive pathway components are indicated in green coloured text boxes whereas negative outcomes and negative pathway components are indicated in red. Any neutral components or those which can be either positive or negative (e.g., attitudes of clinicians) are in black.

No. of letters selected	Patient name (to be removed during redaction)	Patient Unique research ID (to be added during redaction)	Categorisation (**Unsuccessful** OR **successful** discharge letter example)	Reason for selection & categorisation (e.g. any key good or bad points about letter)
EXAMPLE (Before redaction)<br><br>*(after redaction)*	*Mr Joe Smith*<br><br>[redacted]	 <br>*P0001*	*Unsuccessful*<br><br>*Unsuccessful*	*Bad points:*<br>*Medication alterations poorly outlined and information given to patient not explained*
1				
2				
3				

More rows to be added as needed…

*This grid has been previously published [1] under a CC-BY license and has been re-produced here for ease of reference for readers.

1.Weetman K., Spencer R., Dale J., *et al.* What makes a "successful" or "unsuccessful" discharge letter? Hospital clinician and General Practitioner assessments of the quality of discharge letters. *BMC Health Services Research*. 2021;21(1):349. https://doi.org/10.1186/s12913-021-06345-z [Accessed: 18/05/21].

For peer review only

GP interview and focus group guide

Interviewer opening question:

Please tell me about your experience(s) of patients receiving written discharge communication?

The rest of interview or focus group will continue in a conversational manner discussing GPs views and experiences on patients receiving written discharge communication and how the discharge communication process can be improved.

Possible interviewer prompts:

- What are your experiences of discharge communication as a GP?
- How do you think discharge communication can be improved?
- Please tell me your views on the discharge letters you selected?
- How would you suggest to improve these letters?
- In your opinions, is this letter suitable for a/the patient?
- What are your views on patients receiving letters?
- What do you think are important content items for good quality discharge letters?
- In your view what are the effects and outcomes of poor quality discharge letters?

*This guide has been previously published [1] under a CC-BY license and has been re-produced here for ease of reference for readers.

1.Weetman K., Dale J., Scott E., *et al.* The Discharge Communication Study: research protocol for a mixed methods study to investigate and triangulate discharge communication experiences of patients, GPs, and hospital professionals, alongside a corresponding discharge letter sample. *BMC Health Services Research*. 2019;19(1):825. https://doi.org/10.1186/s12913-019-4612-1 [Accessed: 10/06/20].

Patient interview schedule

I: Interviewer (member of the research team) *Action points Q= Question

I: ***Q1: Please tell me about your experiences of receiving any form of written discharge communication? This can be either a direct copy of the letter sent to your GP or a discharge letter specifically addressed to yourself.***

Q2: When you were discharged from hospital on DATE, what information were your given?

****if patient able to be shown letter copy as per protocol, show patient their letter****

Q3: How did you feel about the information you were given?

Q4: What written information would you like to be given or sent when being discharged from hospital and why?

Q5: Would you prefer to receive a direct copy of the letter sent to your GP or a separate letter specifically addressed to yourself?

Q6: Would you like to always be given this letter or would you prefer to choose each time you are discharged?

Q7: How do you think the process of patients receiving written discharge communication can be improved?

Q8: Is there anything else you would like to talk to me about today related to written discharge communication?

Discussion may continue in a relaxed conversational manner and researcher may ask additional questions related to anything else relevant mentioned by the patient.

*This guide has been previously published [1] under a CC-BY license and has been re-produced here for ease of reference for readers.

1.Weetman K., Dale J., Scott E., *et al.* The Discharge Communication Study: research protocol for a mixed methods study to investigate and triangulate discharge communication experiences of patients, GPs, and hospital professionals, alongside a corresponding discharge letter sample. *BMC Health Services Research*. 2019;19(1):825. https://doi.org/10.1186/s12913-019-4612-1 [Accessed: 10/06/20].

For peer review only - http://bmjopen.bmj.com/site/about/guidelines.xhtml

Trio meta-matrix with narrative summaries (S=successful, US=unsuccessful)

Trio case	GP grading	GP comment and interview/focus group findings	Patient interview findings	HP survey findings	Summary of main trio findings
1	S	Although GP graded letter successful due to clear diagnosis and findings, they did comment that the patient management plan was unclear. GP asserted that they felt patients should receive letters as it informs the patient and is a "safety net" for ensuring follow up plans are actioned.		HP gave letter high quality score of "9/9" and 9s in all other areas including GP care management plan except HP gave letter "4/9" for patient comprehensibility. HP concern that patients receiving letters may cause anxiety and distress. HP answered that it would be more appropriate for patients to receive personalised letters.	Although letter graded successful, GP did identify issues. Letter given a top score of "9" by HP. GP and HP appear to have differing views on whether patients should receive copies of their discharge letters with HP expressing concern and GP focussing on benefits.
2	S		Patient generally pleased with discharge experience and happy to have received copy of the letter. Patient likes to be informed. Patient suggests some issues with understanding medical terminology and says that they would prefer to receive patient personalised letter. Patient would prefer choice of receiving letter at discharge.	HP gave overall quality score of "7/9" and patient comprehensibility score of "9/9". HP reports to always copy patients into letters and believes patients should have choice of receiving letters. Answers that patients should receive GP copy of discharge letter.	HP and patient agree about patients receiving letters but appear to disagree over the form that this should take – patient favours personalised correspondence whereas HP favours patients receiving copies of what is sent to the GP.

For peer review only - http://bmjopen.bmj.com/site/about/guidelines.xhtml

Trio case	GP grading	GP comment and interview/focus group findings	Patient interview findings	HP survey findings	Summary of main trio findings
3	S		Patient overall seems pleased with communication and adds that they were given written and verbal information but only as they asked for a copy of the written information and that this was obtained after discharge. Patient describes follow up information in letter is unclear. Patient happy to receive copy of what GP receives and thinks it is reassuring to view the correspondence between doctors for transparency. Patient would prefer more detailed explanations in letter.	HP gives quality score of "8/9" with patient comprehensibility score of "9/9". Answers that patients should receive personalised letters and that patients should be given a choice. HP reports that despite hospital policy and their views on patient choice, they have never given a patient a discharge letter copy. HP believes that part of discharge letter should be given to patient and this is what is meant by personalised, not for two summaries to be generated.	HP given letter top score for patient comprehensibility but patient does report some issues and possible improvements which could be made to letter. Patient and HP in agreement over patient choice of receiving letters but disagreement over form.
4	S		Patient says they were impressed with information provided; they were given a discharge letter copy. Patient thinks patients should receive letters automatically.	HP gave overall letter score of "9/9" and patient comprehensibility score of "9/9". HP reports to give patients letters most of the time and thinks patients should receive GP copy in opt out style system.	Broad agreement between HP and patient within this trio case.
5	US	Unclear procedure due to acronyms not comprehensible to GP; for this reason, unclear what had been done. GP thinks abbreviations should be written out in full for clarity both for the sake of the patient and themselves.	Patient received letter after long discharge delay in hospital. Patient pleased to have received letter. Patient says they cannot understand all of letter but that they are aware they can ask the GP if they want to understand more.		Patient assumes GP understands all of letter and is a source of information for interpretation when GP does not due to use of uncommon abbreviations in letter.

Trio case	GP grading	GP comment and interview/focus group findings	Patient interview findings	HP survey findings	Summary of main trio findings
6	S	GP considered letter successful as follow up arranged. GP perceives use of acronyms in letter probably not comprehensible to patient. GP thinks use of lay terms in letter may be useful for patient understanding.	Patient thinks letter should ideally be emailed. Patient reports not being given much information and only received letter as relative went to hospital to get a copy after discharge. Patient feels discharge is not always clear and more time needs to be put in to ensure patient understanding. Patient felt letter generally inadequate and unsure of some of medical terms and acronyms in letter, patient states acronyms should not be used and terminology should be explained in lay terms.		GP and patient in agreement that letter format not entirely accessible to patient. Agreement over ways to rectify this issue through avoidance of acronyms and explanations of medical terminology in lay terms.
7	S	Letter graded successful as follow up clear. GP perceives letter written in patient friendly language.	Patient reports no difficulties with letter understanding but does note inaccuracies in letter.		GP and patient appear to agree on patient understanding.
8	US	Letter graded unsuccessful as drug changes and reasons for these unclear.	Patient reports being very pleased to have received copy of discharge letter having been given limited information in regard to previous discharges. Patient felt receiving letter supported their wellbeing. Patient conveys that receiving letter means that they can be actively involved in their own care and thus increase patient autonomy.		Patients receiving letters may support and improve patient wellbeing.
9	S	GP graded letter successful as it gave full details of investigations and findings and a working diagnosis. Important in GP view for patient to be given plan of action and instructions.	Patient reports not to have been given a copy of the letter. Patient would have preferred to have been given written information to ensure that they do not forget anything.		Patient and GP in agreement that patient did not receive a Letter and both appear to support practice of patients receiving letters.

For peer review only

Trio case	GP grading	GP comment and interview/focus group findings	Patient interview findings	HP survey findings	Summary of main trio findings
10	S	Letter graded successful as clear notes. Generally, letter informative and clear. GP raises possible issues with patient understanding due to presence of jargon and abbreviations; GP notes some patients would be fine with not understanding these elements whereas some patients will want to know more and may bring letter to GP with queries. GP says that there is a certain amount of technical information that needs to be passed between doctors but to improve patient understanding the letter should be clear and concise with use of lay language.	Patient given a copy of the letter. Patient reports medication information is very useful and clear but notes some issues with abbreviations for which they suggest an abbreviation chart. Patient suggests use of lay terms to make information clearer. Patient says receiving letter decreases the need to see the GP post-discharge.		GP and patient agreed that unexplained abbreviations may not be clear to patient and in order to increase patient understanding, acronyms and abbreviations should be spelt out in full and jargon should be accompanied by lay explanations.
11	S	Letter graded successful as detailed and clear plan. GP did note actions for patient and what the patient told unclear.	Received discharge letter. Patient suggestion that medical terminology could be better explained for patient. Suggestion that verbal explanatory information should accompany letter.		Patient felt in order to increase their understanding, jargon should be accompanied by lay explanations.
12	S	Letter graded successful as clear medication information and plan. Generally, GP happy with letter but is not sure how understandable this letter would be to patient. GP feels clinical summary and medication information would be useful to patient and that it is useful for patient to have a copy of the letter.	Patient received letter. Patient found letter information adequate and found medication information particularly useful. Patient felt information and detail in the letter was perhaps excessive and could be shortened and simplified.		GP and patient in agreement that discharge letter can usefully provide up to date medication information for patient. Patient felt letter contents could be simplified to increase its usefulness to them.

For peer review only

Trio case	GP grading	GP comment and interview/focus group findings	Patient interview findings	HP survey findings	Summary of main trio findings
13	S	Letter graded successful as clear medication information and follow up arranged. GP felt it was useful that letter says drugs started and stopped and reasons why. GP felt instructions to patient and follow up very clear. GP feels letter is appropriate and likely to be useful and comprehensible to patient.	Patient showed preference for receiving copies and did receive a copy in this case which they found useful. Patient liked that letter was simple and comprehensive but also brief. Suggestion that letter could be emailed to accelerate process.		GP and patient in agreement about letter usefulness and comprehensibility to patient.
14	S	Letter graded successful due to level of detail. GP reported issues with hospitals presuming GPs have access to system to view results when they often do not. Although GP graded letter successful, GP did comment that the letter would benefit from more information regarding the clinical summary and admission details. GP assesses letter as appropriate for patient.	Patient given discharge letter from hospital. Patient happy with this information, they felt it was clear what was wrong, what was going to happen next and medication information. Patient reports no problems with reading or understanding letter. Patient feels letter could have more detail. Patient thinks letter system should be opt out and patients should ideally receive personalised letters. Patient suggests use of lay terms to increase letter usefulness.		GP and patient in agreement about letter usefulness and comprehensibility to patient as well as level of detail for letter to be useful. Patient suggests use of lay terms to increase usefulness of letter to patient.
15	US	GP reports issues with the fact that the doctor writing the letter has not seen the patient. GP actions in letter described as ambiguous and inaccuracies noted by GP. The GP felt generally the letter is appropriate for the patient but raises concerns that the vague and unclear parts of the letter may cause patient anxiety. GP suggests how letter could meet needs of both GP and patient through simple interpretations of results and brief summarising of technical information to include breakdown of acronyms. GP felt unexplained acronyms should be avoided for the sake of patient understanding.	Patient not received letter and felt discharge communication process was poor. On letter review, patient was unclear on some of the medical terms in letter. Patient would have preferred to have been given copy of letter by hospital. Patient felt written discharge correspondence to patients should be mandatory.		GP suggests use of lay terms and simple interpretations to increase usefulness of letter to patient. Patient felt patient correspondence after discharge should be mandatory. GP felt acronyms should be avoided for the sake of understanding and clarity for patient. GP and patient in agreement that discharge communication unsuccessful.

For peer review only - http://bmjopen.bmj.com/site/about/guidelines.xhtml

Trio case	GP grading	GP comment and interview/focus group findings	Patient interview findings	HP survey findings	Summary of main trio findings
16	S	GP commented that patient not given a copy and they felt that the patient should have and that the letter would have been entirely appropriate for the patient. GP feels letter may have been reassuring for patient. GP comments that sharing letters with patients is the gold standard. Discharge plan simple and letter successful as concise and clear.	Patient reports being copied into recent letters but has found some of the letter contents technical. Despite this patient would prefer to receive copies of the letter sent to the GP rather than a patient personalised letter. Patient feels happy when they receive letters.		GP preference and patient preference for patients receiving letters. GP and patient disparity about whether or not patient received a copy of their recent discharge letter.
17	US	Letter graded unsuccessful as limited information regarding medication and investigations. GP found medication information unclear as well as working diagnosis. GP unsure whether or not letter wording would cause patient anxiety due to the diagnosis sounding serious. GP unsure whether letter language comprehensible to patient as many technical medical terms. GP thinks for safety netting, it is useful for the patient to know what the follow up plans are. GP reports information given to patients seems variable.	Patient says they were given discharge letter but with no accompanying verbal information or opportunity to ask questions. Patient reports feeling disappointed with discharge communication. Patient feels letter is not entirely accurate and that there have been ramifications as a result of this. Patient saw serious diagnosis for first time in letter which was slightly worrying.		GP and patient seem to be in agreement that discharge communication unsuccessful and that it is not ideal for the patient to be finding out about a potentially serious diagnosis for the first time in a letter with no accompanying counselling.
18	S	GP thinks patients need to know what is happening via a simple letter in lay language. Letter has handwritten pencil annotations which are unclear. Letter graded as successful due to good clinical summary and clear GP actions. GP concerns that receiving this letter may make patient feel anxious. GP raised issues with current prevalence of inaccuracies in discharge letters.	Patient says that they like to receive letters as they like to know what is going on. Patient feels discharge communication is good as long as they get a copy of the discharge letter.		GP and patient do not seem to be in agreement about patient appropriateness of letter. GP perceives letter may cause patient anxiety when the patient did not report this.

For peer review only - http://bmjopen.bmj.com/site/about/guidelines.xhtml

Trio case	GP grading	GP comment and interview/focus group findings	Patient interview findings	HP survey findings	Summary of main trio findings
19	S	Letter graded successful as clear diagnosis, summary, medication, diagnosis and plan. Nothing missing from the letter in GP view. To make letter clearer to patient, GP suggests jargon could be broken down and explained.	Patient happy to have received something written down so that they did not have to remember it. Patient mentions jargon not all initially clear but also says terms can be easily looked up on the internet or through other means. Patient likes to receive the same information as their GP.		GP concerned that patient may not understand letter and that letters such as this may need explaining. Patient happy to have received letter and notes resources such as internet that can be used to look up unknown terms.
20	US	Letter graded unsuccessful due to lack of medication details. Letter appropriate for patient only if they had knowledge of the information previously. GP thinks it is OK for patients to get copies as long as the letter is clear and meaningful to the patient otherwise the GP will need to spend time explaining letters to patients.	Patient seems somewhat indifferent to receiving letters and is most concerned that a copy is received by the GP. Patient would like to be given choice about receiving letter despite feeling that they often do not need a copy. Patient notes no faults with the letter.		Patient and GP disagree about quality of letter.
21	S	GP comments that letter is good quality and sufficiently detailed. GP feels generally letters are appropriate for patients and that it is useful for patients to have record of treatment and medications.	Patient values receiving letters and can understand them and finds them comprehensible. Broadly, patient impressed with letters they have received including the most recent.		GP and patient in agreement that letter suitable and useful for patient.
22	US	GP feels letter contains limited detail and no results of investigations or information regarding treatment. Due to lack of information, letter requires GP follow up to clarify details. GP unsure if this letter would be useful to a patient due to the lack of detail.	Patient pleased to have received copy of the discharge letter. Patient found letter very helpful. Patient prefers to receive copy of what is sent to the GP and unsure why anyone would want anything different. Patient cannot see way to improve letter.		GP and patient disagree on quality of letter.

Trio case	GP grading	GP comment and interview/focus group findings	Patient interview findings	HP survey findings	Summary of main trio findings
23	US	Letter grading due to the fact that the letter does not make sense to GP and is generally inadequate.	Patient likes receiving letters and to know what is going on. Patient reported no problems with letter or receiving it. Patient likes to receive a copy the same as what the GP receives.		GP and patient disagree on letter quality.
24	S	GP cannot think of case where it would not be appropriate for the patient to have a copy of the letter. GP believes patients receiving letters promotes and encourages autonomy and patient informed-ness and can also be reassuring. GP feels overall letter is clear and succinct.	Patient notes verbal and written information was conflicting. Patient pleased to have received letter and felt it was informative. Patent thinks patients need to know what happened, medication information and follow up plan. Patient feels letter system should be opt out to reduce the risk of patients mistakably not receiving letters.		GP and patient seem to agree on the benefits of patients receiving letters – that it can inform on condition and what is next.
25	S	GP expresses concerns with patients comprehending medical terms in discharge letters. GP does add that often patients having letters is useful particularly for GP home visits. GP expounds difficulty writing a letter to meet the needs of two audiences – GP and patient.	Patient reports being given limited information at the time of discharge. Patient notes a few inaccuracies on letter which made them feel uneasy about the rest of the letter and its accuracy, content, and quality. Broadly, patient did not feel the discharge experience was particularly good.		GP and patient slightly disagree on letter quality – GP grades as successful but patient does not describe communication and discharge experience positively.
26	S	GP graded letter successful as findings and plan clear. GP feels no new information should be communicated to the patient in the discharge letter. GP thinks that whether or not it is useful for patient to have a copy of the letter depends on the content and quality of letter. GP feels notes letters should never be handwritten as this can be unclear and thinks generally processes need improving to support better communication.	Patient reports being given limited information and no copy of the letter. Patient was left feeling slightly confused about what was going on. Patient would prefer to always receive copies of letter and for this to be the same as what the GP receives.		GP and patient in agreement that patient receiving letter can be useful.

For peer review only

Quartet meta-matrix with narrative summaries (*US=unsuccessful, S=successful)

Quartet case	GP grading*	GP comment and interview/focus group findings	Patient interview findings	HP survey findings	Main quartet findings
A	US	Letter graded unsuccessful by GP as diagnosis and reason for admission unclear as blank on letter template. GP unclear of cause of patient symptoms and presenting complaint and whether this cause is known to hospital. GP raises possible issues with patient understanding due to presence of jargon and abbreviations. GP thinks avoiding acronyms and use of lay terms in letter may be useful for patient understanding and notes that letter should be provided within context of adequate patient counselling. GP suggests patient information section on letter. GP feels template letters are good as they avoid things being missed. GP likes to know diagnosis, admission and discharge date, consultant details, medication, procedures and results, and patient awareness of diagnosis. GP feels blanks on summaries should not be permitted as unclear.	Patient received copy of letter but did not seem too pleased as they noticed inaccuracies on the letter which made them feel upset/angry. However, patient does find it useful to receive letter so that they can remedy discrepancies. Patient feels someone should go through letter with patients prior to discharge to reduce inaccuracies and ensure patient understanding. Patient prefers to receive direct copy of GP letter. Patient feels letter should have contained name of discharging physician.	HP gave overall letter a quality score of "6/9" with diagnosis information as "2/9" and patient comprehensibility as "2/9". HP felt patients should have a choice about receiving letters and that they should receive a GP copy. HP notes issues with letters being completed by most junior doctors, some of whom may not be on the corresponding consultant speciality team leading to issues. The HP comments that they tend to dictate letters which allows more information to be inputted as the template can be limiting.	Apparent agreement across all three groups that letter is somewhat unsuccessful. All groups raise issues with letter accuracy and HP notes this is likely due to junior status of completing doctor. GP and HP seem to agree patients should receive letter and patient agrees with this noting that had they not received the letter; they would not have been able to rectify the errors. Patient and GP agree that letter should be provided within the context of patient counselling.
B	US	GP comments that they have no way of knowing whether or not patient received letter. GP feels letter is not patient appropriate and could cause patient to feel anxious due to amount of medical language. GP adds that to improve letter, lay language for patient could be used. GP comments that it is good there are no handwritten sections on letter and that the findings are clear. GP feels patients need to know the procedure and results and follow up. GP comments that it is useful when patients receive letters because it helps them understand the action plan. GP feels that discharge letters need improving in terms of timeliness, factual accuracy, details regarding	Patient been given a copy of letter; it was in an unsealed envelope so they read it. Patient notes that follow up stated on letter has not happened. Patient notes they were lucky to have someone with them in hospital who remembered information as they did not due to effects of anaesthesia. Patient would have preferred interpretative simple summary of results. Patient mentions importance of considerations of the individual	HP gave overall quality score of "5/9" with patient comprehensibility score of "7/9". HP felt patients should receive choice of receiving letters and that this should be a GP copy. HP notes that they do not always have much time to complete discharge summaries and so must be brief. HP notes completing summaries which are timely but also informative and accurate is very challenging.	GP concerned that patient may not understand letter and that letters such as this may need explaining. Patient happy to have received letter and notes resources such as internet that can be used to look up unknown terms. Lower quality of letter perhaps explained by HP comments regarding the time pressures of completing summaries in their role.

For peer review only

Quartet case	GP grading*	GP comment and interview/focus group findings	Patient interview findings	HP survey findings	Main quartet findings
		what has happened, and plan of action. GP says that GPs are not responsible for chasing results and yet letters request this of them.	and patient choice. Patient notes that unfamiliar terms can easily be searched on internet.	HP notes that they feel their discharge letters are generally adequate but some HPs include only brief details.	
C	S	Successful grading as all information clear and concise including diagnosis and treatment plan. GP feels unexplained acronyms should be minimised for clarity for both GP and patient. GP notes inconsistency of patients receiving letters. GP raises concerns with patient understanding letter due to acronyms, one of which the GP is unfamiliar with, and medical terminology. GP feels that letter should clearly summarise the results in patient-friendly language to make content clearer (e.g. it should be stated that test results were normal for reassurance). GP feels the important items for letters are diagnosis, reason for admission, clinical summary, treatment and results, medication, and follow up and GP actions. GP feels letters are currently very variable in terms of quality. GP thinks patients should only not be given letters in cases of harm. GP comments that the "blank" GP action on letter is confusing and if there is no action this should be explicitly stated for clarity.	Patient has letter and notes that this is useful so if they go abroad they could show the letter to any clinicians looking after them as relevant. Patient notes that different patients may want different levels of information particularly in regard to bad news. Patient reports that they understand letter and are happy with it although they would have preferred to have been given a copy of the letter through the hospital rather than because they took part in the research. Patient suggests letter could be improved by being written in plain English. Patient notes the importance of adequate patient counselling. Patient values knowing next steps.	HP gives letter quality score of "8/9" across all categories to include patient comprehensibility. HP thinks patients should receive a choice of receipt and that the form should be personalised letters. HP rates their letters highly but adds no comments as to why.	GP expresses concerns regarding the patient understanding letter but patient notes that they did understand the contents. However, the GP and patient agree that the letter would be more useful if it was written in plain English with minimal or no acronyms. The HP seems unaware of the acronym issues. The HP feels patients would benefit from personalised letters but patient says they have preference for receiving a copy of what the GP receives. Letter seems to be evaluated as successful across population groups.
D	S	GP thinks patients receive letters variably. GP notes that language in letters is often very medical and so not suitable for the patient without explanation. The GP asserts that letters can be written in a straightforward way for the patient. GP feels patients should receive letters and says this can make patients feel more included in their care. GP feels letter is a bit brief in regard to results and follow up. Good elements of the letter are that tests have been overviewed. The GP feels a summary of the results to include interpretations	Patient says they did not receive a copy of the discharge letter but they would have liked one had it been offered. Patient would have preferred results to have been clearer and letter to make use of lay terms. Patient would like to be given letter every time they attend hospital. Patient suggests letter could be improved by clearer summary of	Letter given "1/9" by HP across quality scores. HP comments that the letter is poor because it was generated by a computer and was not written by themselves.. HP writes that the computer is unable to select the salient information and communicate it and so sometimes they send	HP and GP seem to agree that computerised templates are not particularly helpful. Groups broadly agree about letter quality. All groups agree patients should receive letters.

Quartet case	GP grading*	GP comment and interview/focus group findings	Patient interview findings	HP survey findings	Main quartet findings
		would be useful for the patient and the GP. The GP makes a general comment on the dangers of rapid hospital requests post-discharge.	what happened, medication, treatment, and follow up plans.	a separate letter to the GP with the important information.	
E	S	Letter graded successful as reason for admission and follow up plan were clear as were actions for GP and medication changes. GP favours that GP action in letter not blank but clear that the GP does not need to undertake further actions. GP feels the letter would be appropriate and useful to patient but may be improved by use of lay terms. GP notes patients receive letters inconsistently but they think it is useful for patients to receive copies particularly in regard to medication information. GP notes difficulty of writing letter that is patient friendly whilst meeting technical needs of GP. GP feels information in letter is quite medical and may be confusing/concerning for a patient; GP suggests lay explanations would help. However, GP does note letter would likely be useful for the patient so they are aware of the follow up plan. GP thinks important elements for letters are tests and results, diagnosis, GP action points. GP suggests patients are given abbreviated copies to include diagnosis, medications, and follow ups.	Patient reports that they had not received copy of letter but they would have liked to have done despite that the letter communicated bad news and a serious diagnosis. Patient would prefer copy of what goes to the GP and that this is useful so they can refer back to it so they are not dependent upon remembering information. Patient would like information in the letter relating to what happened and next steps.	HP rates letter “8” in all quality categories including GP information and patient comprehensibility. The HP notes producing summaries on a weekend when they are understaffed is a barrier to producing high quality letters. The HP feels their letter is clear and informative. The HP comments that the [hospital B] discharge templates are superior to the [hospital A] ones as they allow more freedom with inputting information.	The HP reports they always copy patients into letters and yet the patient reported they had not received a copy of the letter. There seems to be agreement across the groups that the letter was successful. GP expresses concern about patient understanding due to medical terms. The patient noted no understanding issues and found the letter useful.
F	US	Letter graded unsuccessful as unclear diagnosis and medication information. GP suggests that letter could be improved by medication information being put at the end of the letter rather than the beginning as this may cloud other important information. GP comments that positive aspects of the letter such as the inclusion of investigations, management plan, and actions for GP. Another letter improvement would be to specify if any blood tests need repeating and if so which ones and when. GP feels patients should receive letters.	Patient reports that they had received a copy of the discharge letter although one page missing when compared with GP copy. Patient found the medication information unclear. Patient also felt the diagnosis information was unclear and that they were given conflicting verbal and written information. The patient comments that they would like to receive a discharge letter every	HP grades letter an “8/9” for overall quality. HP notes restrictive template of summary can be a barrier to providing detail. The HP comments that upon reviewing the diagnosis it is unclear and they should/could have explained the presenting complaint better. The HP comments on the frustration that reports cannot be cut and pasted into the summary and	GP and patient seem to agree that letter requires improvements and that the medication information is unclear. All agree diagnosis information is unclear.

Quartet case	GP grading*	GP comment and interview/focus group findings	Patient interview findings	HP survey findings	Main quartet findings
			time they are discharged from hospital.	that the templates have restricting word counts.	
G	S	Discharge letter successful as it was concise with clear reason for admission, treatment, follow up, information given to patient, investigations and results. GP values that the medication changes in the letter are clear which is useful. GP thinks patients should receive letters but notes issues with jargon. GP feels current quality of discharge letters is variable and many letters have incomplete medication lists and insufficient detail regarding tests carried out and GP actions.	Patient reports being given copy of letter which they were happy with. Patient notes communication difficulties of being transferred between care providers. Patient felt medication information was a bit unclear and that when they were discharged, they still did not know the cause of their condition.	HP gives quality score of “6/9” and patient comprehensibility score of “3/9”. HP thinks patients should receive GP copies but not always. The HP comments that their spelling and grammar let them down but they do feel the management plan and diagnosis in the letter are succinct and informative.	Agreement between GP and patient as letter contained clear follow up and diagnosis but HP rates letter quality lower due their spelling and grammar mistakes.
H	US	Letter graded unsuccessful as no diagnosis and medication list incomplete. GP does note that there is a follow up plan which is helpful but without the diagnosis the letter is not clear enough. GP notes this letter does not contain enough detail. GP feels patients should receive letters but raises issues with unexplained medical terms. GP feels it is useful for patients to have record of medication and treatment. GP feels patient understanding could be improved through adequate patient counselling regarding discharge letter information.	Patient felt unclear of what the problem was when they discharged due to little information received. Patient reports that they did not receive a copy of the discharge letter but they would have liked to have done. Patient suggests that a patient personalised letter may be more valuable but that they would want both letters. Patient mentions use of internet for looking up unknown terms.	HP gives letter a “6/9” for quality and patient comprehensibility but rates diagnosis information a “2/9” as on reflection they feel this is unclear. The HP thinks the follow up information is also poor. HP thinks patients should receive GP copies and always be given a choice of receipt. The HP feels the letter could have been improved by specifying the differential diagnoses in light of the presenting complaint.	Diagnosis information indicated as unsuccessful across all three groups. GP raises issues with patients understanding medical terms. Patient mentioned no issues with letter contents and said that terms can easily be internet searched.
I	S	Successful grading as clear, inclusive of relevant information, and explained what information and advice given to the patient which the GP reports is not always included on summaries but very important. GP suggests issues with patients understanding letters particularly regarding medication changes and feels letters need to be written in plain English and lay language with	Patient reports to be given verbal information only and no letter which they did not find helpful. They would like to receive letters to include more detailed management and recommendations information. Patient wants letter to contain	HP gives scores of “9/9” for all categories except patient comprehensibility which they give “7/9”. HP claims to always copy patients into letters. HP commented that the letter was successful.	GP feels abbreviations need to be avoided in letters as these are not patient friendly. Patient and GP agreed that letter should be written in plain English with explained terms. GP and patient agree that patient actions and

For peer review only

Quartet case	GP grading*	GP comment and interview/focus group findings	Patient interview findings	HP survey findings	Main quartet findings
		minimal or no abbreviations. GP feels patients receiving letters is a good idea but needs to be accompanied by adequate patient counselling and letters should clearly highlight if the patient is required to take any action. GP notes that a successful letter is not a long letter.	specific information about what is wrong, medication, and how condition can be improved. Patient feels receiving verbal & written information is useful.		recommendations need to be explicit and clearer in the letter.
J	US	Unsuccessful grading due to lack of clear findings and follow up plan. GP feels the letter should have included clear details of the discharging physician and also information given to the patient alongside presentation of clinical findings. GP comments that the letter is particularly unclear as it is handwritten and illegible and so they feel uncertain of the exact procedure that the patient has had and the outcome. GP feels that this specific letter would not be helpful to the patient as it contains no information or advice or follow up details. GP also comments that the letter contains too many medical terms which would be hard for the patient to understand. GP notes general usefulness of patients receiving copies but says the letter should accompany counselling. The GP feels letters should always be typed.	Patient reports difficulties remembering the verbal information they were given as no letter. Patient was given a letter for the GP but as it was in a sealed envelope, they did not open it. Patient suggests they should have been given advice for condition and management, details of any follow up and medications, and expectations of recovery. Patient would prefer to receive a direct copy of what is sent to the GP and thinks patients should always be given letters as information can be easily forgotten.	HP gives letter quality score of “2/9” and notes it was actually produced by someone else more junior on their team but the letter has their name on. The HP rated the letter poorly across quality scales but did not provide any details as to how the letter could have been improved.	GP feels nothing in this particular letter would be of use to patient. Patient had trouble remembering the verbal information. Agreement across all three groups that discharge communication poor and unsuccessful. GP notes the illegibility of the letter due to handwritten form. The patient and HP focus on the content brevity. GP and patient agree that patient needs to know advice and follow up plans.

For peer review only

Table of Developed CMOCs (context, mechanism, outcomes configurations)

CMOC	Context	Mechanism	Outcome	Effect assessment	Does it "work" or not?
CMOC1	patient not offered letter	patient feels less involved in care	reduced patient autonomy	negative	does not work
CMOC2	patient offered opportunity to receive letter(s)/patient choice respected	patient feels more informed and involved in care	increased patient autonomy and increased involvement of patients in treatment, care and communications	positive	does work
CMOC3	large clear posters displaying patients right to choose and importance of correct contact information	patient realises they should inform hospital of address changes and preferences	lowered risk of confidentiality breach	positive	does work
CMOC4	NHS drive for patient-led care (influence or context)	clinicians increasingly offering patient choice of receiving letter/sharing information with patients	increased patient empowerment	positive	does work
CMOC5	clinician views letters to patients are beneficial e.g. increases transparency, compliance, trust, patient satisfaction, patient understanding and recall	clinician feels patient should be offered letter	potential increase in patient autonomy & satisfaction	positive	does work
CMOC6	Clinicians views letters to patients as not beneficial e.g. letter not comprehensible to patient, medico-legal issues, increased cost and staff workload, patient harm	clinician feels patient should not be offered letter	no patient autonomy	N/A	unclear

For peer review only

CMOC	Context	Mechanism	Outcome	Effect assessment	Does it "work" or not?
	(anxiety, distress, and confusion) and issues around confidentiality				
CMOC7	NHS guidance that all hospital-GP correspondence should be copied to patient as a "right" where appropriate and if patients agree (unless risk of serious harm or legal issues)	clinicians increasingly offering patient choice of receiving letter	increased use of NHS resources to send letters but patient benefits through increased understanding & potential reduction in patient queries (costs balanced)	positive	does work
CMOC8	Data Protection Act 1998 (UK)	Patients may become aware of their right to know what is written & stored about them	Patients informed of their stored electronic information (increased transparency)	positive	does work
CMOC9	doctor copies letters to patient	patient trusts doctor more	improved doctor-patient relationship	positive	does work
CMOC10	patient offered choice of receiving letters	patient chooses to receive letters	Increased administrative staff workload and costs of printing & posting letters	negative	unclear
CMOC11	patient offered choice of receiving letters	patient chooses to receive letters	reduced queries and GP visits and reduced hospital re-admissions (limited evidence)	positive	does work
CMOC12	structured discharge letters written clearly in plain English (pref. 5th grade level) with medical jargon explained with lay terms, no value judgements of patients and minimal abbreviations	patients understand letter	increased patient knowledge	positive	does work

For peer review only

CMOC	Context	Mechanism	Outcome	Effect assessment	Does it "work" or not?
CMOC13	doctors provided training in letter writing & record keeping (contextual influence) leading to doctors write letters of higher quality and more appropriate for patients	patients understand letter	Increased patient knowledge/potential increase in doctor confidence in letter writing	positive	does work
CMOC14	patient preference for letter copies acknowledged and patient offered choice of receiving letter	patients feel able to express their preference	decreased strain on resources & increased patient autonomy & satisfaction	positive	does work
CMOC15	patient provided written & verbal information to include sufficient counselling	patient reflects on written record of information for reference	increased patient knowledge of care plan, recall and acceptance of illness or condition	positive	does work
CMOC16	Human Rights Act (1998) and Race Revelations Act (2000) - clinicians equally offer all patients letter copies regardless of background	clinician feels all patients should be offered letter	increased equality and accessibility of information to patients	positive	does work
CMOC17	Use of pictures/pictographs/cartoons with written information	patients understand letter	Patient benefits from improved understanding e.g. adherence to agreed care plan	positive	does work
CMOC18	verbal information only	patient may not be able to retain information	reduced patient recall	negative	does not work
CMOC19	professionals who are not involved/limited involvement with patient writes letter	professional does not understand patient plan	letter quality reduced/increased risk of harm	negative	does not work
CMOC20	patient hospital visit of sensitive nature and/or patient lacks capacity e.g. psychotic episode, dementia	patient finds letter distressing and/or confusing	harm to patient	negative	does not work

For peer review only

CMOC	Context	Mechanism	Outcome	Effect assessment	Does it "work" or not?
CMOC21	Patient letter written above patient educational level or in a language the patient does not read	patient finds letter difficult to understand	patient is confused with no increased knowledge of care/possible misinterpretation of care instructions	negative	does not work
CMOC22	letter contains inaccurate information	patient identifies inaccuracies	patient notifies hospital/GP of inaccuracies and corrections are made leading to improved record keeping	positive	does work
CMOC23	patient receives discharge letter	patient does not understand entirety of letter	patient sources answers (internet, GP, friend or relative)	positive	does work
CMOC24	Patient specific letter sent to patient	patient finds letter clear	improved patient comprehension	positive	does work
CMOC25	Patient specific letter sent to patient	Clinician produces two letters	increased staff workload and costs	negative	does not work
CMOC26	Patient specific letter sent to patient	Patient identifies information sent to GP and patient is different	medico-legal concerns could be raised over letter discrepancies and any withheld information	negative	does not work
CMOC27	hospital sends patient discharge letter without verifying patient contact details without notifying patient	hospital worker does not identify and correct incorrect information	potential breach of patient confidentiality	negative	does not work
CMOC28	hospital routinely checks patient addresses and sends discharge letters to patient marked confidential using full name	hospital worker identifies and corrects incorrect information	patient receives letter, minimal risk of patient confidentiality breach	positive	does work
CMOC29	patient receives discharge letter	patient may feel they have questions relating to letter	patient contacts health provider with queries (evidence suggests minimal impact and queries)	positive	unclear

For peer review only

CMOC	Context	Mechanism	Outcome	Effect assessment	Does it "work" or not?
CMOC30	discharge letter/summary dictated in front of patient	patient queries any inaccuracies	letter less likely to contain inaccuracies	positive	does work
CMOC31	Hospital gives patient letter to deliver to GP	patient may find they are unable to make delivery or patient does not like being asked to perform this task	GP does not always receive letter. Patient satisfaction low.	negative	does not work
CMOC32	Patient receives letter not written at appropriate level for them	patient does not understand letter	patient feels confused and dissatisfied with discharge care	negative	does not work
CMOC33	Patient has anxiety that doctors talk about things behind their backs	patient who receives letter feels reassured that there is no hidden information	decreased patient anxiety and improved doctor-patient relationship through transparency	positive	does work
CMOC34	patient receives discharge letter	Patient feels they are important to clinician	patient is impressed with letter and feels clinician has an interest	positive	does work
CMOC35	choice about whether letter is sent to patient	clinician feels letters would be a disaster and inappropriate for patient	patient does not receive letter(s)	N/A	unclear
CMOC36	patient receives discharge letter	Patient feels indifferent	no impact on patient	N/A	unclear
CMOC37	patient receives discharge letter with bad news	Patient finds letter initially distressing	letter causes initial distress but final outcome that patient finds letter helpful and aids recall and acceptance of condition	positive	does work
CMOC38	letter sent to patient containing information not discussed with patient or abnormal results	patient feels distressed and anxious reading letter	patient harm/unethical practice	negative	does not work
CMOC39	patient worried about diagnosis and receives letter	patient understanding helped by letter	patient feels less anxious due to being more informed	positive	does work

For peer review only

CMOC	Context	Mechanism	Outcome	Effect assessment	Does it "work" or not?
CMOC40	patient preference for letter copies not acknowledged	Patient dissatisfied to have received letter	decreased patient satisfaction	negative	does not work
CMOC41	patient offered choice of receiving letters (opt out)	patient enabled to decide on letter preference	patient may or may not receive letter depending on their preference in relation to the particular care episode resulting in higher patient satisfaction. Increased rate of patients receiving letters	positive	does work
CMOC42	patient who feels copies of letters are not necessary for themselves	Patient pleased not to be given letter	patient satisfied, secondary outcomes: costs and time saved	positive	does work
CMOC43	patient receives discharge letter where appropriate	patient understands letter	patient finds letter informative and helpful. Patient wellbeing boosted and supported	positive	does work
CMOC44	patient receives discharge letter where appropriate	patient feels involved in care plan	patient ensures follow up plan is followed and books any necessary tests etc.	positive	does work
CMOC45	patient receives discharge letter where appropriate	patient feels letter is important	letter forms permanent record of hospital visit and kept for future reference. Patient may show letter to family and friends.	positive	does work
CMOC46	patient receives discharge letter for breaking good news	patient reminded of discussion	patient feels reassured and has "peace of mind"	positive	does work
CMOC47	patient receives discharge letter where appropriate (patient choice)	patient likes receiving letter	patient satisfaction increased	positive	does work
CMOC48	patient receives copy of discharge letter where appropriate	patient becomes aware of what GP knows	Patient reassured that GP knows about visit	positive	does work

For peer review only

CMOC	Context	Mechanism	Outcome	Effect assessment	Does it "work" or not?
CMOC49	Tickbox/template allows letter copies to patients to be monitored and audit trailed	HP becomes aware of practice of copying patients letters	Increased practice of patients receiving letters. Inconsistencies can be monitored for improving uptake.	positive	Does work
CMOC50	Letter acts as record of consultation and given to patient	Patient reminded of consultation	Patient recall increased and no need for patient to remember all consultation information	positive	Does work
CMOC51	Letter acts as record of consultation and given to patient	Patient prompted to use letter for administrative proceedings without need to contact GP or hospital	Letters can be used as proof of illness for benefit receipt, government support, disability applications and allowances, or time off work.	positive	Does work
CMOC52	Patient episode of care due to repeat or ongoing condition	Patient feels already informed about condition	Patient chooses not to receive letter preserving resources	positive	Does work
CMOC53	Patient receives letter with irrelevant or poorly phrased social disease or behaviour details	Patient feels judged and upset	Patient reflects on episode of care poorly and wellbeing negatively impacted	negative	Does not work
CMOC54	Letter provided to patient with additional patient information section	Patient understands summary	Patient knowledge increased and patient reassured that the important content points have been communicated.	positive	Does work
CMOC55	Clinician concern about patient understanding letter	Patient feels they do understand letter	Clinician concerns potentially unfounded. Patient values receiving letter	positive	Does work

For peer review only

COREQ (COnsolidated criteria for REporting Qualitative research) Checklist

A checklist of items that should be included in reports of qualitative research. You must report the page number in your manuscript where you consider each of the items listed in this checklist. If you have not included this information, either revise your manuscript accordingly before submitting or note N/A.

Topic	Item No.	Guide Questions/Description	Reported on Page No.
**Domain 1: Research team and reflexivity**			
Personal characteristics			
Interviewer/facilitator	1	Which author/s conducted the interview or focus group?	
Credentials	2	What were the researcher's credentials? E.g. PhD, MD	
Occupation	3	What was their occupation at the time of the study?	
Gender	4	Was the researcher male or female?	
Experience and training	5	What experience or training did the researcher have?	
Relationship with participants			
Relationship established	6	Was a relationship established prior to study commencement?	
Participant knowledge of the interviewer	7	What did the participants know about the researcher? e.g. personal goals, reasons for doing the research	
Interviewer characteristics	8	What characteristics were reported about the inter viewer/facilitator? e.g. Bias, assumptions, reasons and interests in the research topic	
**Domain 2: Study design**			
Theoretical framework			
Methodological orientation and Theory	9	What methodological orientation was stated to underpin the study? e.g. grounded theory, discourse analysis, ethnography, phenomenology, content analysis	
Participant selection			
Sampling	10	How were participants selected? e.g. purposive, convenience, consecutive, snowball	
Method of approach	11	How were participants approached? e.g. face-to-face, telephone, mail, email	
Sample size	12	How many participants were in the study?	
Non-participation	13	How many people refused to participate or dropped out? Reasons?	
Setting			
Setting of data collection	14	Where was the data collected? e.g. home, clinic, workplace	
Presence of non-participants	15	Was anyone else present besides the participants and researchers?	
Description of sample	16	What are the important characteristics of the sample? e.g. demographic data, date	
Data collection			
Interview guide	17	Were questions, prompts, guides provided by the authors? Was it pilot tested?	
Repeat interviews	18	Were repeat inter views carried out? If yes, how many?	
Audio/visual recording	19	Did the research use audio or visual recording to collect the data?	
Field notes	20	Were field notes made during and/or after the inter view or focus group?	
Duration	21	What was the duration of the inter views or focus group?	
Data saturation	22	Was data saturation discussed?	
Transcripts returned	23	Were transcripts returned to participants for comment and/or	

For peer review only

Topic	Item No.	Guide Questions/Description	Reported on Page No.
		correction?	
**Domain 3: analysis and findings**			
Data analysis			
Number of data coders	24	How many data coders coded the data?	
Description of the coding tree	25	Did authors provide a description of the coding tree?	
Derivation of themes	26	Were themes identified in advance or derived from the data?	
Software	27	What software, if applicable, was used to manage the data?	
Participant checking	28	Did participants provide feedback on the findings?	
Reporting			
Quotations presented	29	Were participant quotations presented to illustrate the themes/findings? Was each quotation identified? e.g. participant number	
Data and findings consistent	30	Was there consistency between the data presented and the findings?	
Clarity of major themes	31	Were major themes clearly presented in the findings?	
Clarity of minor themes	32	Is there a description of diverse cases or discussion of minor themes?	

Developed from: Tong A, Sainsbury P, Craig J. Consolidated criteria for reporting qualitative research (COREQ): a 32-item checklist for interviews and focus groups. *International Journal for Quality in Health Care*. 2007. Volume 19, Number 6: pp. 349 – 357

Once you have completed this checklist, please save a copy and upload it as part of your submission. DO NOT include this checklist as part of the main manuscript document. It must be uploaded as a separate file.

For peer review only
